# Understanding DFT Uncertainties for More Reliable Reactivity Predictions by Advancing the Analysis of Error Sources

**DOI:** 10.1021/acs.jctc.5c00985

**Published:** 2025-09-18

**Authors:** Gergely Laczkó, Imre Pápai, Péter R. Nagy

**Affiliations:** † Institute of Organic Chemistry, 280964 HUN-REN Research Centre for Natural Sciences, Magyar Tudósok Körútja 2, Budapest H-1117, Hungary; ‡ Hevesy György PhD School of Chemistry, Eötvös Loránd University, P.O. Box 32, Budapest H-1518, Hungary; § Department of Physical Chemistry and Materials Science, Faculty of Chemical Technology and Biotechnology, 61810 Budapest University of Technology and Economics, Müuegyetem rkp. 3., Budapest H-1111, Hungary; ∥ HUN-REN-BME Quantum Chemistry Research Group, Müuegyetem rkp. 3., Budapest H-1111, Hungary; ⊥ MTA-BME Lendület Quantum Chemistry Research Group, Müuegyetem rkp. 3., Budapest H-1111, Hungary

## Abstract

Decades of advancements and thousands of successful applications have contributed to the reliability of density functional theory (DFT) methods. Especially in main group chemistry, DFT predictions tend to be increasingly more reliable. In this study, we deeply analyze unexpected (ca. 8–13 kcal/mol) DFT disagreements obtained for a few organic reactions using only widely adopted, modern, hybrid, and higher-rung DFT methods. To understand the underlying causes, we move beyond conventional statistics-based benchmarks by combining recent advances in DFT error decomposition with affordable gold-standard references. This approach helps to characterize and disentangle multiple functional and density-based error types and enables us to find functional(s) suitable for broad mechanistic studies in all studied examples. The proposed tools are cost-efficient, readily accessible, and easy to integrate into routine thermochemistry workflows. While the focus is on main group reactions, the approach is also applicable to transition metal, bio-, and surface chemistry to assist more predictive reactivity modeling.

## Graphical abstract

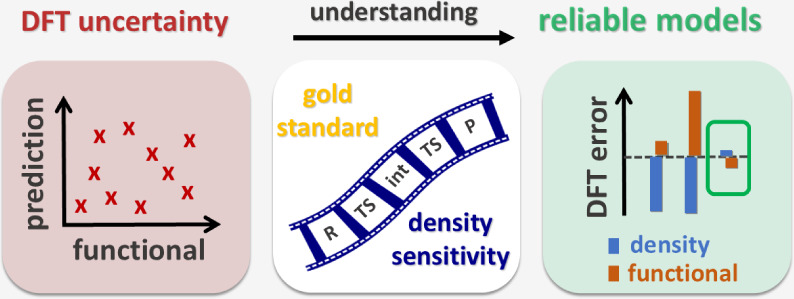

## 1 Introduction

Computational modeling of chemical reactivity and catalysis with quantum chemical^1–6^ and recently also with data-driven and machine-learning methods^7–10^ is well established and successfully exploited, very often in synergy with synthetic developments. The current capabilities of these computational tools, with DFT having a central role, are well documented.^10–19^ These recent reviews suggest a shift in the main challenge of predictive modeling from the electronic structure problem to other important aspects associated with the effects of finite temperature and environment, as well as competing reaction pathways and conformational complexity.^10–19^

Regarding the electronic structure problem, density functional approximations often perform well and their potential shortcomings are also increasingly more understood. Current warnings about difficulties for DFT focus on the clearly identified issues (multireference character, transition-metal and open-shell species, etc.).^17,20,21^ When these issues are avoided, especially for homogeneous (catalytic) reactions in main group chemistry, the consensus expects reliable DFT performance.^11–13,16,20,22,23^ Unlike this general trend and our own experience, here, we study unexpected DFT inconsistencies obtained for the synthetically relevant and representative organic reactions in Figure 1.^24–26^ A spread of 8–13 kcal/mol remains even if we look at only advanced, hybrid and higher rung functionals, take out the largest negative and positive errors from *a priori* reasonable DFT models, and focus on some of the most popular functionals (e.g., see colored markers for ωB97X-D, B3LYP-D3, M06–2X on Figure 1).

**Figure 1:**
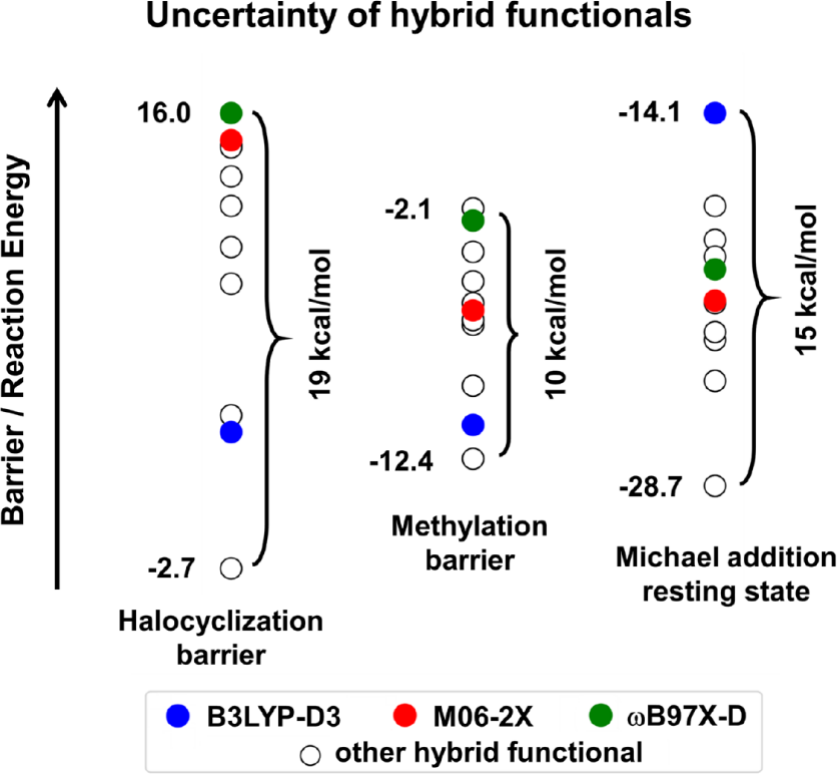
Range of DFT uncertainties at least at the hybrid DFT level in three organic reactions (detailed below in Section “Results and Discussion”). The plotted stabilities were obtained with advanced and widely adopted functionals (listed in Table S2). For clarity, the energies are represented with empty dots and only 3 very popular methods (B3LYP-D3, M06–2X, and ωB97X-D, respectively) are highlighted with colored markers.

The aim of this study is to understand the underlying causes of these discrepancies, thereby aiding future DFT development and the selection of reliable DFT approximations in practice for such unclear situations. In general, indispensable assistance is given to DFT model selection by statistical analysis in broad benchmark studies^27–30^ and reviews of best practices.^15,20,23,31^ These works usually echo the advice to assess multiple DFT models until reaching a consensus. Our study addresses the rather underexplored situation when a deeper understanding is required as one cannot follow the strategy based on the agreement among the most trusted DFT models (Figure 1) or to limited benchmark data.

Besides experimental references, wave function based benchmarks, such as the coupled cluster (CC) model with single and double and perturbative triple excitations [CCSD­(T)],^32^ are increasingly employed. CCSD­(T) is considered a “gold standard” method because its chemical accuracy (ca. 1 kcal/mol uncertainty) has been repeatedly corroborated^33^ when remaining within its applicability domain.^34^ Current local correlation methods enable, albeit at an order of magnitude higher cost than hybrid DFT, relatively routine access to such CCSD­(T) energies, as reviewed recently in ref.^35^ However, structure optimization, thermochemical corrections, spectroscopic properties, etc. are far from available at the local CCSD­(T) level, and thus computational studies will require reliable DFT methods for a long time. Moreover, even when some CCSD­(T) (or experimental) references are available, it can still be challenging to choose a suitable DFT method, as also illustrated in the examples presented here or for a multistep organic cycloaddition reaction.^36^ As Truhlar, Frisch, Adamo and coworkers have most recently highlighted,^36^ for complicated DFT error patterns, “it would be of great interest to understand why some functionals are more accurate than others”, but current considerations still “do not enable one to see which functionals will have acceptable accuracy for a given complex mechanism”.

This current conclusion^36^ is also in line with our aim here for a more systematic approach to better understand DFT uncertainties, especially due to multiple sources of errors, and to assist in reliable model selection for practical, complicated reactions. To that end, we adapt and combine advanced DFT analysis and CCSD­(T) methods, which recently became ready for widespread use. Namely, we

1 exploit local correlation based CCSD­(T) energies^37–39^ with well-converged approximations and robust uncertainty estimates,^35^
2 combine this with recent DFT error decomposition and density error estimation methods,^40,41^ and
3 go beyond the current benchmark approaches often focusing only on the statistical analysis of DFT energy errors by separating and characterizing the sources of DFT errors from reactants, through barriers and intermediates to products.

The benefit is that the combination of (1)-(3) [or when sufficient already (1) and (2)] enables the informed choice of DFT methods for better reasons, e.g., by using models specifically designed to mitigate the identified types of DFT errors.

This idea builds on one of the major DFT development approaches, that is to recognize general DFT limitations and then to design models overcoming them.^42–44^ An outstanding recent success along these lines is the development of dispersion corrections.^45–48^ Another extensively researched but not yet as well resolved general issue is connected to errors in the DFT densities, often due to the self-interaction error (SIE).^40,44,49,50^ The textbook examples for SIE are one electron systems, where (for fully polarized systems) the Hartree–Fock (HF) model is exact. SIE emerges when the Coulomb and exchange components of DFT methods do not cancel completely, leading to a nonphysical interaction of the electron with itself.^51^ Promising methods have been under development to overcame one-electron SIE^52–55^ and more general many-electron SIE and delocalization error.^44,50,56^

The resulting overly delocalized densities can affect a wide range of applications, including bond dissociation and torsion barriers, σ-hole interactions, radical and ionic complexes, etc.^40,44,49,50^ Consequently, the accuracy of DFT densities and SIE are still in the center of intense scientific discussions.^40,57–60^ For example, the idea of replacing the potentially SIE-prone self-consistent DFT density with its SIE-free HF counterpart, that is HF-DFT, is an early concept^61–63^ which was systematically revisited by Burke and coworkers.^40,41^ The proposal to use HF-DFT when the density error can be expected to be severe offers a successful remedy for a wide range of SIE-prone systems listed above.^40,41,64^ However, HF-DFT was shown to sometimes benefit from compensation of errors or be outperformed by some hybrid or higher rung functionals in general purpose test sets.^59,60,65^

Nevertheless, the HF-DFT line of studies by Burke et al. also introduced a key concept of decomposing the total DFT error to functional and density-driven error components^66^ and a practical density sensitivity measure to estimate the latter^67^ (as detailed in Section 2). Here, we show that these tools are useful by themselves, however, so far very little was known about their behavior for chemical reactions of synthetic relevance. For example, regarding the performance of HF-DFT and density sensitivity on processes along a reaction coordinate, only simple systems, such as $H_{2}^{+}$ , NaCl, and FCl···NH_3_ dimer dissociation were investigated.^40,41^ Somewhat more is known about density sensitivity measures for transition states (TS) of, e.g., small molecule reactions, like H· + H_2_/HF or CH_3_Cl + F^–^.^40,41,68^

Thus, to transfer these tools from the domain of textbook systems to routine applications, our study also provides better understanding on how the functional and density error components behave for more complicated, practical reactions. All methods employed here affordably fit into existing reaction mechanism exploration protocols and are sufficiently simple and (openly) accessible in multiple quantum chemistry packages.^69–71^ This makes the suggested tools readily and widely applicable. We demonstrate this on the practical reactions of Figure 1 with current synthetic relevance in main group chemistry. Namely, C–C and C–O bond as well as ring formation reactions via halocyclization,^24^ methylation,^25^ and Michael addition^26^ are analyzed in detail.

All of these investigated systems exhibit at least two different kind of DFT issues in a single reaction, highlighting the benefits of the proposed advancements, i.e., the ability to disentangle and understand the potentially confusing (like in Figure 1) interplay of multiple causes. While at the moment it is unclear how rare are such modeling uncertainties in organic chemistry, caution is advised due to the relatively broad occurrence of the chemical motifs that are found to cause them. In such cases, the suggested computational approach enables a deeper understanding and thus more predictive DFT choices for better reasons.

## 2 Methodology

### 2.1 Accurate CCSD­(T) References

The DFT uncertainties will be measured against well-converged CCSD­(T) reference electronic energies using the efficient local natural orbital (LNO),^35,37–39,72^ method of the Mrcc program package.^69,73,74^ Relying on systematically improvable series of basis sets and local approximation settings, as well as on corresponding extrapolation toward the complete basis set (CBS) and the local approximation free (LAF) limits, gold standard CCSD­(T)/CBS results can be approached within chemical accuracy (1 kcal/mol).^35^ Moreover, the remaining local and basis set errors in the LNO–CCSD­(T) references with respect to exact CCSD­(T)/CBS can be characterized using robust error estimates.^35^ The combination of CBS and LAF extrapolations with LNO–CCSD­(T) takes advantage of tightly converged LNO and quadruple-ζ basis set levels, which are broadly accessible within ca. half a day on 16 cores, even for the largest system studied here (**B-ts**). If converged properly, other local correlation methods could also be used to get the same reference energies.^35,75–77^ Further technicalities are presented in Section S1. The SI reports details of the LNO–CCSD­(T) reference computations, careful LNO–CCSD­(T) convergence studies and error estimates including CBS extrapolations up to diffuse quintuple-ζ bases, LAF extrapolations from up to very Tight LNO settings and canonical CCSD­(T) benchmarks.^35,78^ These tests show 0.1–0.3 kcal/mol local and basis set uncertainties, which is clearly suitable to assess DFT methods in the present study.

### 2.2 DFT Error Analysis: Density Component

In principle, the total DFT error with respect to the exact electronic energy can be decomposed into density-driven (Δ*E*_dens_) and functional (Δ*E*_func_) error components:^66^

1 $$\Delta E=E^{\mathrm{DFT}}\left[\rho^{\mathrm{DFT}}\right]-E\left[\rho \right]=\Delta E_{\mathrm{dens}}+\Delta E_{\mathrm{func}}$$

Here, the density-driven error in the energy of a density functional approximation (*E* ^DFT^) is the energy difference obtained with its self-consistent density (ρ^DFT^) and the exact density (ρ):

2 $$\Delta E_{\mathrm{dens}}=E^{\mathrm{DFT}}\left[\rho^{\mathrm{DFT}}\right]-E^{\mathrm{DFT}}\left[\rho \right]$$

Then, the remaining functional error is the difference between the exact energy of the exact functional (*E*) and the energy of the approximate functional, both evaluated on the exact density:

3 $$\Delta E_{\mathrm{func}}=E^{\mathrm{DFT}}\left[\rho \right]-E\left[\rho \right]$$

Since the exact electronic energy, density, and density functional are not accessible we employ practical approximations. Namely, Δ*E* is obtained against the LNO–CCSD­(T) reference and we estimate Δ*E*_dens_ using the density sensitivity measure employed by Burke et al:^67^

4 $$S^{\mathrm{DFT}}=E^{\mathrm{DFT}}\left[\rho^{\mathrm{LDA}}\right]-E^{\mathrm{DFT}}\left[\rho^{\mathrm{HF}}\right]$$

Here, *S* ^DFT^ is the difference of the approximate DFT energy obtained with two densities: the local density approximation (LDA) density, which is one of the most sensitive to SIE, and the Hartree–Fock (HF) density, which is free from SIE by definition. Thus, *S* ^DFT^ basically measures how sensitive the selected functional is to the SIE in the density (and thus not related to the functional component of the SIE.^79,80^ When *S* ^DFT^ is large, one expects that the self-consistent density of the corresponding functional can cause sizable energy errors. In turn, a small *S* ^DFT^ measure indicates the insensitivity of the functional to SIE in the density. In a slight deviation from the density sensitivity definition of ref. 67 where the absolute value of eq 4 is used, we find here that the signed density sensitivities are simpler to interpret and more informative for our purposes.

We note here, that for DH functionals used in practice, the density is optimized with a (hybrid) functional different from the functional used for the energy evaluation, which then depends on also the KS orbitals and orbital energies. Thus, the analysis of density sensitivity is not sufficient to explore the error sources for DH methods,^81^ and we do not present density sensitivity measures for them. Nevertheless, one can expect that modern DH methods are not as sensitive to SIE as their HFx content is usually above 50%.

### 2.3 DFT Error Analysis: Functional Component

The remaining, functional error can be characterized via the above approximations for Δ*E* and Δ*E*_dens_ utilizing Δ*E*_func_ = Δ*E* - Δ*E*_dens_ [from eq 1]. The practical benefit is clear as other tools to assess the quality of the functional, such as the Kohn–Sham inversion, are yet not affordable due to, e.g., the cost of wave function based densities.,^59,82,83^ While investigating Δ*E*_func_ this way could often be sufficient, if needed, we can go further and analyze some of its components originating from the approximate exchange and correlation functionals. Regarding the importance of the dispersion component,^45–48^ one can look at the size of the dispersion correction (see Table S2). These, as well as, second-order (MP2) and CCSD­(T) correlation energy contributions inform us about the size and complexity (e.g., in terms of perturbation order) of the electron correlation effects. Then, these measures indicate the level of difficulty faced by the correlation functional for a specific chemical process. To analyze the system-specific effect in the exchange component, we can systematically vary the portion of exact HF exchange in the functional.

While the indicators collected in the previous paragraph are relatively simply accessible, it is also insightful to first inspect trends along the rungs of Jacob’s ladder.^84^ Here, we employ 24 functionals covering the top four rungs of Jacob’s ladder, including some of the most popular and accurate functionals of each rung and multiple categories with no, moderate, and a high number of empirical parameters (see Section S1 and Table S2). Here, we will focus on hybrid (H) functionals and higher rungs, including HF exchange (HFx), as their use can be considered standard in reaction mechanistic studies. Generalized gradient approximation (GGA) and meta-GGA (mGGA) functionals are nowadays employed mostly for molecular dynamics, condensed phase calculations, or structure optimization of very large molecules. Including briefly (m)­GGAs as well is informative for us, e.g., for the purpose of making the error sources related to the density more apparent. Due to the importance of SIE in the present study and with the expectation of smaller SIE with increasing HFx content, we divide the hybrids into two groups with lower and higher than, say, 40% HFx content. Analogously, range-separated hybrids (RSHs) are assigned to a separate category, as they usually include a very high amount of (or in some cases even 100%) long-range HFx. Finally, functionals from the double-hybrid (DH) rung are also included providing, in general, the most accurate energetics on the highest rung. However, despite accelerated DH approaches,^85,86^ the cost of DH gradients and Hessians is still too high for larger molecules. Thus, we focus on finding (RS)H methods with reasonable accuracy over cost performance, which can be recommended for routine use in reaction mechanism modeling.

### 2.4 Tracking the Components along the Reaction Coordinate

If multiple sources of error are found significant, we move beyond evaluating functional performance only at stationary points, which may obscure the potentially complex interplay of errors. Since different error types can vary throughout the reaction, sometimes canceling or amplifying one another, we extend the analysis along the reaction coordinate (RC). This helps to disentangle the error types, as there are regions along the RC where certain error sources become negligible, enabling us to isolate and identify the dominant ones. Then, starting from the point(s) where there is only one clearly identified error type, we can track the changes along the RC to structures where the error pattern is more involved. Specifically, we examine how the DFT errors and density sensitivities vary along the RC using the same structures for all methods (as detailed in Section S1). In this study, besides the popular M06-2X-D3, ωB97X-D4, and B3LYP-D4 functionals, we analyze more closely a an additional (here best performing) RSH (CAM-B3LYP-D4) along the RC. Further DFT computational details are presented in Section S1.^87^

## 3 Results and Discussion

### 3.1 Demonstration of the Methodology: a Simple Nucleophilic Substitution

We briefly demonstrate the methodology for a relatively straightforward example of an S_N_2 model reaction, namely for the concerted nucleophilic substitution occurring in the attack of CH_3_Cl by Cl^–^ (Scheme 1).

**Scheme 1:**
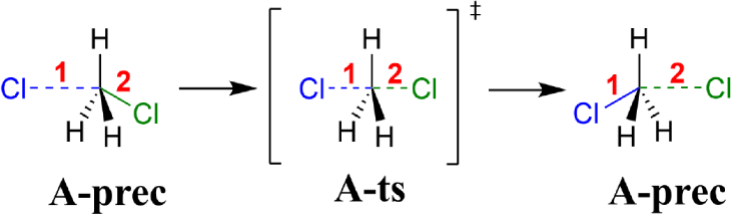
Reaction **A**: Nucleophilic Substitution^a^

a The chlorine of methyl-chloride is exchanged with a free chloride-ion in a concerted nucleophilic substitution. For the analysis along the reaction coordinate (see Figure S9), the difference between the bond order of the formed and cleaved C–Cl bonds (denoted as 1 and 2) is chosen as reaction coordinate.

The underestimation of the barrier height of this reaction by DFT is well-known^88–90^ and it was attributed to SIE and overdelocalization due to the 3*c*/4e nature of the TS.^91^ Accordingly, hybrid and higher rung methods show errors in the range of −9.5 to 1.6 kcal/mol for the barrier height of **A-ts** (Figure S7 and Table S3). Especially for hybrids with lower HFx content [and pure (m)­GGAs], both the sign and size of the density sensitivities correlate well with the energy errors (Figure S8), verifying that *S* ^DFT^ is a suitable measure of the density-driven error for our purposes. The negative sign of the error is also consistent with the expectation of overdelocalization, and thus overstabilization of the TS compared to the reactant state. As expected, hybrids with a large amount of HF exchange, RSHs, and DHs perform better in the [−5, 1.6] kcal/mol error interval. For a more detailed analysis, see Section S2.1.

### 3.2 Self-Interaction and Dispersion Errors: Halocyclization

In the first, synthetically widely applied halocyclization reaction^92–95^ an intramolecular nucleophilic addition is induced by the electrophilic addition of a halogen to the double bond, yielding halogenated cyclic compounds. Here, we follow one of the rare experimental-computational mechanistic studies,^24^ and investigate chlorolactonization of phenyl-pentenoic acid (**B-reac**) catalyzed by a quinuclidine (**qui**) base. The inspected *anti* addition pathway involves a concerted chlorenium transfer, ring closure, and base-assisted substrate deprotonation (see Scheme 2), with a halogen bond formed already in precomplex **B-prec** also playing a role in the selectivity.

**Scheme 2:**
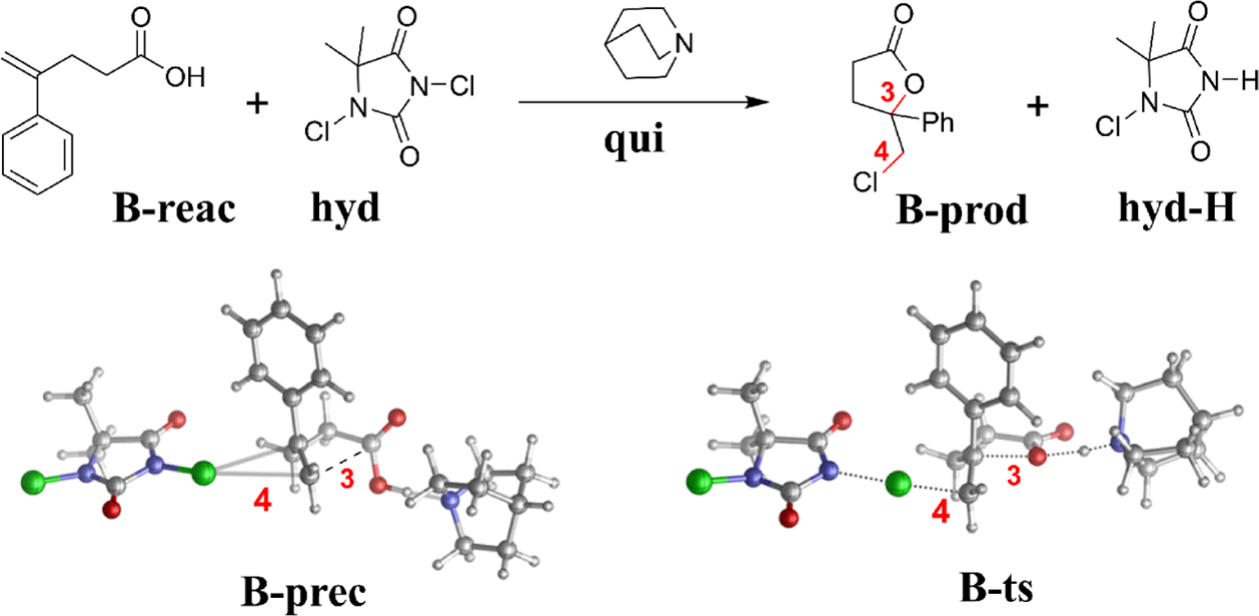
Reaction **B**: Halocyclization[Fn sch2-fn2]

a First the substrate **B-reac**, the N-chlorohydantoin halogen source **hyd** and quinuclidine catalyst **qui** form a ternary complex (**B-prec**). Then, the ring closure, the halogen transfer and the deprotonation proceeds through one concerted transition state (**B-ts**). The average bond order of the forming C–Cl and C–O bonds (denoted by 3 and 4) is chosen as the reaction coordinate.

While the reaction energies are reliable (being mostly consistent within a 1–2 kcal/mol window in Table S4), the **B-ts** barrier heights are analyzed more closely. In Figure 2 we arranged according to Jacob’s ladder the density sensitivities and DFT deviations, the latter ranging from –11 to 7.7 kcal/mol for rungs of hybrids and above.

**Figure 2:**
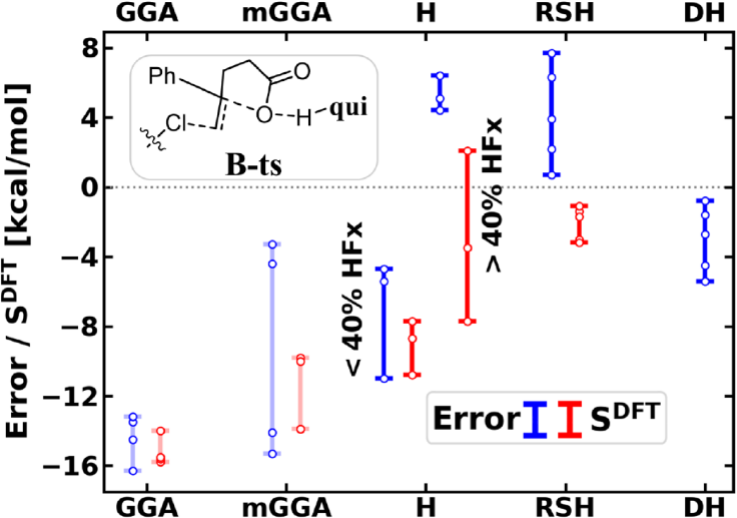
Barrier of the halocyclization reaction (**B-ts** in Scheme 2 with respect to separated reactants): signed error with respect to LNO–CCSD(T) (blue, left bars) and *S* ^DFT^ density sensitivity (red, right bars) of functionals. Results are plotted with bars corresponding to each functional category and white dots represent the individual results. Transparent colors for (m)­GGAs indicate to focus more on the higher rungs. The categorization of the functionals is introduced in Table S2 and the results are collected for each functional in Figure S11 and Table S4.

Considering the hybrids with a low amount of exact exchange [and the (m)­GGAs], **B-ts** and **A-ts** show similar trends, i.e., the negative errors correlate with the density sensitivities (cf. Figures 2 and S8). The density sensitivities increase notably in **B-ts** compared to **B-prec** due to the 3*c*/4e character of the N···Cl···C bond in the TS, which is also an analogy with the S_N_2 model reaction. To our knowledge, these similarities in the electronic structure of the barrier and the corresponding sensitivity to SIE between nucleophilic substitutions (as in **A-ts**) and such electrophilic additions (here, halofunctionalizations) have not been pointed out in the literature.

In accordance with these trends, the hybrids with a larger amount of HFx and the RSHs show moderate negative density sensitivity and less correlation between the energy errors and *S* ^DFT^ and perform better. The positive signed errors appear to be due to the (too) high portion of exact HFx in some of these functionals, which we show by varying their HFx content for this specific TS (as detailed in Section S2.2 and Figure S12).

Regarding dispersion corrections, we consider that their use is (should be) the general practice, so we make only a brief note in the main text. Considering only the energies, GGA methods exhibit smaller errors in the barrier height without the dispersion correction (e.g., BP86-D4: −16.3 kcal/mol, BP86:1.4 kcal/mol, see all data in Table S5). However, inspecting the dispersion and density sensitivity components separately, for (m)­GGAs, the lack of the stabilizing dispersion interaction is compensated by overstabilization due to the overdelocalized density (which is explained in Section S2.2.2 and consistent with similar findings in the literature).^96–98^ While experts would notice these trends, we echo the advice that such compensation of opposite sign effects should not be relied upon. As the need for dispersion corrections is clear, one still needs to decide on the employed model.^45–48^ Due to their dominant role and broad availability in computation chemistry, we employ and compare D3 ^99^ D4 ^100^ and VV10 ^101^ models in Section S2.2.2. While in our cases, the dispersion models are comparable and turn out to be not among the main sources of error, in general, one should be aware of recently found shortcomings and improvements, especially useful for exploration of reaction paths.^102,103^

Regarding the importance of the dispersion component,^45–48^ one can look at the size of the dispersion correction (see Table S2).

Inspecting the DFT errors and the density sensitivities along the RC in the middle and right panels of Figure 3, we find that they vary mostly analogously to the case of the S_N_2 model reaction **A** (Figure S9). One difference is that the density sensitivity values do not tend to zero around the reactant/product complexes (two sides of Figure 3) due to the SIE characteristic of the halogen bonds.^104^ Going from the reactant complex toward **B-ts**, especially for the B3LYP-D4 (orange) curve (and amplified further by BLYP-D4 in Figure S15), we find a concerted increase in the DFT errors and density sensitivities corroborating that density-based SIE is the dominant error. For the methods with higher HFx content (M06-2X-D3 and the RSHs in Figure 3), the self-consistent density is probably considerably better than the ones used for evaluating *S* ^DFT^. Accordingly, their somewhat positive errors along the RC suggest that not only density but also a small amount of functional error could be responsible for their uncertainties.

**Figure 3:**
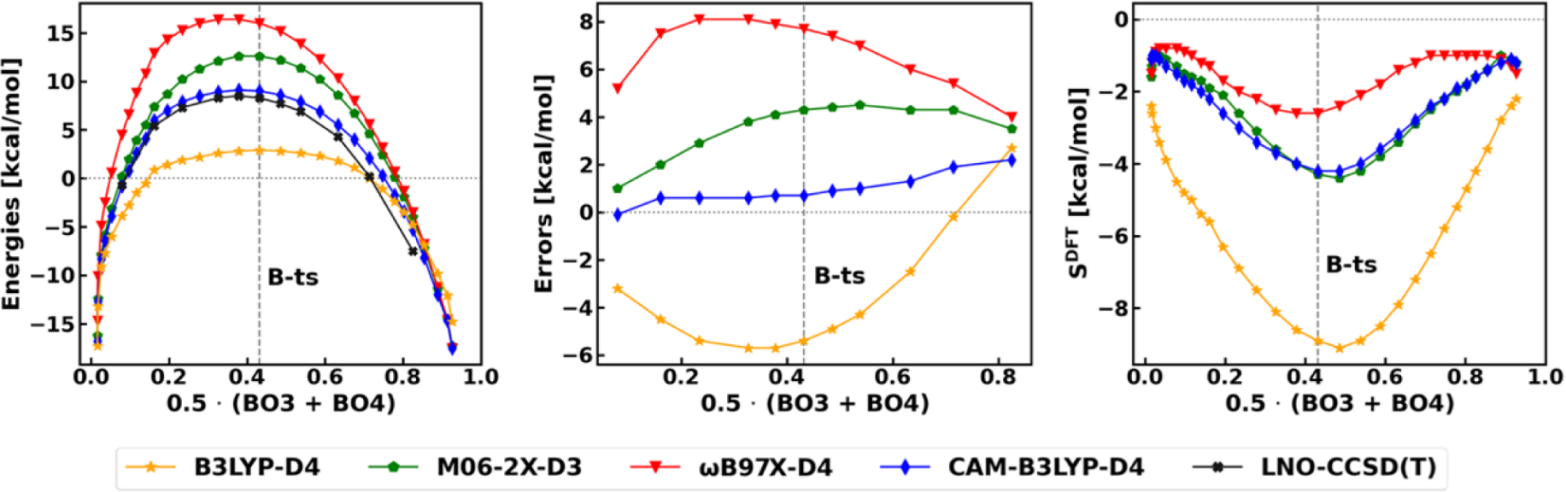
Halocyclization reaction **B**. Left: Electronic energies computed with various methods along the reaction coordinate (RC). The RC is defined as the average Mayer Bond Order^105^ (BO) of the forming bonds (3 and 4 in Scheme 2). The reactant state (CV ≈ 0) corresponds to **B-prec** and the energies are provided with respect to the separated reactants. Middle: signed errors of functionals with respect to LNO–CCSD­(T)/CBS results along the RC. Right: density sensitivities of functionals along the RC. For the BLYP-D4 curves, see Figure S15.

To inspect this more closely, we analyze the correlation energy contributions to the stabilization energies along the RC (Figure S16). The CCSD­(T) correlation energy contribution is much more significant than in the nucleophilic substitution, ranging from −30 to −40 kcal/mol. Moreover, MP2 overestimates the correlation energy contributions by 5–10 kcal/mol, pointing to the significance of higher than second-order correlation. Reproducing such nontrivial electron correlation effects could be challenging even with the advanced functionals, which provides an explanation also for the notable errors even at the DH level (Figure 2 and Table S4).

### 3.3 Multiple Types of SIE: Methylation

Methylations are widely applied transformations in organic and medicinal chemistry,^106–108^ which are often performed using electrophilic methyl sources. The examined reaction is the electrophilic attack of an enolate (**C-en**) by iodomethane (**MeI**) (Scheme 3). This diastereoselective methylation has recently been used as a key step in the total synthesis of stemoamides^109^ and the related computational analysis provided a simple stereoselectivity model.^25^

**Scheme 3:**
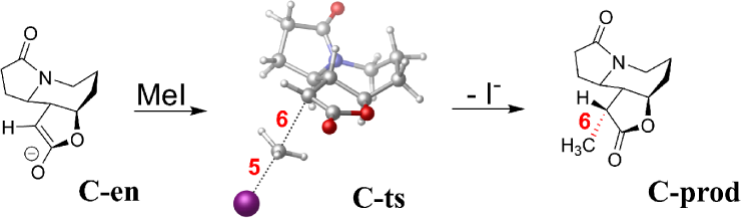
Reaction **C**: Methylation[Fn sch3-fn3]

a Enolate from a trans-fused *γ*-butyrolactone **C-en** is methylated by iodomethane. The difference of the bond orders of the forming C–C and breaking C–I bonds is chosen as the reaction coordinate.

The errors in the barrier height of **C-ts** (Figure S17) and their correlation to the density sensitivities (Figure S18) again points to SIE as the main source of error. However, despite these similarities at first sight, a closer inspection reveals differences. First, the barrier height errors are somewhat lower (−6.5 to +3.8 kcal/mol at the hybrid and above rungs) than in reactions **A-B**. Second, unlike in reactions **A-B**, notable errors also occur not only for the barrier but also in the reaction energy (Figure 4). Third, the reaction energy errors and density sensitivities of Figure 4 are of opposite (positive) sign compared to the barriers of **A-B**, especially for low HFx hybrids (and GGA functionals, see Figure S18) Then, for range-separated hybrids, the density sensitivity becomes close to zero with mostly negative reaction energy errors. In turn, double hybrids generally have positive errors.

**Figure 4:**
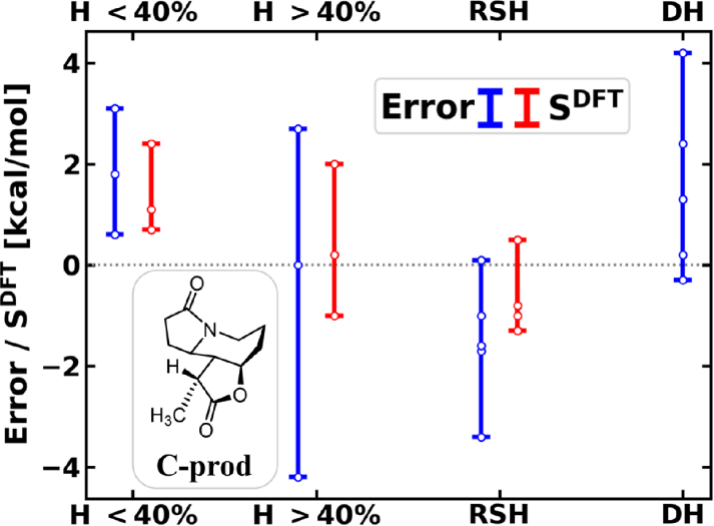
Reaction energy of the methylation (**C-prod** + I^–^ in Scheme 3, with respect to separated reactants): signed error with respect to LNO–CCSD­(T) (left bars, blue) and density sensitivity (right bars, red) of functionals. See (m)­GGA results in Figure S18 and all data in Figure S17 or Table S6.

Since the errors and the density sensitivities correlate in Figure 4, SIE could play a role, but the usual overstabilization by negative density-based errors is not apparently consistent with the positive reaction energy errors. An alternative explanation for the underestimated reaction energy would be an overstabilized reactant state compared to the methylated enolate **C-prod** and an iodide-ion (Scheme 3) in the product state. Namely, enhanced electron delocalization caused by SIE could overstabilize the iodomethane reactant, but cannot be present for the infinitely separated iodide-ion.^110^

Because of this SIE in the reactant, it is helpful to simplify the analysis by removing this source of error and use the product as reference state (Figure S19). Here, the reaction energy of the reverse reaction has a negative error of up to 3 kcal/mol with hybrids (and 7 kcal/mol with GGAs) due to the lack of SIE cancellation between the reactant and product states. Moreover, the error in the reverse barrier height reaches up to −10 kcal/mol with hybrids (and −14 kcal/mol with (m)­GGAs), now matching the size of the SIE in the **A-B** barrier heights. Thus, one should consider two different error sources of opposite signs in reaction **C**: (measured from the reactant state) a negative SIE source due to the 3*c*/4e TS structure and a positive component due to the lack of SIE compensation in the polarizable iodine species.

For the separation and better understanding of these two sources of errors, let us analyze them along the RC (for simplicity, first, with respect to the product state in Figures 5 and S20). The lowest (most negative) density sensitivities are found close to **C-ts** with all functionals. The errors show similar behavior to *S* ^DFT^ in the case of B3LYP-D4, which hybrid is the most sensitive to SIE. In this case, some density sensitivity remains in the precomplex, and even in the reactant state (that is the separated reactants, denoted as **R** in Figure 5), which translates into negative energy errors. Thus, one finds the first SIE type already at the reactant state, which is accompanied by the second SIE source moving toward the TS, and then both tend to diminish at the product state.^111^ Finally, let us note that analogous DFT performance is found for the *syn* methylation here and the *anti* pathway (Figure S24). While such excellent error cancellation is not guaranteed in general, the practically important difference between the *syn* and *anti* barrier heights are reliable (Section S2.3.1) for stereoselectivity conclusions.

**Figure 5:**
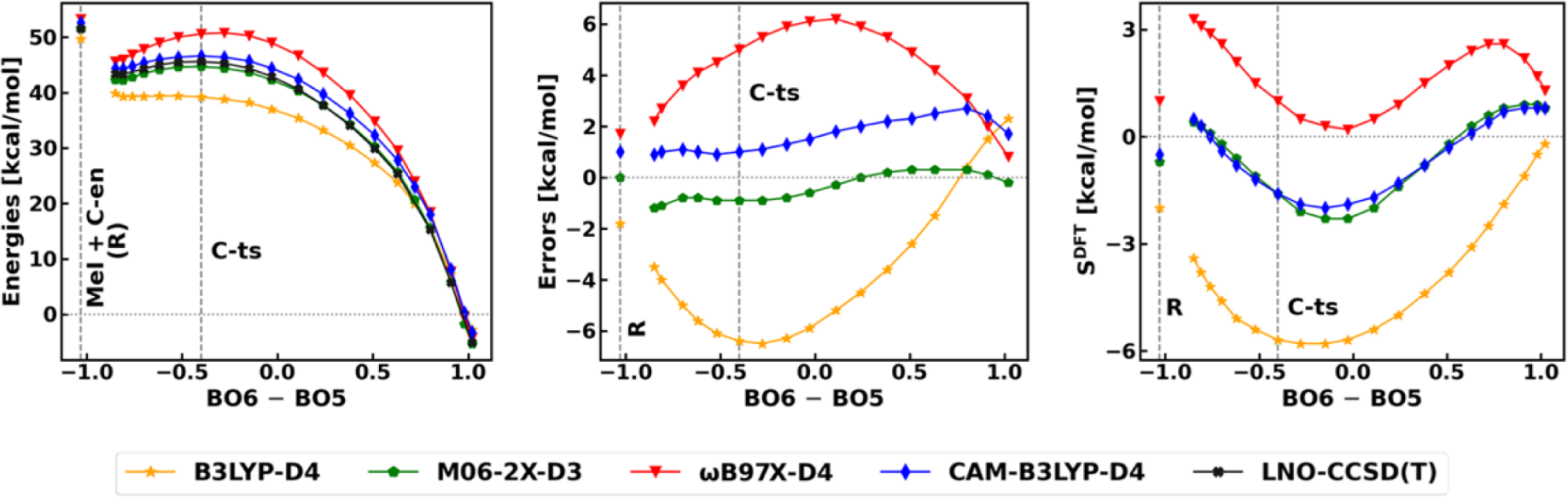
Reverse of the methylation reaction **C**. Left: electronic energies along the RC with respect to the separated products. The RC is defined as the difference between the BOs of the formed and cleaved bonds (6 and 5 in Scheme 3). Middle: signed DFT errors with respect to LNO–CCSD­(T)/CBS along the RC. Right: density sensitivities along the RC. **R** labels the state of infinitely separated reactants. The BLYP-D4 curves are presented in Figure S20.

### 3.4 Complex Interplay of Functional and Density Errors: Michael Addition

Enantioselective Michael addition reactions enable valuable, stereoselective C–C and C–X bond formation.^112–115^ In Scheme 4, we focus on the addition of the nitrostyrol (**ns**) to an enamine species (**D-en**) forming a 6-membered dihydrooxazine N-oxide (**D-oo**) intermediate (via **D-ts**_**1**_). **D-oo** is then rearranged (via **D-ts**_**2**_) into a nitro-substituted cyclobutane (**D-cb**) intermediate, which was found to play a key role in the stereocontrol of organocatalytic Michael additions.^26^

**Scheme 4:**
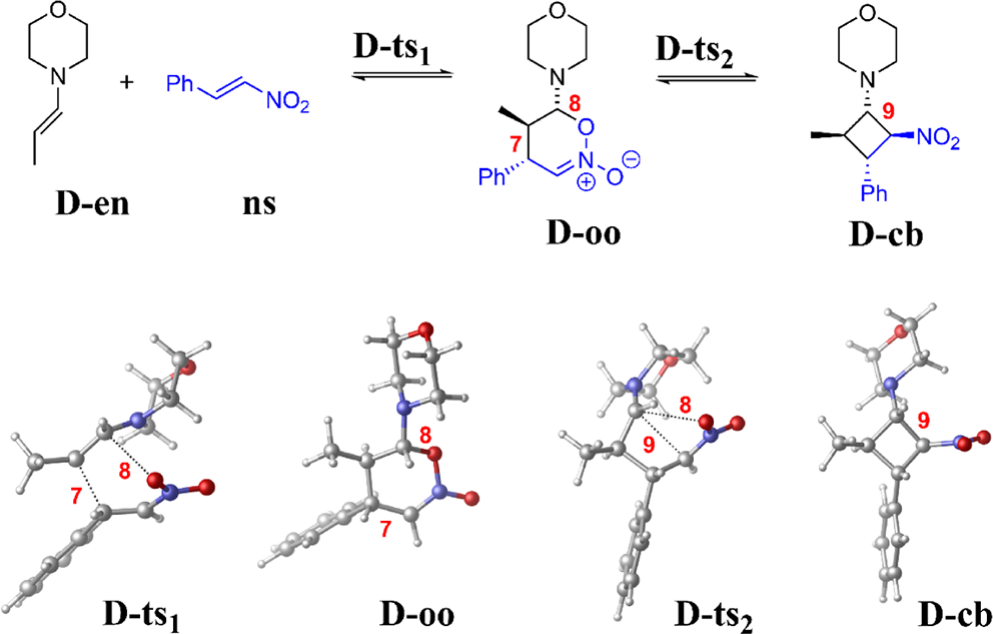
Reaction **D**: Michael Addition[Fn sch4-fn4]

a First, a 6-member ring (dihydrooxoazine-oxide, **D-oo**) is formed, which is rearranged into a 4-member ring (cyclobutane-form, **D-cb**). In the first step, a C–C and a C–O bond is formed, which are denoted by 7 and 8, respectively. In the rearrangement, bond 5 is cleaved and another C–C bond (denoted as 9) is formed instead.

In Figure 6, the energy of the intermediates and transition states are plotted with respect to the separated **D-en** and **ns** obtained with various, at least hybrid rung functionals (bars and dots) and the LNO–CCSD­(T) method (horizontal dashed line). Starting with **D-ts**_**1**_, the at least hybrid DFT results span a 9 kcal/mol range, analogously to the TSs in reactions **A-C**. In contrast, the errors in the **D-oo** intermediate with high rung functionals are −7.5 to 7.1 kcal/mol (larger positive with low HFx, closer to zero with high HFx and notably negative with some RSHs). Next, despite the structural similarity of **D-ts**_**2**_ to **D-ts**_**1**_ and **D-oo**, its errors between −4.9 and +2.5 kcal/mol for hybrids and above are smaller and better centered around LNO–CCSD­(T). Then, in another turn, the errors at the H/RSH/DH rungs in the **D-cb** intermediate are found to be the largest (in the range of [−11.0,8.3] kcal/mol). Note that the intervals of H/RSH/DH errors for **D-oo** and **D-cb** still span 8–10 kcal/mol, even if we take out the positive (popular B3LYP-D4) and negative (SIE resistant RSH LC-ωPBE-D3) outliers, which are also reasonable choices *a priori*.

**Figure 6:**
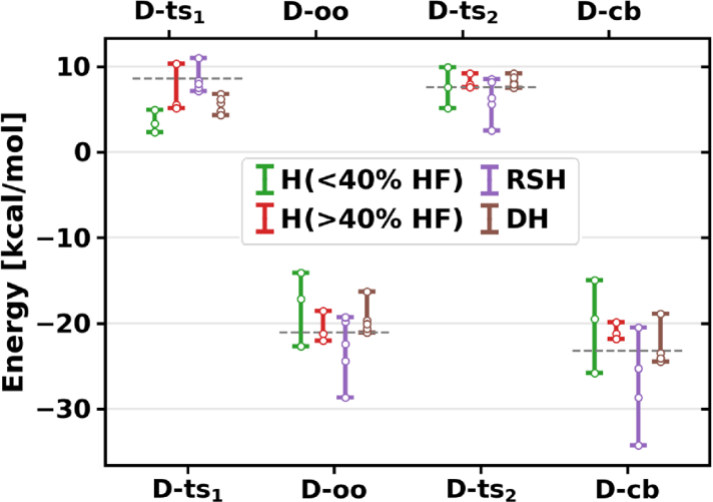
Energies of stationary points in the Michael addition (Scheme 4, with respect to separated reactants) with various methods. DFT results are plotted with bars corresponding to each functional category and dots represent the functionals. The LNO–CCSD­(T) energies are shown with horizontal dashed lines.

The next step in our workflow is the analysis of the potential correlation between DFT errors and density sensitivities (Figure 7 for **D-oo** and Figure S26 for all four species). For **D-ts**_**1**_ we find analogous DFT error and *S* ^DFT^ correlations as for the TSs of reactions **A-C**. Although 3*c*/4e structural elements do not appear to be present, the partly sp^2^-like structure of the carbon pillars in the forming bond 7 may be considered to resemble the case of, e.g., **C-ts**. However, a novel aspect compared to the case of reactions **A-C** is that the **D-oo**, **D-ts**_**2**_, and **D-cb** errors do not correlate with the density sensitivity.^116^ Interestingly, the trends of the *S* ^DFT^ measure for **D-ts**_**2**_ are similar to the case of **D-ts**_**1**_ (Figure S26), but their correlation with the DFT errors is lost. Moreover, the density sensitivities are quite small (mostly in the 1–2 kcal/mol range) for **D-oo** and **D-cb** (Figure S26 and Table S10).

**Figure 7:**
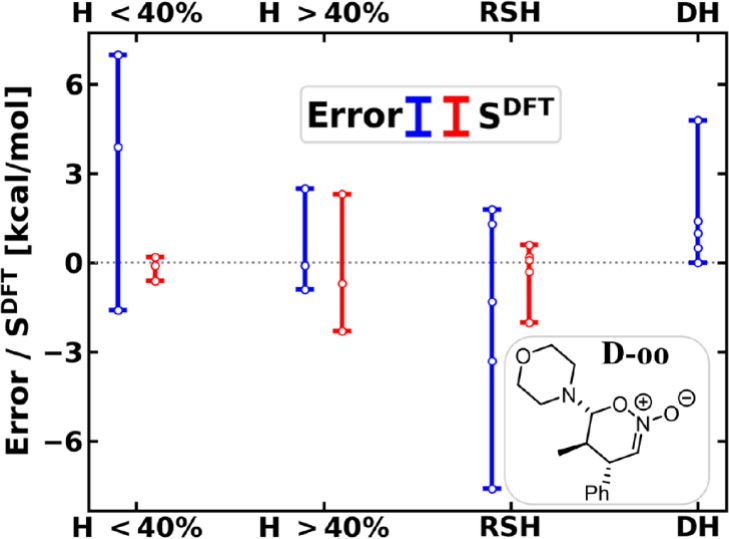
Stability of the **D-oo** with respect to the separated reactants (Scheme 4): signed error with respect to LNO–CCSD­(T) (left bars, blue) and density sensitivity (right bars, red) of functionals. See all data in Figure S27 or Table S10.

To see if multiple error types could explain the trends, we continue with the analysis along the RC. In the first elementary step, a C–C bond and the C–O bond is formed (bonds 7 and 8 in Scheme 4), so a collective variable (CV) averaging their bond orders was chosen as RC.^117^ Along this CV_1_ until **D-ts**_**1**_, similarly to the other reactions, one finds generally negative errors with minima close to the TS (Figure 8). Most *S* ^DFT^ curves on the right panel also show a minimum at (or around) **D-ts**_**1**_. The density sensitivities display another minima around CV_1_ = 0.6 (green highlight in Figure 8), where the first bond is almost completely formed and the second bond is halfway formed (BO8 is close to 0.4). Despite the large negative density sensitivities, the errors are close to zero or rather positive in this green region. Then, moving toward the intermediate **D-oo**, the density sensitivities diminish and the positive errors increase. Combining these observations, especially magnified in the case of B3LYP-D4 (and BLYP-D4 in Figure S30) one finds a positive error emerging and growing from CV_1_ = 0.3 to CV_1_ = 0.6, partly canceling the negative density-driven error. Then, *S* ^DFT^ steeply decreases above CV_1_ = 0.6 toward **D-oo**, which correlates well with the increase of the total DFT error. This suggests a positive functional error which is no longer canceled by the density-based error around **D-oo**.

**Figure 8:**
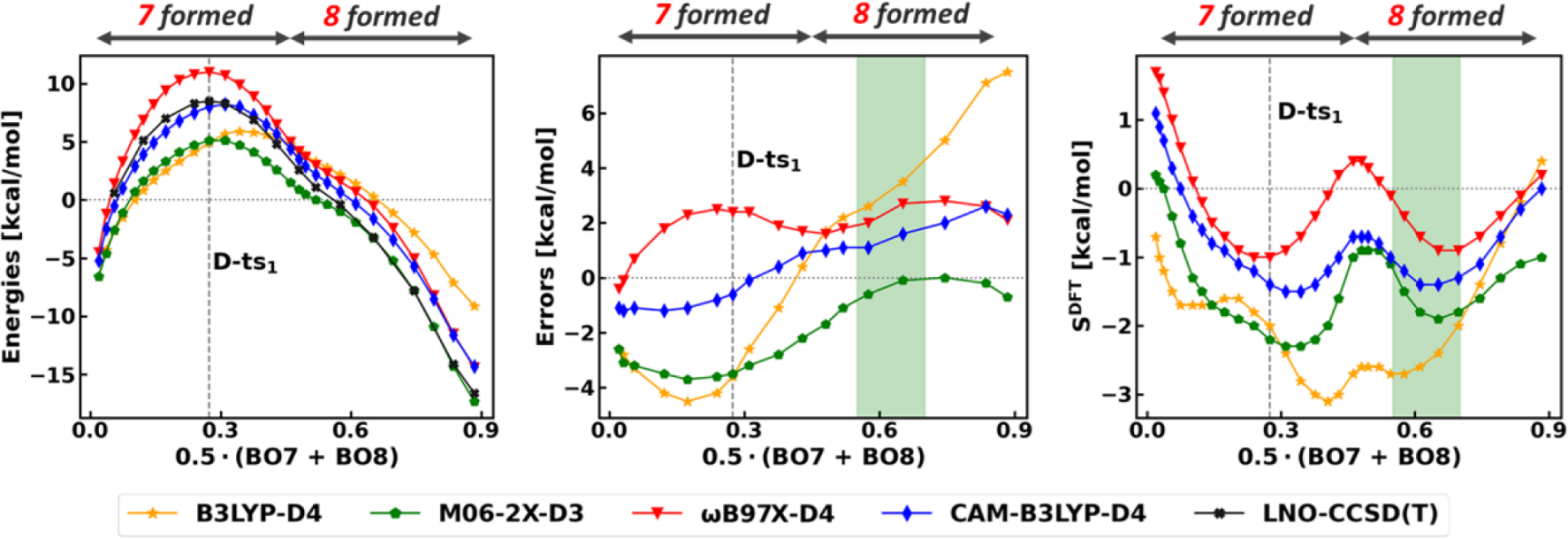
First step of the Michael addition. Left: electronic energies along the RC with respect to the separated reactants. The RC is defined as the average BO of the forming bonds (7 and 8 in Scheme 4). Middle: signed errors of functionals with respect to LNO–CCSD­(T)/CBS results along the RC. Right: density sensitivities of functionals along the RC. The structure representative of the green highlighted region is depicted in Figure S28. For the BLYP-D4 curves, see Figure S30.

For the second elementary step from **D-oo** through **D-ts**_**2**_, the CV_2_ of BO9–BO8, that is the difference between the BOs of the cleaved C–O bond 8 and the forming C–C bond 9 (Scheme 4) is a reasonable RC. The density sensitivities along this CV_2_ in Figure 9 are generally negative and are the most negative approximately where these bonds are halfway formed or cleaved (green regions around CV_2_ = −0.3 and CV_2_ = 0.4). The errors and density sensitivities around **D-cb** (right sides of Figure 9 panels) display an analogous picture to **D-oo** (left sides of Figure 9 panels): as the *S* ^DFT^ curves approach zero, the DFT errors become more positive.

**Figure 9:**
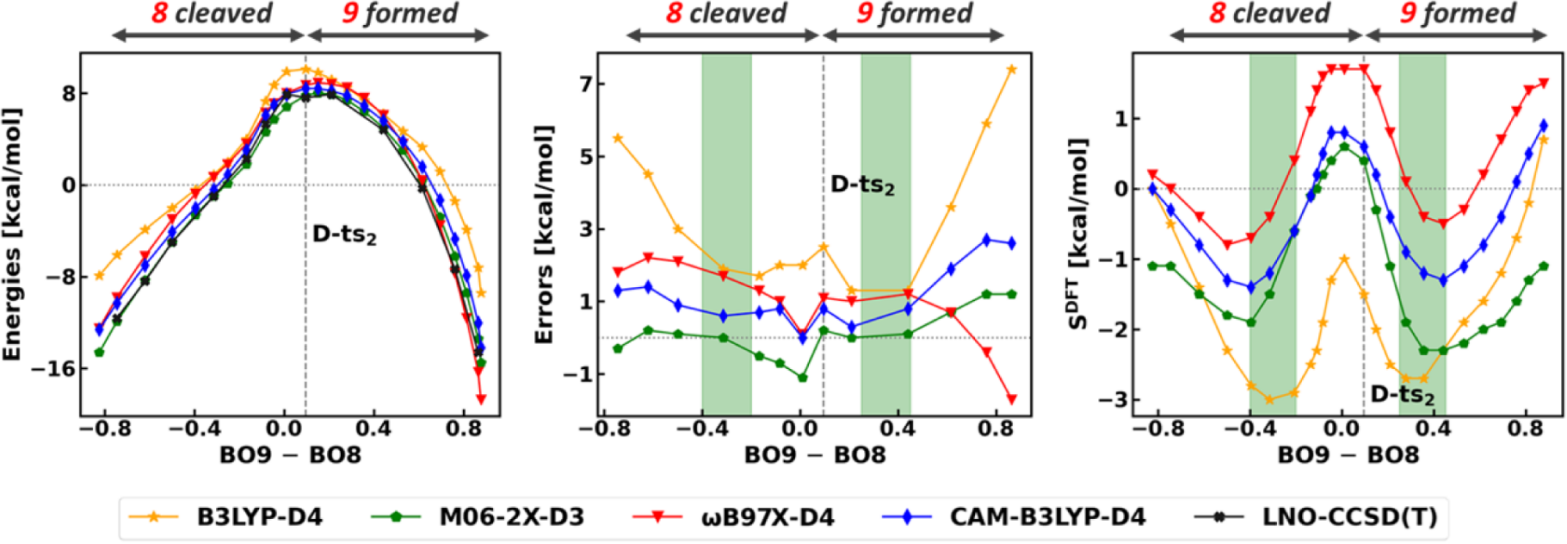
Second step of the Michael addition. Left: electronic energies along the RC with respect to the separated reactants. The RC is defined as the difference between the BOs of the formed and cleaved bonds (9 and 8 in Scheme 4). Middle: signed errors of functionals with respect to LNO–CCSD­(T)/CBS results along the RC. Right: density sensitivities of functionals along the RC. The structures representative of the green highlighted regions are depicted in Figure S29. For the BLYP-D4 curves, see Figure S31.

The analysis along the RC revealed that *S* ^DFT^ is most negative where one of the bonds is about halfway formed or cleaved. These points include one of the transition states (**D-ts**_**1**_) and the three green regions in Figures 8 and 9. However, in contrast to the previously discussed reactions, in these points except for **D-ts**_**1**_, the large negative density sensitivities do not result in negative errors but the errors are close to zero. This suggests a large positive functional error along the reaction coordinate, that starts to appear around **D-ts**_**1**_, becomes large from **D-oo** through **D-ts**_**2**_ to **D-cb**, and is partly compensated by the negative density error where the SIE is large.

In light of this, we can inspect closer the most confusing case of **D-ts**_**2**_. Interestingly, **D-ts**_**2**_ is between the two minima on the *S* ^DFT^ curves of Figure 9, as it has a fully cleaved C–O (8) bond but a not yet started C–C (9) bond (BO8 = 0.00, BO9 = 0.09). Between these bond breaking and formation steps, the *S* ^DFT^ curves have local maxima around **D-ts**_**2**_, which affect the DFT error curves around **D-ts**_**2**_. Namely, corresponding little peaks appear also on the DFT error curves around **D-ts**_**2**_, where the errors originating from functional and density sources cancel differently than in the neighboring green regions.

Considering the potential source of functional error components (detailed in Figure S33 and its discussion in the SI), the MP2 and post-MP2 components are both sizable but they are relatively constant with shallow local minima around the bond-breaking/formation halfway points. Compared to that the size and shape of the HF contribution correlate well with the functional error starting from **D-ts**_**1**_ and for **D-oo** and **D-cb** too, suggesting an imbalance of the exchange and correlation functional components along this CV interval. All in all, a positive functional error for the relatively similar **D-oo**, **D-ts**_**2**_, and **D-cb** structures in combination with the uncovered complex density sensitivity behavior along the CV explains the strange error pattern for all four structures in Figure 6.

### 3.5 General Procedure to Guide Functional Choice

In this section, we combine the case by case experience above to draw more general conclusions and suggest a practically applicable workflow for DFT model selection. The overall performance of some of the advanced, popular and best-performing methods is illustrated in Table 1. Although none of the highlighted methods are ideal for all **A-D** examples, some RSH and DH methods (especially the more recent DHs, e.g., revDSD-PBEP86-D4) are reliable for multiple reactions and one finds at least one or more suitable functionals for each reaction.

**Table 1:** Energy Errors with Respect to LNO-CCSD­(T) [in kcal/mol] for the Best-Performing Functionals in the Low HFx, High HFx, RS, and D Hybrid Categories (Data for All Studied Functionals is in Table S12)

Method	**A-ts**	**B-ts**	**C-ts**	**D-ts**_**1**_	**D-cb**
B3LYP-D4 (20%)	–7.7	–5.4	–4.6	–3.7	8.2
M06-2X-D3 (54%)	–3.2	4.4	–0.9	–3.5	1.4
ωB97X-D4	–1.3	7.7	3.3	2.4	–2.1
ωB97M-V	–3.7	2.2	0.8	–1.5	–1.3
CAM-B3LYP-D4	–4.0	0.7	–0.1	–0.6	2.7
revDSDPBEP86-D4	3.5	1.6	1.2	1.8	1.4

For a broader perspective, we arranged all DFT errors according to Jacob’s ladder in Table S12. In accord with the expectations, there is a general improvement from GGA toward DH with some are exceptions. However, for all studied reactions none of the rungs or categories perform consistently the best. If we consider only the best performers of the rungs/categories (Table S13), there is clear, systematic improvement toward the higher rungs. For the best functionals at the RSH/DH rungs, there are 2–3 energy differences where the errors are within or close to as well as still above chemical accuracy. Therefore, at least for such complicated cases, a more careful analysis is useful going beyond the standard approach of looking at error statistics against benchmark results.

To make the suggested analysis more practical, we note that probably not all steps are necessary for most reactions and thus we arranged the steps into a workflow funnel (Figure 10).

**Figure 10:**
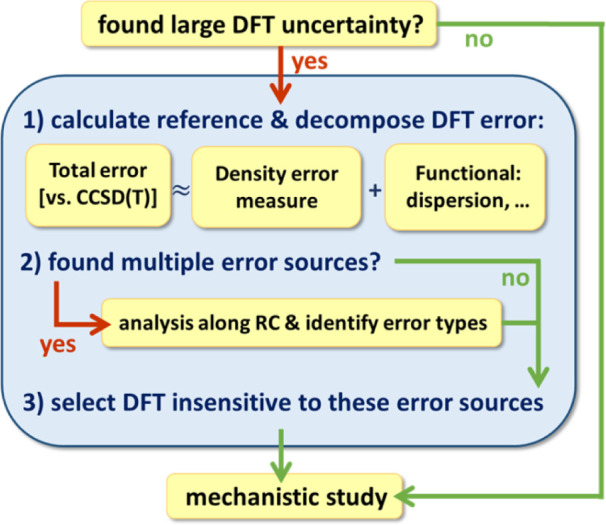
Overview of the suggested computational tools and analysis workflow.

To start, the knowledge of at least some key structures or a preliminary reaction mechanism is needed to quickly test multiple DFT methods against each other. For this consistency check one can recommend advanced, generally well-performing and somewhat diverse functionals. In practice, it is not necessary to test as many entries as we did here. For example, a few low and high HFx containing, as well as RS and double hybrids can be assessed selected from general^27–30^ or system-specific statistical studies, from Table S2, or even the short list of Table 1 could suffice (as it is similar to the outcome in refs. 27–30 ). As also noted before in the expert community, a common pitfall at this stage is to test only reaction energies or intermediates (due to, for example, easier access to experimental data for these). However, transition states and other mechanistically relevant structures along the (preliminary) reaction coordinate should not be overlooked, e.g., because of their higher sensitivity to DFT errors (cf. reactions **A-C**).

If satisfactory consistency is found for a representative set of species, as often the case in organic chemistry, one can clearly proceed with the mechanistic study. If too large disagreements are obtained, the next step is analysis according to DFT rungs as well as accurate (local correlation based) CCSD­(T)^35^ reference computations (including checks if single-reference CC methods are applicable.^34^

As CCSD­(T) based references become more affordable and popular, here, one should point out the importance of reaching proper level of convergence both in the basis set and the local approximations (see Section S1).^35^

At this stage, one may be able to identify a satisfactory functional that exhibits sufficiently low errors for all tested structures, especially if there is only one source of DFT error. Alternatively, continuing the analysis of DFT performance can be valuable under the following circumstances:

i if DFT disagreements exceed the acceptable accuracy target,
ii if there are indications of multiple error sources, e.g., suggested by complex error patterns, or
iii if one seeks to avoid coincidental error compensations and ensure that the model provides accurate results for sound reasons across the entire mechanistic study.

A concern related to points (i)–(iii) is that access to all relevant reaction pathways and structures at this initial phase may be limited, as mechanistic studies often involve a variety of reactants, catalysts, isomers, conformers, solvents/environments, reaction paths, and so on. Thus, choosing a model that is robust and appropriate according to a larger number of and more diverse measures increases the likelihood that its strong performance will extend to a broader chemical space of interest. Toward that end, one can proceed by decomposing the total DFT error into functional and density components. This step is made simple and easily accessible here by combining the LNO–CCSD(T) reference and exploring measures for the dispersion and density sensitivity components.

If the error decomposition yields a clear, dominant source of error at this point, such as in reaction **A**, one may conclude the analysis by choosing a functional (group) that is designed to be more resilient against such errors (e.g., 3 out of 5 RSHs performs well for reaction **A**).

If multiple significant error sources are found, such as in reactions **B-D**, we find it useful to extend this analysis to a broader set of structures, e.g., along (a preliminary) reaction coordinate via step 2) of the workflow (Figure 10). In our examples, data points are taken relatively densely for demonstrative purposes, as 20–30 LNO–CCSD­(T) computations per elementary step were easily affordable. In practice, a half as or even more sparse exploration along the reaction coordinate should often be sufficient. The key idea is to find structure(s) along the reaction coordinate, where the behavior of the DFT errors is simpler, ideally with only one dominant error source. While the dispersion corrections turned out to be suffienct for our specific reactions, in genaral, caution can be still advised for studies along the RC for cases similar to problematic ones reported recently.^102,103^ For the halocyclization and even more so for the methylation (reactions **B** and **C**), this point turned out to be the product state, where only one of the two types of SIE is dominant. While for the Michael addition (reaction **D**) the intermediates are the best option to decouple the functional error from the complex density sensitivity pattern along the RC.

Next, one can retain a subset of functionals working well for the so-identified structure(s) with one dominant error source and follow the changes in the different error types from these point(s). Then, one can use this understanding to explain the potential error amplification or cancellation occurring at the structures with multiple error sources. The main benefit is that we can narrow the selection among the best-performer models in terms of energies by setting aside methods with “false positive” matches, i.e., ones with seemingly good results due to error cancellation.

For example, we can eliminate one source of error by finding points where, e.g., the density sensitivity diminishes (as it has a direct measure). By decoupling the error sources, e.g., for reaction **B**, we could explain that the compensation of dispersion and density errors is behind the small (even 0.3–1.4 kcal/mol) error of some (m)­GGAs (Table S5). The cancellation of two SIE types is found to be responsible for the consistently small (0.8–1.0 kcal/mol) error of all hybrids with high HFx content for reaction **C** (Table S6). For reaction **D**, our approach catches functionals that are good for the intermediates and spot on for **D-ts**_**2**_ (with 0.0–0.3 kcal/mol error, e.g., for TPSSh-D4 or MN15-D3) because of benefiting from error cancellation (Figure S27).

The most rigorous selection criteria for the functionals are to be resilient against all separated sources of errors and to maintain a consistent performance for an extended set of structures, e.g., along a RC. For example, some but not all RS (e.g., CAM-B3LYP-D4) and double hybrids were found the least sensitive to the dominant SIE in reactions **B** and **C**, and some high HFx containing hybrids (especially M06-2X-D3) are also among the best performers for **C**. However, considering reaction **D**, the errors of these (e.g., CAM-B3LYP-D4, M06-2X-D3) functionals vary more along the RC and thus do not remain the best choices. In turn, the potential energy parallelity of, e.g., ωB97M-V with LNO-CCSD­(T) is outstanding for reaction **D** (Table S10), while it is relatively good but not the best performer for reactions **A–C**. All in all, the proposed workflow led us to at least one reliable functional for each reaction, enabling one to proceed with a more exhaustive mechanistic study.

The calculations in all steps of this workflow can be carried out routinely with both openly (for academics) and commercially accessible programs^69–71,77,118^ (see sample input files in Section S4). The computational cost of the LNO–CCSD­(T) reference energies is similar to that of the structure optimization and harmonic frequencies with a hybrid DFT and required here at most 25 GB memory. Thus, hundreds of LNO–CCSD­(T) computations were easily possible utilizing at most half a day each on 8–16 cores, even for the largest species in this study. As reviewed in detail in ref. 35 well converged LNO–CCSD­(T)/CBS references can be obtained nowadays with relatively simply accessible resources for 100–200 atoms, while up to 1000-atom computations were also reported,^39^ well beyond the size of what is needed for DFT benchmarking. The density sensitivity calculations cost just as much as a single point hybrid DFT energy evaluation and are even more broadly accessible.

## 4 Conclusions

Our study was initiated by unforeseen DFT uncertainties (cf. Figure 1) in our computational research exploring the thermochemistry and kinetics of organic reactions and their mechanisms. With the aim to find predictive functionals for these studies, we developed an approach enables the separation, identification, and thus a more detailed understanding of the underlying causes. To that end, we combined well-converged (LNO-based local) CCSD­(T) references^35^ and the decomposition of the corresponding DFT deviations into terms approximating functional (dispersion, correlation, exchange, etc.) and density-driven components.^40^ If multiple issues were detected, we followed the trends along the reaction coordinate, which helped to characterize different error types and disentangle their potentially complicated interplay. For example, we successfully distinguished three kinds of self-interaction-driven density errors of different origins from other (functional) sources.

Even when we focused on advanced, hybrid and higher rung functionals, took out otherwise reasonable outliers and considered some of the most popular functionals, 8–13 kcal/mol DFT disagreement remained in these case studies. Such large DFT uncertainties go against current majority expectations about the performance of modern functionals on organic reactions. However, one can also point out that rare cases may be down-weighted in statistics on large data sets, DFT benchmarks are concentrated on molecules below 25-atoms^27–30^ and valuable studies on more practical reactions are scarce.^119–123^ The proposed method offers to go beyond broad statistics based expectations by enabling a systematic understanding of the system specific sources of DFT uncertainties. The so uncovered, clearly targetable examples in main group chemistry and the detailed error characterization should also motivate and contribute to the future advancement of DFT models addressing the underlying causes.

Moreover, bringing the above analysis tools from the domain of simple, textbook systems to real-life catalytic reactions already revealed some lessons beyond the scope of the studied reactions. For example, refining the current expectation, larger self-interaction error (SIE) based issues in the density occurred close to bond-breaking and forming regions. These SIE-sensitive regions often but not always coincide with transition state (TS) structures, relevant e.g., for reaction **D** or more generally also for barrierless processes. In addition to the common case of SIE in nucleophilic substitution TSs, we could also understand their analogy with the seemingly unrelated electrophilic attack of unsaturated bonds (reactions **B** and **C**). The reported investigations are also connected to potential modeling pitfalls that are noted before in the literature but still worth reiterating. Namely, higher uncertainties can occur not only for TSs, but e.g., for intermediates and other relevant structures. Moreover, multiple factors can mislead the DFT model selection, such as focusing only on equilibrium structures (due to simpler access to experimental data) or where multiple DFT error types could cancel and not including the more complicated ones.

If motivated to look at them, expert practitioners would notice the signs prompting our study (disagreement of broadly trusted functionals with each other and/or benchmarks, large dispersion correction, maybe even sensitivity in the density via deviations between GGAs and hybrids, etc.). The advancement here are approaches for the next steps to understand and mitigate these issues. A significant benefit is the ability to explain counterintuitive DFT results in real-life, complex processes emerging from multiple error sources that can amplify or cancel differently along the reaction coordinate. The model selected on the basis of the gained understanding then can be expected to perform better for the entire computational study also outside of the limited number of initially tested structures. The proposed tools are ready for practical use as they are simple, (openly) accessible in multiple codes,^69–71^ and fast enough to fit into routine, DFT-based thermochemistry protocols.

While the four reaction types examined here (nucleophilic substitution, halocyclization,^24^ methylation,^25^ and Michael addition^26^) have broad synthetic relevance by themselves, the underlying issues originate in motifs frequently occurring across chemistry. These include molecular interactions and bond transformations around polarizable (an)­ionic and π-systems, σ-hole interactions, and three-center four-electron (TS) structures. Our focus here was main group chemistry, but the proposed tools are readily applicable in other fields,^35^ such as (single reference) transition metal, surface, or biochemistry. For all investigated reactions, it was possible to characterize multiple sources of errors, understand their interplay and find at least one (a few) suitable functional countering the causes. Thus, such robust model selection approaches should help to make these ever more automated computations more predictive.

## Supplementary Material







## Data Availability

The raw energy data obtained from the computations is provided in Excel format, and all molecular structures are collected into a zip-compressed folder.

## Notes

Computational details; additional figures, tables, and analysis; numerical data presented in the figures of the main text; as well as sample inputs for calculations with Mrcc are provided in the Supporting Information (PDF)
Structure of the studied molecules (ZIP)
All employed DFT and LNO-CCSD­(T) energies (XLSX)

## References

[1] Tantillo D. J. (2016). Faster,
Catalyst! React! React! Exploiting Computational Chemistry for Catalyst
Development and Design. Acc. Chem. Res.

[2] Ess D., Gagliardi L., Hammes-Schiffer S. (2019). Introduction: Computational Design
of Catalysts from Molecules to Materials. Chem.
Rev.

[3] Ahn S., Hong M., Sundararajan M., Ess D. H., Baik M.-H. (2019). Design
and Optimization of Catalysts Based on Mechanistic Insights Derived
from Quantum Chemical Reaction Modeling. Chem.
Rev.

[4] Melnyk N., Iribarren I., Mates-Torres E., Trujillo C. (2022). Theoretical Perspectives
in Organocatalysis. Chem. - Eur. J..

[5] Williams W. L., Zeng L., Gensch T., Sigman M. S., Doyle A. G., Anslyn E. V. (2021). The evolution of data-driven modeling
in organic chemistry. ACS Cent. Sci.

[6] Kalikadien A. V., Mirza A., Hossaini A. N., Sreenithya A., Pidko E. A. (2024). Paving the road towards automated
homogeneous catalyst
design. ChemPluschem.

[7] Reid J. P., Sigman M. S. (2018). Comparing quantitative prediction
methods for the discovery
of small-molecule chiral catalysts. Nat. Rev.
Chem.

[8] Zahrt A. F., Athavale S. V., Denmark S. E. (2020). Quantitative
Structure-Selectivity
Relationships in Enantioselective Catalysis: Past, Present, and Future. Chem. Rev.

[9] Durand D. J., Fey N. (2019). Computational Ligand Descriptors
for Catalyst Design. Chem. Rev.

[10] Soyemi A., Szilvási T. (2021). Trends in
computational molecular catalyst design. Dalton
Trans.

[11] Grimme S., Schreiner P. R. (2018). Computational
Chemistry: The Fate of Current Methods
and Future Challenges. Angew. Chem., Int. Ed.

[12] Harvey J. N., Himo F., Maseras F., Perrin L. (2019). Scope and Challenge
of Computational Methods for Studying Mechanism and Reactivity in
Homogeneous Catalysis. ACS Catal.

[13] Funes-Ardoiz I., Schoenebeck F. (2020). Established
and Emerging Computational Tools to Study
Homogeneous Catalysis-From Quantum Mechanics to Machine Learning. Chem.

[14] Bachrach S. M. (2014). Challenges
in computational organic chemistry. Wiley Interdiscip.
Rev.: Comput. Mol. Sci.

[15] Mata R. A., Suhm M. A. (2017). Benchmarking Quantum Chemical Methods: Are We Heading
in the Right Direction?. Angew. Chem., Int.
Ed.

[16] Houk K. N., Liu F. (2017). Holy Grails for Computational Organic Chemistry and Biochemistry. Acc. Chem. Res.

[17] Ryu H., Park J., Kim H. K., Park J. Y., Kim S.-T., Baik M.-H. (2018). Pitfalls in Computational Modeling of Chemical Reactions
and How To Avoid Them. Organometallics.

[18] Plata R. E., Singleton D. A. (2015). A Case
Study of the Mechanism of Alcohol-Mediated Morita
Baylis-Hillman Reactions. The Importance of Experimental Observations. J. Am. Chem. Soc.

[19] Sperger T., Sanhueza I. A., Schoenebeck F. (2016). Computation and experiment: a powerful
combination to understand and predict reactivities. Acc. Chem. Res.

[20] Bursch M., Mewes J.-M., Hansen A., Grimme S. (2022). Best-Practice
DFT Protocols
for Basic Molecular Computational Chemistry. Angew. Chem., Int. Ed.

[21] Fey N., Lynam J. M. (2022). Computational mechanistic study in organometallic catalysis:
Why prediction is still a challenge. Wiley Interdiscip.
Rev.: Comput. Mol. Sci.

[22] Burrows C. J. (2017). Holy Grails
in Chemistry, Part II. Acc. Chem. Res.

[23] Goerigk L., Casanova-Páez M. (2020). The Trip to
the Density Functional Theory Zoo Continues:
Making a Case for Time-Dependent Double Hybrids for Excited-State
Problems. Aust. J. Chem.

[24] Yousefi R., Sarkar A., Ashtekar K. D., Whitehead D. C., Kakeshpour T., Holmes D., Reed P., Jackson J. E., Borhan B. (2020). Mechanistic Insights into the Origin of Stereoselectivity
in an Asymmetric Chlorolactonization Catalyzed by (DHQD)_2_PHAL. J. Am. Chem. Soc.

[25] Csókás D., Siitonen J. H., Pihko P. M., Pápai I. (2020). Conformationally
Locked Pyramidality Explains the Diastereoselectivity in the Methylation
of trans-Fused Butyrolactones. Org. Lett.

[26] Földes T., Madarász Á., Révész Á., Dobi Z., Varga S., Hamza A., Nagy P. R., Pihko P. M., Pápai I. (2017). Stereocontrol
in Diphenylprolinol
Silyl Ether Catalyzed Michael Additions: Steric Shielding or Curtin–Hammett
Scenario?. J. Am. Chem. Soc.

[27] Goerigk L., Hansen A., Bauer C., Ehrlich S., Najibi A., Grimme S. (2017). A look at the density functional theory zoo with the
advanced GMTKN55 database for general main group thermochemistry,
kinetics and noncovalent interactions. Phys.
Chem. Chem. Phys.

[28] Mardirossian N., Head-Gordon M. (2017). Thirty years
of density functional theory in computational
chemistry: an overview and extensive assessment of 200 density functionals. Mol. Phys.

[29] Peverati R., Truhlar D. G. (2014). Quest for a universal
density functional: The accuracy
of density functionals across a broad spectrum of databases in chemistry
and physics. Philos. Trans. R. Soc., A.

[30] Santra G., Calinsky R., Martin J. M. L. (2022). Benefits
of Range-Separated Hybrid
and Double-Hybrid Functionals for a Large and Diverse Data Set of
Reaction Energies and Barrier Heights. J. Phys.
Chem. A.

[31] Morgante P., Peverati R. (2020). The devil in the details:
A tutorial review on some
undervalued aspects of density functional theory calculations. Int. J. Quantum Chem.

[32] Raghavachari K., Trucks G. W., Pople J. A., Head-Gordon M. (1989). A fifth-order
perturbation comparison of electron correlation theories. Chem. Phys. Lett.

[33] Bartlett R. J., Musiał M. (2007). Coupled-cluster theory in quantum chemistry. Rev. Mod. Phys.

[34] The employed CCSD(T) model assumes an electronic structure dominated by single-reference character. The (low energy) reaction pathes between single-reference molecules are typically well described with CCSD(T) and the applicability of unrestricted (LNO-)CCSD(T)^124^ goes even further to the domain of mildly multideterminantal cases. Moreover, HF and CC convergence, as well as CC wave function based (T1, T2, D1···) indicators usually reliably show when the limits of CCSD(T) is approached. Then, if affordable, we recommend to look at multireference based indicators obtained e.g. from CASSCF wave functions.

[35] Nagy P. R. (2024). State-of-the-art
local correlation methods enable accurate and affordable gold standard
quantum chemistry up to a few hundred atoms. Chem. Sci.

[36] Li H., Mansoori Kermani M., Ottochian A., Crescenzi O., Janesko B. G., Truhlar D. G., Scalmani G., Frisch M. J., Ciofini I., Adamo C. (2024). Modeling Multi-Step
Organic Reactions:
Can Density Functional Theory Deliver Misleading Chemistry?. J. Am. Chem. Soc.

[37] Nagy P. R., Kállay M. (2017). Optimization of the linear-scaling local natural orbital
CCSD­(T) method: Redundancy-free triples correction using Laplace transform. J. Chem. Phys.

[38] Nagy P. R., Samu G., Kállay M. (2018). Optimization
of the linear-scaling
local natural orbital CCSD­(T) method: Improved algorithm and benchmark
applications. J. Chem. Theory Comput.

[39] Nagy P. R., Kállay M. (2019). Approaching
the basis set limit of CCSD­(T) energies
for large molecules with local natural orbital coupled-cluster methods. J. Chem. Theory Comput.

[40] Sim E., Song S., Vučković S., Burke K. (2022). Improving
Results by Improving Densities: Density-Corrected Density Functional
Theory. J. Am. Chem. Soc.

[41] Song S., Vučković S., Sim E., Burke K. (2022). Density-Corrected
DFT Explained: Questions and Answers. J. Chem.
Theory Comput.

[42] Teale A. M., Helgaker T., Savin A., Adamo C., Aradi B., Arbuznikov A. V., Ayers P. W., Baerends E. J., Barone V., Calaminici P., Cancès E., Carter E. A., Chattaraj P. K., Chermette H., Ciofini I., Crawford T. D., De Proft F., Dobson J. F., Draxl C., Frauenheim T., Fromager E., Fuentealba P., Gagliardi L., Galli G., Gao J., Geerlings P., Gidopoulos N., Gill P. M. W., Gori-Giorgi P., Görling A., Gould T., Grimme S., Gritsenko O., Jensen H. J. A., Johnson E. R., Jones R. O., Kaupp M., Köster A. M., Kronik L., Krylov A. I., Kvaal S., Laestadius A., Levy M., Lewin M., Liu S., Loos P.-F., Maitra N. T., Neese F., Perdew J. P., Pernal K., Pernot P., Piecuch P., Rebolini E., Reining L., Romaniello P., Ruzsinszky A., Salahub D. R., Scheffler M., Schwerdtfeger P., Staroverov V. N., Sun J., Tellgren E., Tozer D. J., Trickey S. B., Ullrich C. A., Vela A., Vignale G., Wesolowski T. A., Xu X., Yang W. (2022). DFT exchange: sharing
perspectives on the workhorse of quantum chemistry and materials science. Phys. Chem. Chem. Phys.

[43] Becke A. D. (2014). Perspective:
Fifty years of density-functional theory in chemical physics. J. Chem. Phys.

[44] Cohen A. J., Mori-Sánchez P., Yang W. (2012). Challenges for Density Functional
Theory. Chem. Rev.

[45] Grimme S., Hansen A., Brandenburg J. G., Bannwarth C. (2016). Dispersion-Corrected
Mean-Field Electronic Structure Methods. Chem.
Rev.

[46] Hermann J., DiStasio R. A. J., Tkatchenko A. (2017). First-Principles
Models for van der
Waals Interactions in Molecules and Materials: Concepts, Theory, and
Applications. Chem. Rev.

[47] Klimeš J., Michaelides A. (2012). Perspective: Advances and challenges
in treating van
der Waals dispersion forces in density functional theory. J. Chem. Phys.

[48] Becke A. D., Johnson E. R. (2007). Exchange-hole dipole
moment and the dispersion interaction
revisited. J. Chem. Phys.

[49] Janesko B. G. (2021). Replacing
hybrid density functional theory: motivation and recent advances. Chem. Soc. Rev.

[50] Bryenton K. R., Adeleke A. A., Dale S. G., Johnson E. R. (2023). Delocalization error:
The greatest outstanding challenge in density-functional theory. Wiley Interdiscip. Rev.: Comput. Mol. Sci.

[51] Lonsdale D. R., Goerigk L. (2020). The one-electron self-interaction error in 74 density
functional approximations: A case study on hydrogenic mono- and dinuclear
systems. Phys. Chem. Chem. Phys.

[52] Perdew J. P., Zunger A. (1981). Self-interaction correction
to density-functional approximations
for many-electron systems. Phys. Rev. B.

[53] Kulik H. J. (2015). Perspective:
Treating electron over-delocalization with the DFT+U method. J. Chem. Phys.

[54] Li C., Zheng X., Su N. Q., Yang W. (2018). Localized orbital scaling
correction for systematic elimination of delocalization error in density
functional approximations. Natl. Sci. Rev.

[55] Perdew, J. P. ; Ruzsinszky, A. ; Sun, J. ; Pederson, M. R. Chapter One - Paradox of Self-Interaction Correction: How Can. Anything So Right Be So Wrong? In Advances In Atomic, Molecular, and Optical Physics; Arimondo, E. ; Lin, C. C. ; Yelin, S. F. , Eds.; Academic Press, 2015; Vol. 64, pp. 1–14.

[56] Ruzsinszky A., Perdew J. P., Csonka G. I., Vydrov O. A., Scuseria G. E. (2006). Spurious
fractional charge on dissociated atoms: Pervasive and resilient self-interaction
error of common density functionals. J. Chem.
Phys.

[57] Medvedev M. G., Bushmarinov I. S., Sun J., Perdew J. P., Lyssenko K. A. (2017). Science.

[58] Kirkpatrick J., McMorrow B., Turban D. H. P., Gaunt A. L., Spencer J. S., Matthews A. G. D. G., Obika A., Thiry L., Fortunato M., Pfau D., Castellanos L. R., Petersen S., Nelson A. W. R., Kohli P., Mori-Sánchez P., Hassabis D., Cohen A. J. (2021). Science.

[59] Kanungo B., Kaplan A. D., Shahi C., Gavini V., Perdew J. P. (2024). Unconventional
Error Cancellation Explains the Success of Hartree-Fock Density Functional
Theory for Barrier Heights. J. Phys. Chem. Lett.

[60] Hernandez, D. J. ; Rettig, A. ; Head-Gordon, M. A New View on Density Corrected DFT: Can One Get a Better Answer for a Good Reason? arXiv 2023.

[61] Gill P. M. W., Johnson B. G., Pople J. A., Frisch M. J. (1992). An investigation
of the performance of a hybrid of Hartree-Fock and density functional
theory. Int. J. Quantum Chem.

[62] Janesko B. G., Scuseria G. E. (2008). Hartree–Fock
orbitals significantly improve
the reaction barrier heights predicted by semilocal density functionals. J. Chem. Phys.

[63] Verma P., Perera A., Bartlett R. J. (2012). Increasing
the applicability of DFT
I: Non-variational correlation corrections from Hartree-Fock DFT for
predicting transition states. Chem. Phys. Lett.

[64] Song S., Vučković S., Kim Y., Yu H., Sim E., Burke K. (2023). Extending density functional
theory with near chemical
accuracy beyond pure water. Nat. Commun.

[65] Santra G., Martin J. M. (2021). What Types of Chemical
Problems Benefit from Density-Corrected
DFT? A Probe Using an Extensive and Chemically Diverse Test Suite. J. Chem. Theory Comput.

[66] Kim M.-C., Sim E., Burke K. (2013). Understanding and Reducing
Errors in Density Functional
Calculations. Phys. Rev. Lett.

[67] Sim E., Song S., Burke K. (2018). Quantifying
Density Errors in DFT. J. Phys. Chem. Lett.

[68] Martín-Fernández C., Harvey J. N. (2021). On the Use of Normalized Metrics for Density Sensitivity
Analysis in DFT. J. Phys. Chem. A.

[69] Mester D., Nagy P. R., Csóka J., Gyevi-Nagy L., Szabó P. B., Horváth R. A., Petrov K., Hégely B., Ladóczki B., Samu G., Lőrincz B. D., Kállay M. (2025). An overview
of developments in the MRCC program system. J. Phys. Chem. A.

[70] Neese F. (2018). Software update:
The ORCA program system, version 4.0. Wiley
Interdiscip. Rev.: Comput. Mol. Sci.

[71] Smith D. G. A., Burns L. A., Simmonett A. C., Parrish R. M., Schieber M. C., Galvelis R., Kraus P., Kruse H., Di Remigio R., Alenaizan A. (2020). PSI4 1.4: Open-source software for high-throughput
quantum chemistry. J. Chem. Phys.

[72] Mester D., Nagy P. R., Kállay M. (2024). Basis-set limit CCSD­(T) energies
for large molecules with local natural orbitals and reduced-scaling
basis-set corrections. J. Chem. Theory Comput.

[73] Kállay M., Nagy P. R., Mester D., Rolik Z., Samu G., Csontos J., Csóka J., Szabó P. B., Gyevi-Nagy L., Hégely B. (2020). The MRCC program system:
Accurate quantum chemistry from water to proteins. J. Chem. Phys.

[74] Kállay, M. ; Nagy, P. R. ; Mester, D. ; Gyevi-Nagy, L. ; Csóka, J. ; Szabó, P. B. ; Rolik, Z. ; Samu, G. ; Csontos, J. ; Hégely, B. , Mrcc, a quantum chemical program suite. https://www.mrcc.hu/ Accessed 1 December 2024.

[75] Riplinger C., Pinski P., Becker U., Valeev E. F., Neese F. (2016). Sparse maps—A
systematic infrastructure for reduced-scaling electronic structure
methods. II. Linear scaling domain based pair natural orbital coupled
cluster theory. J. Chem. Phys.

[76] Ma Q., Werner H.-J. (2018). Scalable Electron Correlation Methods. 5. Parallel
Perturbative Triples Correction for Explicitly Correlated Local Coupled
Cluster with Pair Natural Orbitals. J. Chem.
Theory Comput.

[77] Schmitz G., Hättig C., Tew D. P. (2014). Explicitly correlated PNO-MP2 and
PNO-CCSD and their application to the S66 set and large molecular
systems. Phys. Chem. Chem. Phys.

[78] Gyevi-Nagy L., Kállay M., Nagy P. R. (2020). Integral-direct and parallel implementation
of the CCSD­(T) method: Algorithmic developments and large-scale applications. J. Chem. Theory Comput.

[79] Vučković S., Song S., Kozlowski J., Sim E., Burke K. (2019). Density Functional
Analysis: The Theory of Density-Corrected DFT. J. Chem. Theory Comput.

[80] Johnson E. R., Otero-de-la Roza A., Dale S. G. (2013). Extreme density-driven delocalization
error for a model solvated-electron system. J. Chem. Phys.

[81] Singh A., Fabiano E., Śmiga S. (2025). Understanding
the Core Limitations
of Second-Order Correlation-Based Functionals Through: Functional,
Orbital, and Eigenvalue-Driven Analysis. J.
Chem. Theory Comput.

[82] Nam S., Song S., Sim E., Burke K. (2020). Measuring Density-Driven
Errors Using Kohn-Sham Inversion. J. Chem. Theory
Comput.

[83] Gould T. (2023). Toward routine
Kohn-Sham inversion using the “Lieb-response” approach. J. Chem. Phys.

[84] Perdew J. P., Schmidt K. (2001). Jacob’s ladder
of density functional approximations
for the exchange-correlation energy. AIP Conf.
Proc..

[85] Nagy P. R., Samu G., Kállay M. (2016). An integral-direct
linear-scaling
second-order Møller–Plesset approach. J. Chem. Theory Comput.

[86] Neugebauer H., Pinski P., Grimme S., Neese F., Bursch M. (2023). Assessment
of DLPNO-MP2 Approximations in Double-Hybrid DFT. J. Chem. Theory Comput.

[87] The structure optimizations and intrinsic reaction coordinate calculations were performed at DFT level with Gaussian 16,^125^ while all single point DFT, density sensitivity and LNO-CCSD(T) calculations were carried out using the Mrcc package.^69,73,74^ For the details of geometry optimization, see Section S1. The DFT and LNO-CCSD(T) calculations are performed on the same structures, so the sometimes important geometry-driven errors originating from different optima with different levels of theory are circumvented.^126^ optimized structures and the raw energy data of the single point calculations are presented in additional supplementary files.

[88] Glukhovtsev M. N., Bach R. D., Pross A., Radom L. (1996). The performance of
B3-LYP density functional theory in describing S_N_2 reactions
at saturated carbon. Chem. Phys. Lett.

[89] Adamo C., Barone V. (1998). Exchange functionals
with improved long-range behavior
and adiabatic connection methods without adjustable parameters: The
mPW and mPW1PW models. J. Chem. Phys.

[90] Gritsenko O. V., Ensing B., Schipper P. R. T., Baerends E. J. (2000). Comparison of the
Accurate Kohn–Sham Solution with the Generalized Gradient Approximations
(GGAs) for the S_N_2 Reaction F^–^ + CHF_3_ → FCH_3_ + F^–^: A Qualitative
Rule To Predict Success or Failure of GGAs. J. Phys. Chem. A.

[91] Even though such typical S_N_2 model TSs are well-understood and very well represented in the training data of many DFT models,^27,28,29^ it is worth noting, that SIE-related issues from the self-consistent densities still cannot be eliminated, e.g., solely with better functional parameters.

[92] Cai Y., Liu X., Zhou P., Feng X. (2019). Asymmetric Catalytic Halofunctionalization
of *α*, *β*-Unsaturated
Carbonyl Compounds. J. Org. Chem.

[93] Kristianslund R., Tungen J. E., Hansen T. V. (2019). Catalytic
enantioselective iodolactonization
reactions. Org. Biomol. Chem.

[94] China H., Kumar R., Kikushima K., Dohi T. (2020). Halogen-induced controllable
cyclizations as diverse heterocycle synthetic strategy. Molecules.

[95] Ashtekar, K. D. ; Jaganathan, A. ; Borhan, B. ; Whitehead, D. C. Organic Reactions; John Wiley & Sons, Ltd, 2021; pp. 1–266.

[96] Kozuch S., Martin J. M. L. (2013). Halogen Bonds: Benchmarks and Theoretical Analysis. J. Chem. Theory Comput.

[97] Song S., Vučković S., Sim E., Burke K. (2021). Density Sensitivity
of Empirical Functionals. J. Phys. Chem. Lett.

[98] Anderson L. N., Aquino F. W., Raeber A. E., Chen X., Wong B. M. (2018). Halogen
Bonding Interactions: Revised Benchmarks and a New Assessment of Exchange
vs Dispersion. J. Chem. Theory Comput.

[99] Grimme S., Antony J., Ehrlich S., Krieg H. (2010). A consistent and accurate
ab initio parametrization of density functional dispersion correction
(DFT-D) for the 94 elements H-Pu. J. Chem. Phys.

[100] Caldeweyher E., Ehlert S., Hansen A., Neugebauer H., Spicher S., Bannwarth C., Grimme S. (2019). A generally applicable
atomic-charge dependent London dispersion correction. J. Chem. Phys.

[101] Vydrov O.
A., Van Voorhis T. (2010). Nonlocal van
der Waals density functional:
The simpler the better. J. Chem. Phys.

[102] Tkachenko N. V., Head-Gordon M. (2024). Smoother Semiclassical Dispersion
for Density Functional Theory via D3S: Understanding and Addressing
Unphysical Minima in the D3 Dispersion Correction Model. J. Chem. Theory Comput.

[103] Tkachenko N. V., Dittmer L. B., Tomann R., Head-Gordon M. (2024). Smooth Dispersion
Is Physically Appropriate: Assessing and Amending the D4 Dispersion
Model. J. Phys. Chem. Lett.

[104] Since recent studies revealed that halogen bonds can be sensitive to SIE,^127^ **B-prec** could be affected as well. The DFT errors and density sensitivities for **B-prec** indicate some, but not larger than 3 kcal/mol density sensitivity in the stability of the precomplex (Table S4). This is probably due to the relatively low polarizability of chlorine among the halogens and the relative weakness of the halogen bond due to the weaker electron acceptor and donor ability of chlorine and the π-bond, respectively.

[105] Mayer I. (1983). Charge, bond order and valence in the ab initio SCF
theory. Chem. Phys. Lett.

[106] Aynetdinova D., Callens M. C., Hicks H. B., Poh C. Y. X., Shennan B. D. A., Boyd A. M., Lim Z. H., Leitch J. A., Dixon D. J. (2021). Installing the “magic methyl”
- C–H methylation in synthesis. Chem.
Soc. Rev.

[107] Chen Y. (2019). Recent Advances
in Methylation: A Guide for Selecting Methylation
Reagents. Chem. - Eur. J..

[108] Barreiro E. J., Kümmerle A. E., Fraga C. A. M. (2011). The Methylation
Effect in Medicinal Chemistry. Chem. Rev.

[109] Siitonen J. H., Csókas D., Pápai I., Pihko P. M. (2020). Total Synthesis of Stemoamide, 9a-epi-Stemoamide,
and
9a, 10-epi-Stemoamide: Divergent Stereochemistry of the Final Methylation
Steps. Synlett.

[110] This is indeed the case if we consider the about 5 kcal/mol higher GGA-level density sensitivity measure of iodomethane compared to the iodide-ion.

[111] For further analysis regarding the reactant state as reference (Figure S21) as well as the HF, MP2, and post-MP2 contributions (Figure S23) we refer to Section S2.3.

[112] Reyes E., Uria U., Vicario J. L., Carrillo L. (2016). The catalytic,
enantioselective Michael reaction. Org. React.

[113] Das T., Mohapatra S., Mishra N. P., Nayak S., Raiguru B. P. (2021). Recent
Advances in Organocatalytic Asymmetric Michael Addition Reactions
to *α*, *β*-Unsaturated
Nitroolefins. ChemistrySelect.

[114] Pasuparthy S. D., Maiti B. (2022). Enantioselective Organocatalytic
Michael Addition Reactions Catalyzed by Proline/Prolinol/Supported
Proline based Organocatalysts: An Overview. ChemistrySelect.

[115] Zhang Y., Wang W. (2012). Recent advances in organocatalytic
asymmetric Michael reactions. Catal. Sci. Technol.

[116] The respective correlation coefficients are 0.21, 0.04, and 0.24 for **D-oo**, **D-ts** _ **2** _ and **D-cb**, respectively, compared to 0.83 obtained for **D-ts** _ **1** _ inTable S11.

[117] In transition state **D-ts** _ **1** _, C-C bond 7 is approximately halfway-formed and the C-O bond 8 is not significant yet (BO7 is 0.481 and BO8 is 0.067).

[118] Werner H.-J., Knowles P. J., Knizia G., Manby F. R., Schütz M. (2012). Molpro: a general-purpose quantum
chemistry program
package. Wiley Interdiscip. Rev.: Comput. Mol.
Sci.

[119] Sylvetsky N., Banerjee A., Alonso M., Martin J. M. L. (2020). Performance
of Localized Coupled Cluster Methods in a Moderately Strong Correlation
Regime: Hückel–Möbius Interconversions in Expanded
Porphyrins. J. Chem. Theory Comput.

[120] Kříž K., Řezáč J. (2020). Benchmarking
of Semiempirical Quantum-Mechanical Methods on Systems Relevant to
Computer-Aided Drug Design. J. Chem. Inf. Model.

[121] Semidalas E., Martin J. M. L. (2022). The MOBH35 metal-organic
barrier
heights reconsidered: performance of local-orbital coupled cluster
approaches in different static correlation regimes. J. Chem. Theory Comput.

[122] Maurer L. R., Bursch M., Grimme S., Hansen A. (2021). Assessing
Density Functional Theory for Chemically Relevant Open-Shell Transition
Metal Reactions. J. Chem. Theory Comput.

[123] Kazakov A., Paulechka E. (2025). Accurate Enthalpies
of Formation
for Bioactive Compounds from High-Level Ab Initio Calculations with
Detailed Conformational Treatment: A Case of Cannabinoids. J. Chem. Theory Comput.

[124] Szabó P. B., Csóka J., Kállay M., Nagy P. R. (2023). Linear-scaling local
natural orbital CCSD­(T) approach
for open-shell systems: algorithm, benchmarks, and large-scale applications. J. Chem. Theory Comput.

[125] Frisch, M. J. ; Trucks, G. W. ; Schlegel, H. B. ; Scuseria, G. E. ; Robb, M. A. ; Cheeseman, J. R. ; Scalmani, G. ; Barone, V. ; Mennucci, B. ; Petersson, G. A. , Gaussian 16 Revision C.01.; Gaussian, Inc., 2016.

[126] Vučković S., Burke K. (2020). Quantifying and Understanding
Errors in Molecular Geometries. J. Phys. Chem.
Lett.

[127] Kim Y., Song S., Sim E., Burke K. (2019). Halogen and Chalcogen
Binding Dominated by Density-Driven Errors. J. Phys. Chem. Lett.

