# Supplementary material for: Understanding DFT Uncertainties for More Reliable Reactivity Predictions by Advancing the Analysis of Error Sources
Source: J Chem Theory Comput. 2025 Sep 18;21(19):9483–97. doi: 10.1021/acs.jctc.5c00985 (PMC12529897; doi:10.1021/acs.jctc.5c00985)
Supplement: Supplementary file 1 [file ct5c00985_si_001.pdf]

Supporting information for publication: Understanding DFT uncertainties for more reliable reactivity predictions by advancing the analysis of error sources

Gergely Laczkó,[†,‡] Imre Pápai,[*,†] and Péter R. Nagy[*,¶,§,‖]

†*Institute of Organic Chemistry, HUN-REN Research Centre for Natural Sciences, Magyar Tudósok Körútja 2, H-1117 Budapest, Hungary*

‡*Hevesy György PhD School of Chemistry, Eötvös Loránd University, P.O. Box 32, Budapest, H-1518, Hungary*

¶*Department of Physical Chemistry and Materials Science, Faculty of Chemical Technology and Biotechnology, Budapest University of Technology and Economics, Műegyetem rkp. 3., H-1111 Budapest, Hungary,*

§*HUN-REN-BME Quantum Chemistry Research Group, Műegyetem rkp. 3., H-1111 Budapest, Hungary*

‖*MTA-BME Lendület Quantum Chemistry Research Group, Műegyetem rkp. 3., H-1111 Budapest, Hungary*

E-mail: papai.imre@ttk.hu; nagy.peter@vbk.bme.hu

Contents

S1 Computational Details **3**

S2 Additional tables, figures and analysis **11**

S2.1 Nucleophilic substitution 11

S2.2 Halocyclization 16

S2.2.1 Varying the HFx ratio 18

S2.2.2 The effect of dispersion correction 20

S2.2.3 Analysis along the RC 23

S2.3 Methylation 24

S2.3.1 Anti methylation 32

S2.3.2 Syn VS anti methylation 35

S2.4 Michael addition 36

S3 Statistics **43**

S4 Sample input files **45**

References **48**

S1 Computational Details

For the LNO-CCSD(T) calculations, we applied the aug-cc-pVXZ (X=T, Q, and 5) correlation consistent basis sets of Dunning[1–3] (with the effective core potential for iodine[4]). The HF and LNO-CCSD(T) correlation energies are extrapolated towards the complete basis set (CBS) limit via:[5,6]

$$E^{\mathrm{HF}}_{X(X-1)} = E^{\mathrm{HF}}_{X} + \frac{(X+1)\left(E^{\mathrm{HF}}_{X} - E^{\mathrm{HF}}_{X-1}\right)}{X \exp\left[\gamma\left(\sqrt{X} - \sqrt{X-1}\right)\right]}, \tag{1}$$

$$E^{\mathrm{corr}}_{X(X+1)} = \frac{X^3 E^{\mathrm{corr}}_{X} - (X+1)^3 E^{\mathrm{corr}}_{X+1}}{X^3 - (X+1)^3}. \tag{2}$$

In case of CBS(T,Q) extrapolation, we employed $X = 4$ and $\gamma = 6.57$ in Eq. (1) and $X = 3$ in Eq. (2). To accelerate the convergence of the LNO-CCSD(T) results, we also used a recent extrapolation method to approach the local approximation free (LAF) limit of CCSD(T).[7,8] For example, one can generally recommend

$$E_{\text{N-T}} = E_{\text{Tight}} + 0.5(E_{\text{Tight}} - E_{\text{Normal}}), \tag{3}$$

where E_{Tight} and E_{Normal} are the LNO-CCSD(T) correlation energies with the *Tight* and *Normal* LNO threshold sets, respectively. Moreover, the step size of this LAF extrapolation provides an estimation for the remaining LNO error, that is $\pm 0.5(E_{\text{Tight}} - E_{\text{Normal}})$ error bar can be employed. For the final LNO-CCSD(T) energies approximating CCSD(T)/CBS, an efficient composite scheme[8] is employed exploiting both CBS and LAF extrapolations:

$$E^{\text{CBS(T,Q)}}_{\text{N-T LNO-CCSD(T)}} = E^{\text{CBS(T,Q)}}_{\text{Normal}} + E^{\text{TZ}}_{\text{N-T}} - E^{\text{TZ}}_{\text{Normal}}. \tag{4}$$

The computed and the extrapolated energies in the important structures of reactions **A**, **C**, **D** and **B** are shown in Figures S1, S2, S3, and S4, respectively. For the structures in **C**, a representative intermediate of **D-cb**, as well as for reaction **B** below, we also carried out

calculations with even more accurate (aug-cc-pV5Z and *very Tight* LNO) settings than what is used in Eq. 4 to verify its validity. It is clear from these convergence studies that the LNO approximation is converged at least to a few tenths kcal/mol. Although the difference of the aug-cc-pVTZ and aug-cc-pVQZ results are somewhat larger, this is in accord with our general experience showing notable basis set superposition effects with the diffuse triple-ζ bases.[8] However, the basis set convergence of the CBS(T,Q) extrapolated results is convincing, as the basis convergence uncertainty of ca. a few tenths of a kcal/mol is considerably smaller than the difference of CBS(T,Q) and aug-cc-pVQZ results. This is further verified by the very close agreement of the CBS(T,Q) and CBS(Q,5) results (Figures S2–S4).

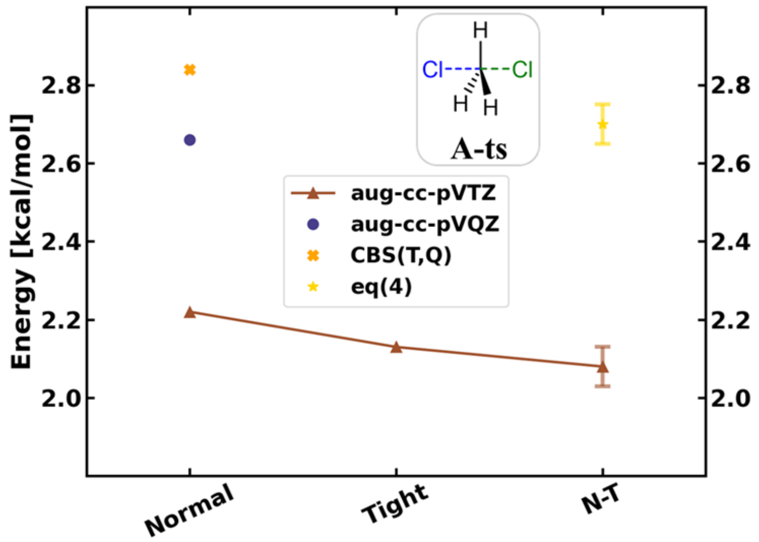

Figure S1: LNO-CCSD(T) results for reaction **A** with various basis sets, LNO settings, and consequent extrapolations in the barrier height of the nucleophilic substitution (with respect to separated reactants, see Scheme 1).

While this composite approach in Eq. 4 was found reliable in a wide range of applications,[8] we even more carefully assessed it for the **B-ts** barrier, since this depends on the largest and most complicated system in this study. To that end, systematically converging series of aug-cc-pVXZ basis sets with X= T, Q, and 5 as well as of S = *Normal, Tight, veryTight, veryveryTight* LNO setting were employed (Figure S4). Moreover, we utilized

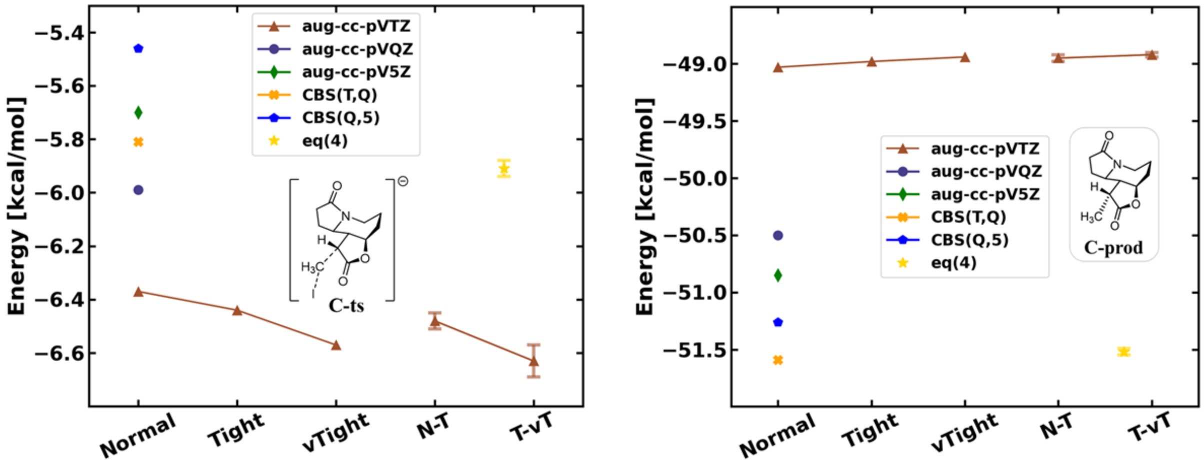

Figure S2: LNO-CCSD(T) results for reaction **C** with various basis sets, LNO settings, and consequent extrapolations in the barrier height and reaction energy of the methylation (with respect to separated reactants, see Scheme 3).

LAF extrapolations for the general S LNO settings as:[7,8]

$$E_{\text{S-(S+1)}} = E_{\text{S+1}} + 0.5(E_{\text{S+1}} - E_{\text{S}}). \tag{5}$$

The barrier height of the halocyclization (Figure S4) shows notable basis set dependence, if we consider the difference between the aug-cc-pVXZ values with X= T, Q, and 5. The basis set incompleteness error at that aug-cc-pVTZ basis set still amounts to ca. 6 kcal/mol. However, the CBS(T,Q) and CBS(Q,5) extrapolated results are practically identical, verifying the high-quality of CBS(T,Q) in this study.

Using the aug-cc-pVTZ basis set, the N-T and the *veryTight-veryveryTight* (vT-vvT) LAF extrapolated barriers differ by 0.4 kcal/mol, which is consistent with the ± 0.3 and ± 0.1 kcal/mol LNO error bars corresponding to the N-T and vT-vvT LAF extrapolated results. As the *Normal*, *Tight*, and N-T convergence pattern is fairly independent of the basis set, the composite form of Eq. (4) is justified. This is also confirmed by the excellent agreement of this N-T LNO-CCSD(T)/CBS(T,Q) barrier height (yellow star in Figure S4) compared to the best converged, but more expensive results. Let us note, that the case of this halocyclization barrier was extensively analyzed in Sect. 3.3, S6 of Ref. 8 also against canonical CCSD(T),

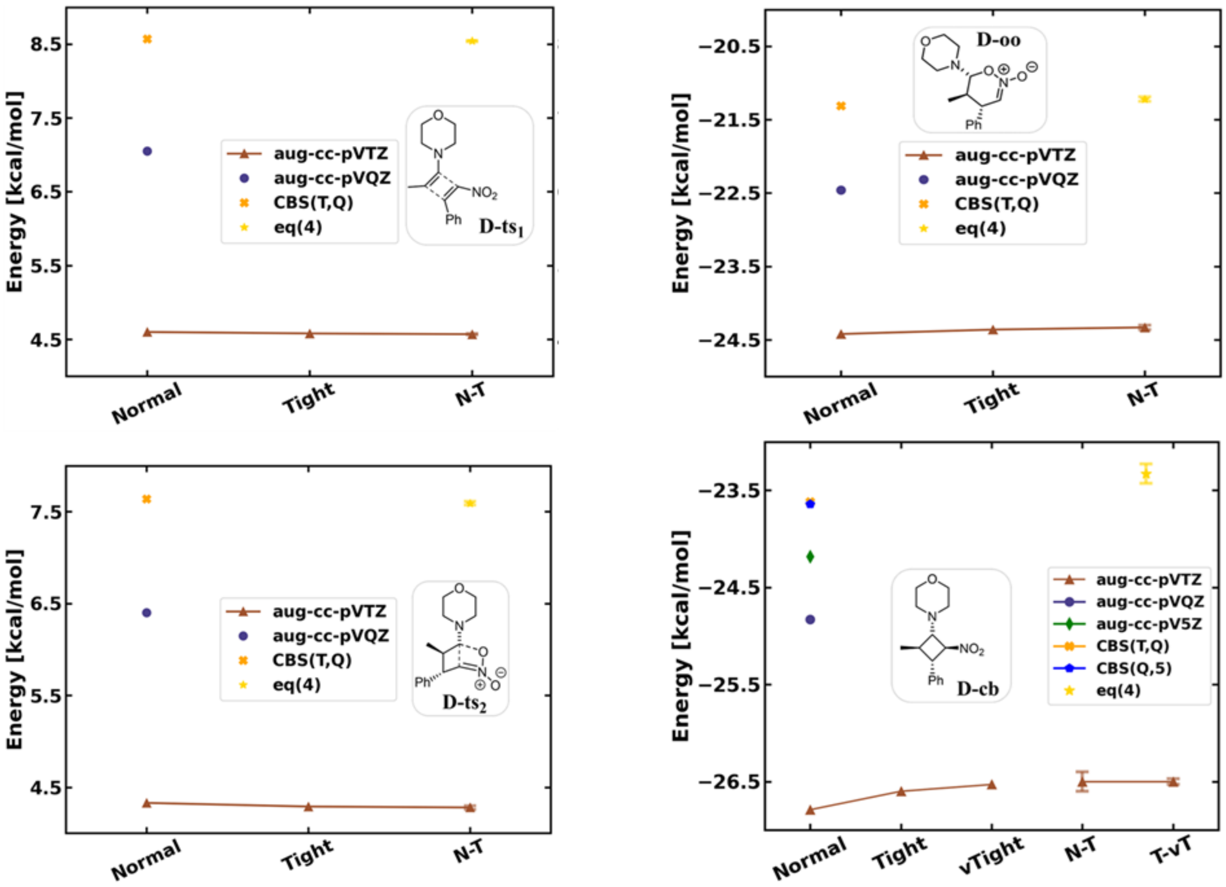

Figure S3: LNO-CCSD(T) results for reaction **D** with various basis sets, LNO settings, and consequent extrapolations in the important stationary points of the Michael addition (with respect to separated reactants, see Scheme 4).

indicating close to 99.999% accurate correlation energies and ca. 0.1 kcal/mol LNO error in the barrier.

Using our highly-optimized MPI parallel code in MRCC,[9] we carried out canonical (density-fitting based) DF-CCSD(T) calculations to further verify the appropriateness of the LNO approach. For the important structures of reactions **C** and **D** the use of the def2-TZVPPD basis set is still feasible, which would be not possible for the larger **B-ts** (hence the detailed test in Figure S4 up to *veryveryTight* LNO settings). Most of the time, the convergence tests with (*Loose*), *Normal* and *Tight* LNO thresholds are sufficient.[8] When feasible, more expensive tests against DF-CCSD(T) can provide additional evidence and here they are performed upon a request during the revision of the manuscript. The DF-CCSD(T)/def2-TZVPPD are

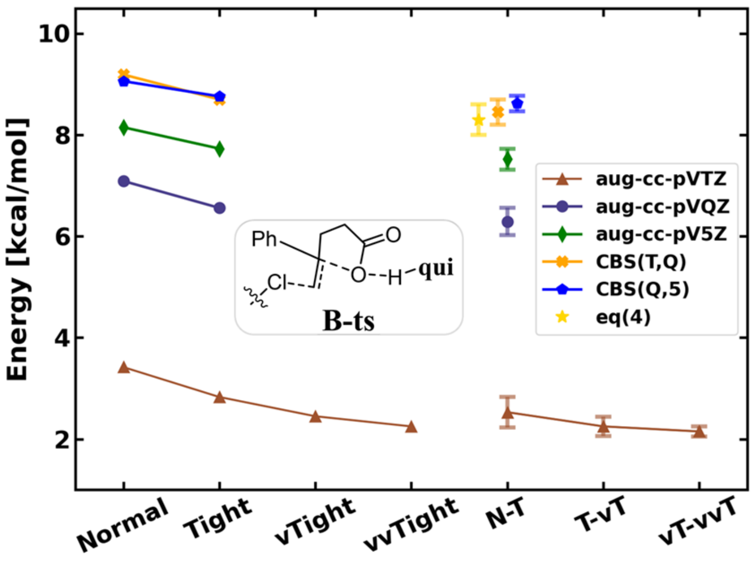

Figure S4: LNO-CCSD(T) results with various basis sets, LNO settings, and consequent extrapolations in the barrier height of the halocyclization reaction **B**.

compared to the LNO-CCSD(T)/def2-TZVPPD result with various thresholds in Table S1. The difference between canonical DF-CCSD(T) and LNO-CCSD(T) are 0.0–0.3 kcal/mol already with the *Normal* settings and the N-T extrapolation is spot on.

We briefly note that no indication (e.g., in HF and CCSD iteration convergence or in HF and CCSD quantities, such as orbital energies, amplitude size and amplitude based multi-reference indicators) was found to suggest any multi-reference character, single-reference CCSD(T) appear to be perfectly applicable, as expected.

Table S1: Stabilities of important structures in reactions **C** and **D** with respect to the corresponding reactants obtained with LNO-CCSD(T) (various thresholds) and DF-CCSD(T) with the def2-TZVPPD basis set [kcal/mol].

	Normal	Tight	vTight	N-T	T-vT	**canonical**
**C-ts**	-6.8	-6.9	-6.9	-6.9	-7.0	**-6.8**
**C-prod**	-50.5	-50.2	-50.2	-50.1	-50.1	**-50.1**
**D-ts$_1$**	5.3	5.2	5.2	5.2	5.1	**5.0**
**D-oo**	-23.8	-23.7	-23.6	-23.7	-23.6	**-23.7**
**D-ts$_2$**	5.1	5.2	5.2	5.2	5.1	**5.1**
**D-cb**	-26.6	-26.4	-26.3	-26.3	-26.3	**-26.3**

Therefore, we employ the composite $E^{\text{CBS(T,Q)}}_{\text{N-T LNO-CCSD(T)}}$ expression of Eq. (4) throughout this work. The frozen core approximation was employed for the core electrons. Regarding iodine, its first three shells were described with the effective core potential and the correlation of the 4d sub-valence and all valence electrons were included. All LNO-CCSD(T) as well as single point DFT calculations were carried out with the MRCC program.[10–12]

Altogether, 24 functionals were chosen for our benchmark, which included 4–4 GGA and mGGA as well as 5–5–5 hybrid, RSH and DH methods. Unless noted otherwise, we applied dispersion correction. The D4[13] corrections was added to all functionals for which it was available and the D3[14] scheme was applied in the remaining cases, both developed by Grimme and co-workers. Some methods with the non-local VV10[15] dispersion correction were also considered. The important functional parameters and their references are collected in Table S2.

For single point energy calculations, the def2-TZVPP basis set was applied with all the functionals.[42] For some of the most important structures, we carried out calculations also with the def2-QZVPP basis. The errors obtained with the different basis sets are compared in Figure S5. Using an even larger basis set does not have a notable effect on the errors with respect to the LNO-CCSD(T)/CBS stabilities from the perspective of the investigated trends. The most complicated case of **B-ts** was again more carefully analyzed, i.e. we calculated the barrier height on the def2-QZVPP basis set with all the investigated functionals (Figure S6). These results further support that no significant basis set dependence is expected for the DFT methods.

Optimizations and intrinsic reaction coordinate (IRC) calculations were carried out with Gaussian 16.[43] Stationary points were searched at the M06-2X/def2-SVP level of theory for the nucleophilic addition and the halocyclization. The optimized structures corresponding to the methylation and the Michael addition were taken from Refs. 44 and 45, respectively. These structures were optimized with the ωB97X-D functional, applying the def2-SVP basis set in the methylation and the 6-311g(d,p) basis for the Michael addition. The employed

Table S2: Rung, ratio of exact exchange, applied dispersion correction and relevant publications of the functionals studied in this work.

	rung	% HFx	dispersion	references
BP86-D4	GGA	0	D4[13]	16, 17
BLYP-D4	GGA	0	D4	16, 18
PW91-D4	GGA	0	D3[14]	19
PBE-D4	GGA	0	D4	20
SCAN-D4	mGGA	0	D4	21
TPSS-D4	mGGA	0	D4	22
B97M-V	mGGA	0	VV10[15]	23
M06-L-D3	mGGA	0	D3	24
TPSSh-D4	H	10	D4	25
B3LYP-D4	H	20	D4	26
PBE0-D4	H	25	D4	27
MN15-D3	H	44	D3	28
BHLYP-D4	H	50	D4	29
M06-2X-D3	H	54	D3	30
ωB97X-D4	RSH	22-100	D4	31, 32
ωB97X-V	RSH	16.7-100	VV10	33
ωB97M-V	RSH	15-100	VV10	34
LC-ωPBE-D3	RSH	0-100	D3	35
CAM-B3LYP-D4	RSH	19-65	D4	36
B2PLYP-D4	DH	53	D4	37
DSD-PBEP86-D4	DH	69	D4	38
revDSD-PBEP86-D4	DH	69	D4	39
DRPA75-D3	DH	75	D3	40
DSD-PBEP86-dRPA75-D4	DH	75	D4	41

structures, DFT and LNO-CCSD(T) energies are provided in supplementary files. The Mayer bond order[46] was employed for all reactions as reaction coordinate.

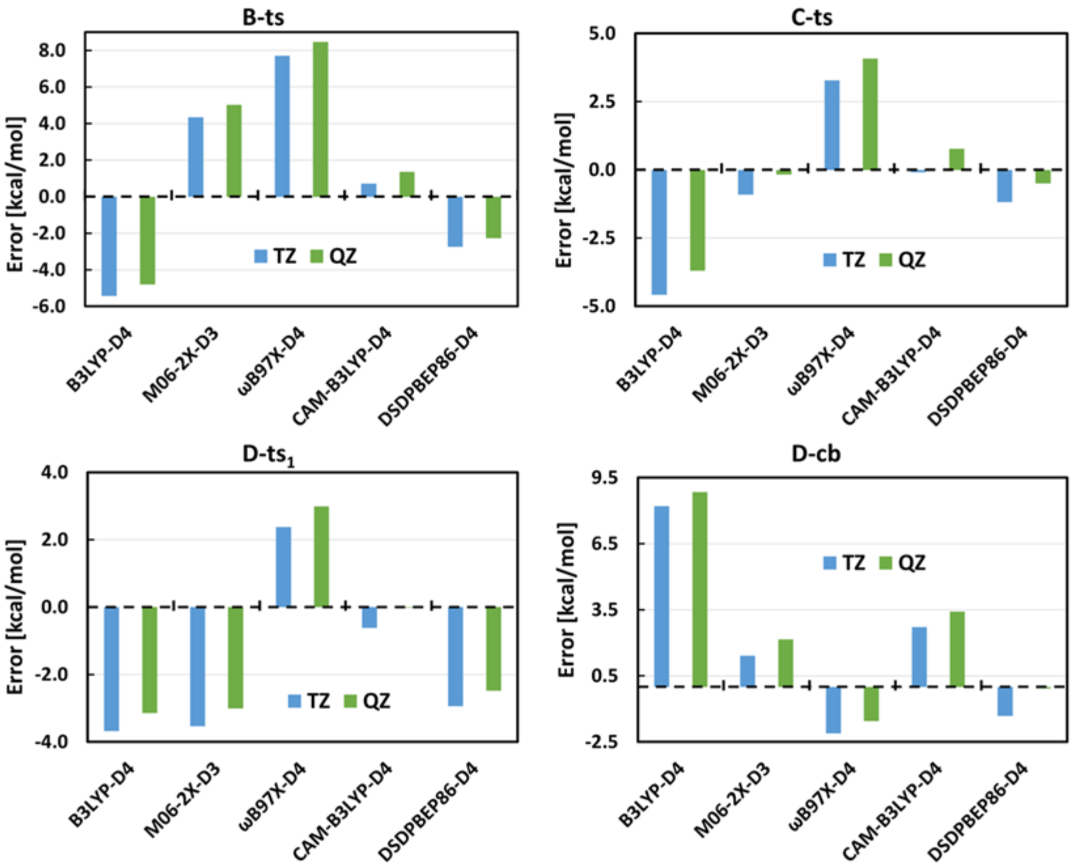

Figure S5: Errors of some representative functionals [with respect to LNO-CCSD(T)] in the stability of important structures [with respect to separated reactants] obtained with def2-TZVPP (TZ) or def2-QZVPP (QZ) basis set.

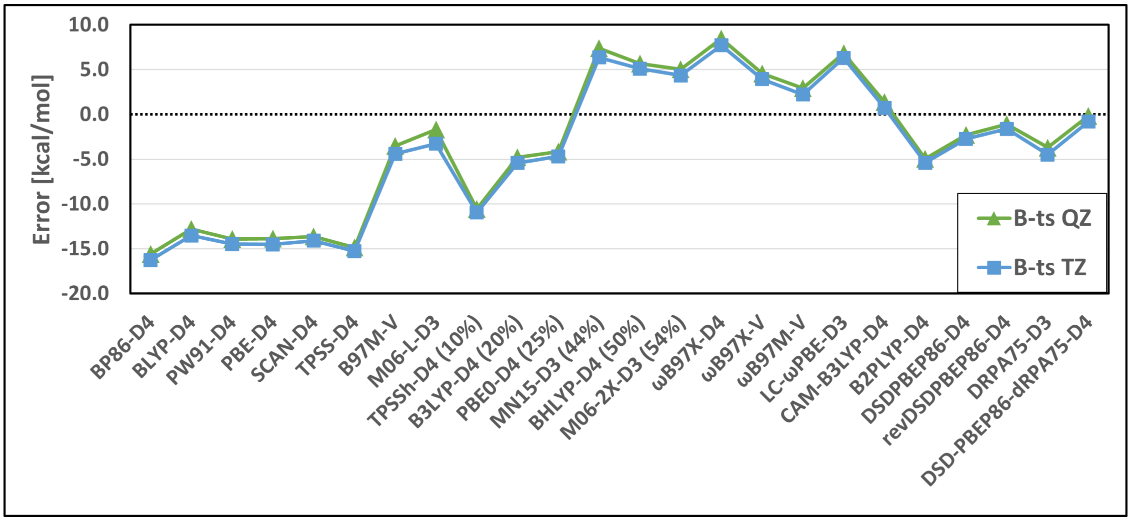

Figure S6: Errors of functionals [with respect to LNO-CCSD(T)] in the barrier of reaction **B** (see Scheme 2) obtained with def2-TZVPP (TZ) or def2-QZVPP (QZ) basis set.

S2 Additional tables, figures and analysis

S2.1 Nucleophilic substitution

The signed error and density sensitivity of each investigated functional are plotted in Figure S7 and are collected into Table S3. The contribution of the applied dispersion correction for each functional is also collected in Table S3. Compared to the LNO-CCSD(T)/CBS reference, we find the stability of pre-complex **A-prec** with the 24 functionals generally accurate within −1 to −4 kcal/mol. In contrast, up to 12 kcal/mol errors are found in the barrier height of **A-ts**.

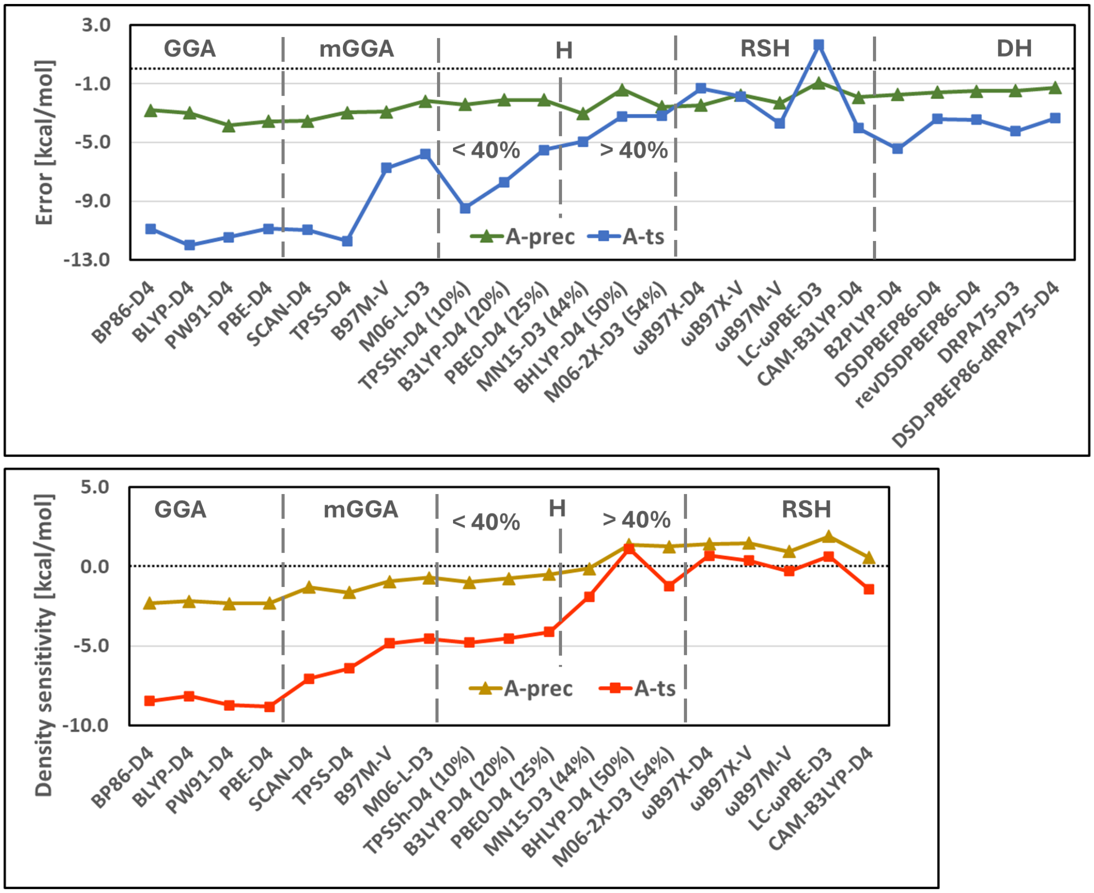

Figure S7: Signed errors (top) and density sensitivities (bottom) in **reaction A** (nucleophilic substitution, Scheme 1.) The errors and density sensitivities are calculated in the stabilities with respect to the separated reactants.

Table S3: Signed errors, density sensitivities (S^{DFT}) and the contribution of the applied dispersion-corrections (D) in **reaction A** (nucleophilic substitution, Scheme 1) in kcal/mol. The errors and density sensitivities are calculated in the stabilities with respect to the separated reactants.

	Error (**A-prec**)	Error (**A-ts**)	S^{DFT} (**A-prec**)	S^{DFT} (**A-ts**)	D (**A-prec**)	D (**A-ts**)
BP86-D4	-2.8	-10.9	-2.3	-8.5	-1.0	-1.1
BLYP-D4	-3.0	-12.0	-2.2	-8.2	-1.4	-1.7
PW91-D4	-3.9	-11.5	-2.3	-8.7	-0.5	-0.6
PBE-D4	-3.6	-10.9	-2.3	-8.8	-0.7	-0.8
SCAN-D4	-3.5	-11.0	-1.3	-7.1	-0.1	-0.1
TPSS-D4	-3.0	-11.7	-1.6	-6.4	-0.9	-1.1
B97M-V	-2.9	-6.7	-0.9	-4.8	-1.4	-1.6
M06-L-D3	-2.2	-5.8	-0.7	-4.5	0.0	0.0
TPSSh-D4 (10%)	-2.4	-9.5	-1.0	-4.8	-0.9	-1.0
B3LYP-D4 (20%)	-2.1	-7.7	-0.8	-4.5	-1.1	-1.3
PBE0-D4 (25%)	-2.1	-5.5	-0.5	-4.1	-0.6	-0.7
MN15-D3 (44%)	-3.0	-4.9	-0.1	-1.9	0.0	0.0
BHLYP-D4 (50%)	-1.4	-3.2	1.4	1.1	-0.8	-1.0
M06-2X-D3 (54%)	-2.6	-3.2	1.3	-1.2	0.0	0.0
ωB97X-D4	-2.5	-1.3	1.4	0.7	-0.1	-0.1
ωB97X-V	-1.8	-1.9	1.5	0.4	-1.4	-1.6
ωB97M-V	-2.3	-3.7	0.9	-0.3	-1.4	-1.6
LC-ωPBE-D3	-0.9	1.6	1.9	0.6	-0.9	-1.0
CAM-B3LYP-D4	-1.9	-4.0	0.6	-1.4	-0.6	-0.7
B2PLYP-D4	-1.7	-5.4			-0.5	-0.6
DSD-PBEP86-D4	-1.6	-3.4			-0.1	-0.2
revDSD-PBEP86-D4	-1.5	-3.5			-0.3	-0.4
DRPA75-D3	-1.5	-4.2			-0.6	-0.8
DSD-PBEP86-dRPA75-D4	-1.3	-3.4			-0.4	-0.5

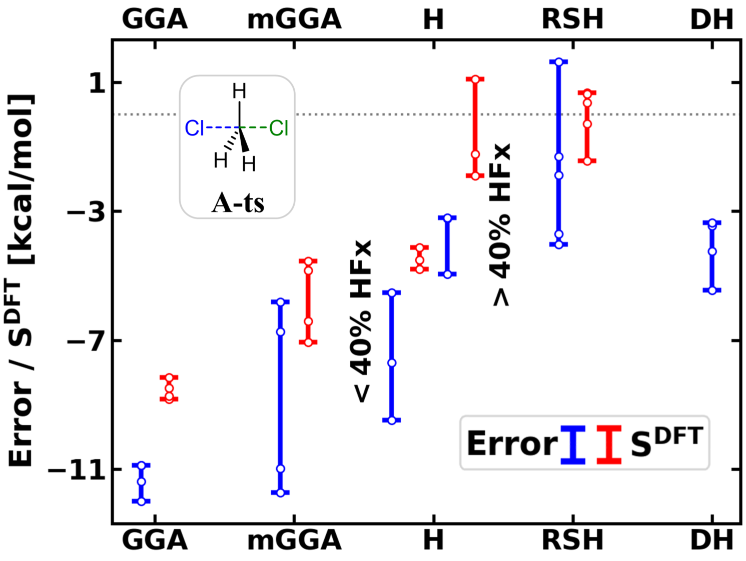

Figure S8: Signed error of various functionals with respect to LNO-CCSD(T) reference (left bars, blue) and S^{DFT} density sensitivity (eq 4, right bars, red) of functionals in the transition state of the nucleophilic substitution (with respect to separated reactants, see Scheme 1). Results are plotted with bars corresponding to each functional category (defined in Section 2) and white dots represent the individual results. The values corresponding to each functional are plotted individually in Figure S7 and given in Table S3.

In Figure S8, we plotted the error (blue) and density sensitivity (red) of each functional for the barrier height. For simplicity, the results are arranged according to the noted DFT rungs/groups, while the individual results are collected in Figure S7 and in Table S3. The DFT errors are generally negative (with one exception) and systematically decrease with the increased sophistication of the functionals along Jacob's ladder. Within the hybrid methods, the error is reduced when more HFx is introduced, showing a correlation with the expected decrease in SIE.

Having a closer look at Figure S8, both the error and density sensitivity of pure (m)GGA functionals are sizable and correlate well with each other. The density sensitivities of mGGAs are somewhat smaller than for GGAs, and advanced mGGAs among those (M06-L and B97M-V) provide somewhat improved barriers, probably partly because they were trained to reproduce barrier heights of similar reactions. The errors and density sensitivities of hybrids with a low amount of HF exchange are not much better than with (m)GGAs, suggesting

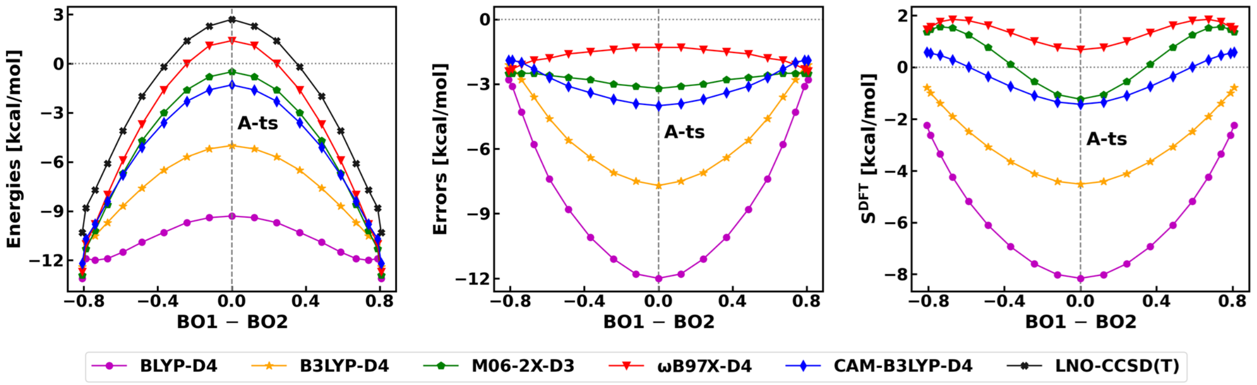

Figure S9: left: Electronic energies with various methods along the reaction coordinate (RC) of the nucleophilic substitution (with respect to separated reactants, see Scheme 1). The difference of the bond orders (BO) of the forming and breaking C-Cl bonds denoted by 1 and 2 in Scheme 1 is chosen as reaction coordinate, using the Mayer Bond Order[46] as for all other reactions in this study. middle: Signed errors of functionals with respect to LNO-CCSD(T)/CBS results along the RC. right: Density sensitivities of functionals along the RC.

that 10-25% HFx is not sufficient to circumvent the SIE. Compared to those, hybrids with a large amount of exact exchange, RSHs and DHs show ca −5 to 2 kcal/mol errors. Since the corresponding density sensitivities are smaller, these errors cannot be characterized as mostly density-driven. We note that the effect and size of dispersion corrections were also checked but they turned out to be insignificant, which is consistent with the small size of the system.

Since the DFT barrier errors for this nucleophilic substitution TS are well explained by SIE and there appears to be only one significant source of DFT error, one might conclude the analysis at this point. However, taking advantage of the relative simplicity of this well-known example, we also show the proposed analysis along the RC. Inspecting Figure S9, the electronic energies (left panel) and the corresponding errors (middle panel) show consistent negative errors along most of the RC, peaking at the TS point. This especially holds for the methods with no or low HFx content, where the density sensitivity curves (right panel) mirror the shape of the error curves. In comparison, the RSH methods and M06-2X-D3 with 54% HFx content provide a fairly consistent performance along the RC, in accordance with their relatively small density sensitivity values.

We also monitored the correlation energy contributions along the RC and found that they were not large and nearly constant during the reaction (Figure S10). The CCSD(T) correlation energy is mostly captured at the level of second-order perturbation theory (MP2). All in all, for this simpler case, the error analysis along the RC provides the same conclusions for all points as for the TS in Figure S8.

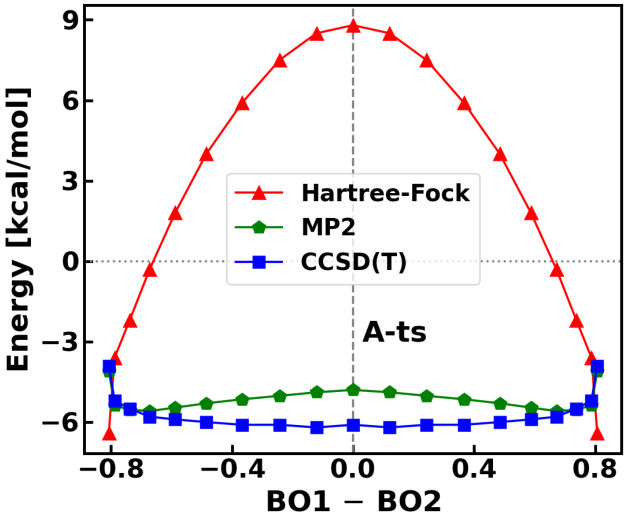

Figure S10: Correlation and Hartree–Fock contributions in **reaction A** (nucleophilic substitution, Scheme 1) to the LNO-CCSD(T) energy along the reaction coordinate. The separated reactants are taken as reference state.

S2.2 Halocyclization

The signed error and density sensitivity of each investigated functional are plotted in Figure S11 and are collected into Table S4. These data correspond to Figure 2 in the article. The error in the reaction energy is also added to Table S4. The effect of the dispersion correction is demonstrated in Table S5. The total correlation and Hartree–Fock contributions to the LNO-CCSD(T) energy along the reaction coordinate are plotted in Figure S16.

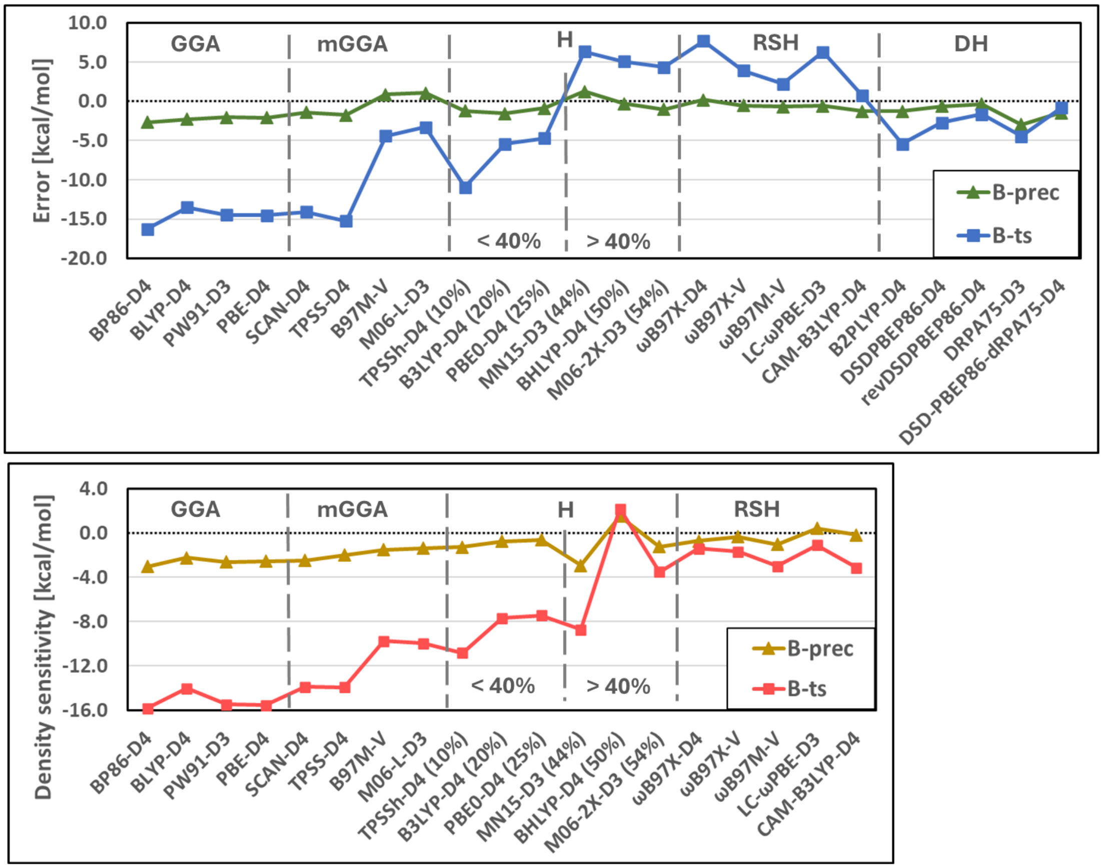

Figure S11: Signed errors (top) and density sensitivities (bottom) in **reaction B** (halocyclization, Scheme 2). The errors and density sensitivities are calculated in the stabilities with respect to the separated reactants.

Table S4: Signed errors, density sensitivities (S^{DFT}) in **reaction B** (halocyclization, Scheme 2) in kcal/mol. The errors and density sensitivities are calculated in the stabilities with respect to the separated reactants.

	Error (**B-prec**)	Error (**B-ts**)	S^{DFT} (**B-prec**)	S^{DFT} (**B-ts**)	Error (**B-prod**)
BP86-D4	-2.7	-16.3	-3.0	-15.8	2.9
BLYP-D4	-2.3	-13.5	-2.2	-14.0	7.0
PW91-D4	-2.0	-14.5	-2.6	-15.5	3.4
PBE-D4	-2.1	-14.5	-2.5	-15.6	2.5
SCAN-D4	-1.4	-14.1	-2.5	-13.9	-2.0
TPSS-D4	-1.7	-15.3	-2.0	-13.9	1.7
B97M-V	0.9	-4.4	-1.5	-9.8	0.5
M06-L-D3	1.1	-3.3	-1.4	-10.0	2.1
TPSSh-D4 (10%)	-1.2	-11.0	-1.3	-10.8	0.0
B3LYP-D4 (20%)	-1.5	-5.4	-0.8	-7.7	3.1
PBE0-D4 (25%)	-0.9	-4.7	-0.6	-7.4	-2.1
MN15-D3 (44%)	1.2	6.4	-2.9	-8.7	-0.6
BHLYP-D4 (50%)	-0.3	5.1	1.5	2.1	-1.2
M06-2X-D3 (54%)	-1.0	4.4	-1.2	-3.5	0.3
ωB97X-D4	0.2	7.7	-0.7	-1.4	-1.5
ωB97X-V	-0.5	3.9	-0.4	-1.7	-4.0
ωB97M-V	-0.7	2.2	-1.0	-3.0	-1.7
LC-ωPBE-D3	-0.6	6.3	0.4	-1.1	-6.5
CAM-B3LYP-D4	-1.3	0.7	-0.2	-3.2	-0.1
B2PLYP-D4	-1.3	-5.4			1.5
DSD-PBEP86-D4	-0.6	-2.7			-0.7
revDSD-PBEP86-D4	-0.3	-1.6			-0.3
DRPA75-D3	-3.0	-4.5			0.9
DSD-PBEP86-dRPA75-D4	-1.4	-0.8			-0.1

S2.2.1 Varying the HFx ratio

Since we showed that the large error of some popular hybrid methods originates from their low amount of Hartree–Fock exchange, we investigated whether their accuracy can be improved by optimizing their HFx content (Figure S12). For the functionals with lower HFx ratio (B3LYP-D4 20% and PBE0-D4 25%), increased amount of HFx counteracting the SIE improved the barrier heights with optima around 27% and 40% HF exchange, respectively. Then, a further increase in the repulsive HFx content led to positive barrier errors. Similarly, the original 50% and 54% HFx content of BHLYP-D4 and M06-2X-D3 are over their optimal values, explaining both their low density sensitivity and positive barrier errors. However, the other key quantities (stability of the precomplex and the products) do not improve consistently with the barrier heights upon varying the HFx content or even deteriorate. Therefore, the optimization of the HF exchange parameter alone is not advised without the re-optimization of all other parameters. Instead, the selection of a functional with a suitable amount of HFx content should be beneficial, which, however, depends on the specific reaction.

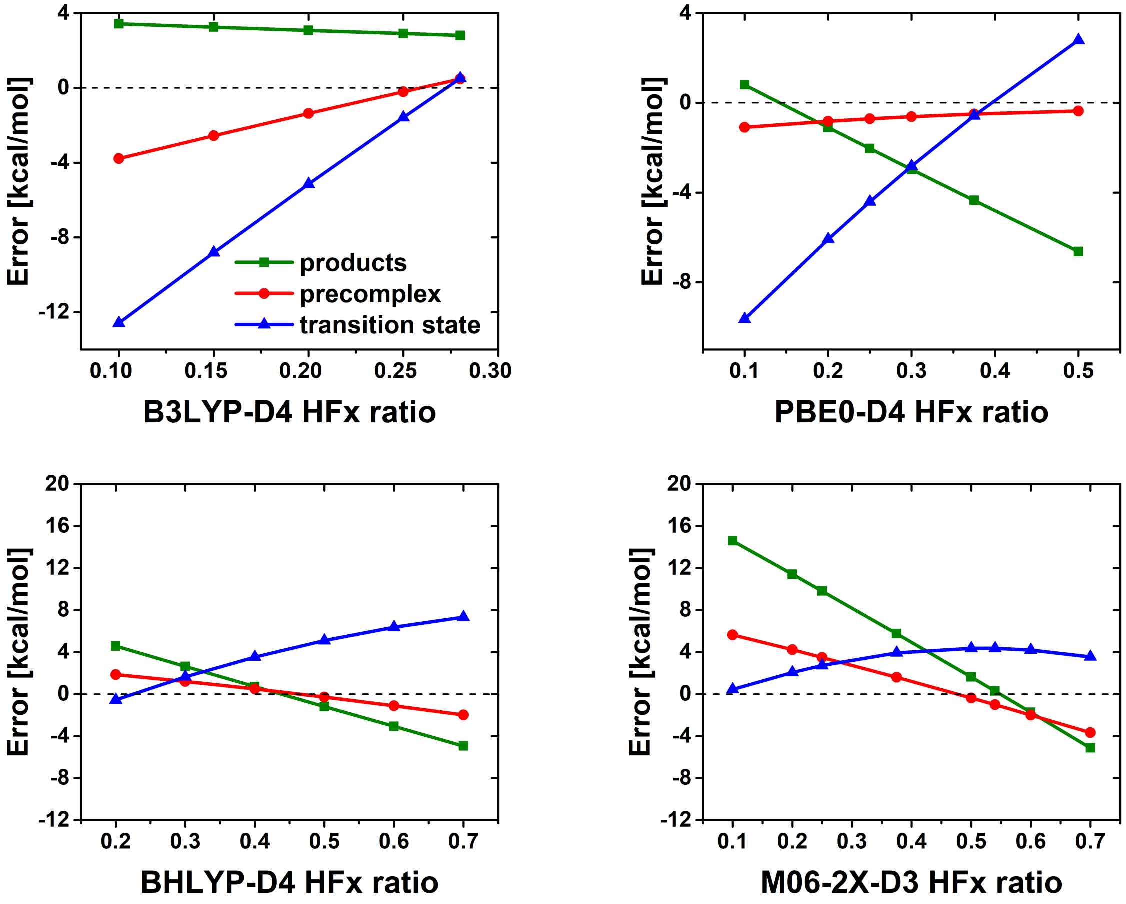

Figure S12: Varying the ratio of exact exchange in several functionals in **reaction B** (halocyclization, Scheme 2). Errors in the stability of the precomplex (**B-prec**), the transition state (**B-ts**) and the products (**2 + MCDHM**) with respect to the separated reactants are given with various ratios of exact exchange.

S2.2.2 The effect of dispersion correction

The errors of all investigated functionals with and without dispersion correction are written in Table S5.

Table S5: Signed error of functionals with and without dispersion correction in **reaction B** (halocyclization, Scheme 2) in kcal/mol. The errors are calculated in the stabilities with respect to the separated reactants.

	With D3/D4/VV10		No D3/D4/VV10	
	Error (**B-prec**)	Error (**B-ts**)	Error (**B-prec**)	Error (**B-ts**)
BP86(-D4)	-2.7	-16.3	11.5	1.4
BLYP(-D4)	-2.3	-13.5	14.2	8.1
PW91(-D3)	-2.0	-14.5	6.4	-4.0
PBE(-D4)	-2.1	-14.5	7.5	-2.4
SCAN(-D4)	-1.4	-14.1	2.1	-9.9
TPSS(-D3)	-1.7	-15.3	10.5	0.4
B97M(-V)	0.9	-4.4	11.7	10.9
M06-L(-D3)	1.1	-3.3	3.6	-0.3
TPSSh(-D4)	-1.2	-11.0	10.6	4.0
B3LYP(-D4)	-1.5	-5.4	12.2	12.1
PBE0(-D4)	-0.9	-4.7	8.2	6.7
MN15(-D3)	1.2	6.4	1.4	6.6
BHLYP(-D4)	-0.3	5.1	10.9	19.1
M06-2X(-D3)	-1.0	4.4	0.9	6.6
ωB97X(-D4)	0.2	7.7	3.1	11.2
ωB97X(-V)	-0.5	3.9	10.4	19.4
ωB97M(-V)	-0.7	2.2	10.5	17.9
LC-ωPBE(-D3)	-0.6	6.3	9.4	18.6
CAM-B3LYP(-D4)	-1.3	0.7	8.3	12.7
B2PLYP(-D4)	-1.3	-5.4	5.8	3.4
DSD-PBEP86(-D4)	-0.6	-2.7	2.1	0.6
revDSD-PBEP86(-D4)	-0.3	-1.6	3.7	3.9
DRPA75(-D3)	-3.0	-4.5	1.5	2.2
DSD-PBEP86-dRPA75(-D4)	-1.4	-0.8	2.2	4.2

The effect of the dispersion correction is schematically represented in Figure S13. For (m)GGAs, the lack of the stabilizing dispersion interaction is compensated by over-stabilization

due to the negative SIE (Figure S13, left side). Then, any attempt to improve upon only one of these errors of opposite sign will increase the total errors (Figure S13, middle). Finally, if both dispersion and SIE are corrected, we obtain more reliable energies for better reasons (Figure S13, right side). On the basis of analogous observations, recent benchmark studies[47–49] on halogen-bonded systems also found that adding dispersion correction increases the error of GGA functionals, which can also be better understood using similar analysis.

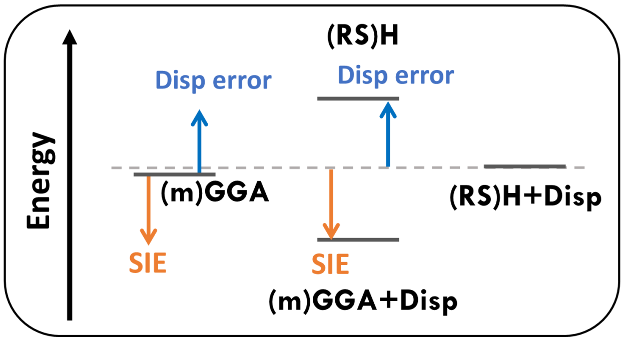

Figure S13: Schematic representation of the cancellation between error components attributed to self-interaction (SIE, orange arrows) and dispersion (Disp, blue arrows).

On Figures S14, S22, and S32 we explore the dependence of the dispersion correction component along the RCs. The Figures show the change in the dispersion correction compared to the first point along the RC, so that the D4 and VV10 corrections can be put on a similar footing. One has to note that, since dispersion is not a physically defined quantity, its characterization varies. Moreover, part of the dispersion is contained in the chosen DFA, especially in short to mid-range, thus the dispersion corrections are not equal to the total dispersion in the system. All in all, it is informative to study the size and RC dependence. The D4 correction to wB97X is found fairly constant along the RC for all cases, indicating consistent behavior upon changes in the structure. In accordance with the formulation of the D3 and D4 schemes, we found similar when they are combined with different DFA. Compared to that the VV10 component of wB97X-V is found also fairly constant along the RC for reactions **B** and **C**. Its variability becomes considerable for reaction **D**, in accordance with the more extensive rearrangement of the large portion of atoms along the RC

(cf. Scheme 4) of the main text). This may indicate that the more elaborate VV10 model could be of preference here, although considering multiple DFA+D3/4 models and the other error sources in Section 3.3 shows that other functional and density based inaccuracies are responsible for the main issues.

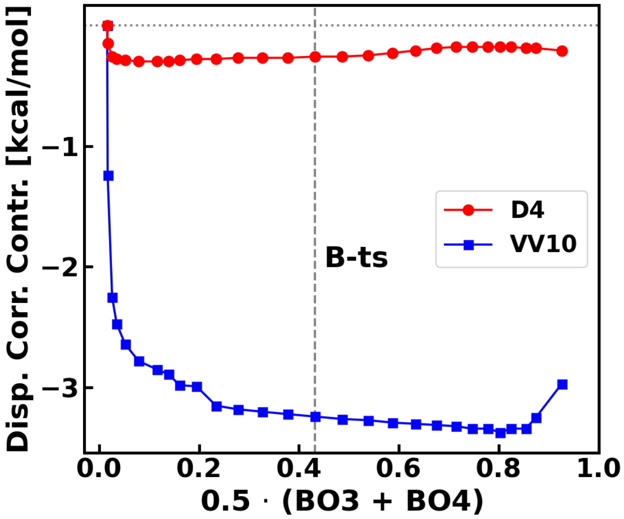

Figure S14: Contribution of different dispersion corrections (D4 and VV10) to the ωB97X functional (resulting in ωB97X-D4 and ωB97X-V, respectively in **reaction B** (halocyclization, Scheme 2). Both D4 and VV10 contributions are given as the increment with respect to their value at the first point of the analysis.

S2.2.3 Analysis along the RC

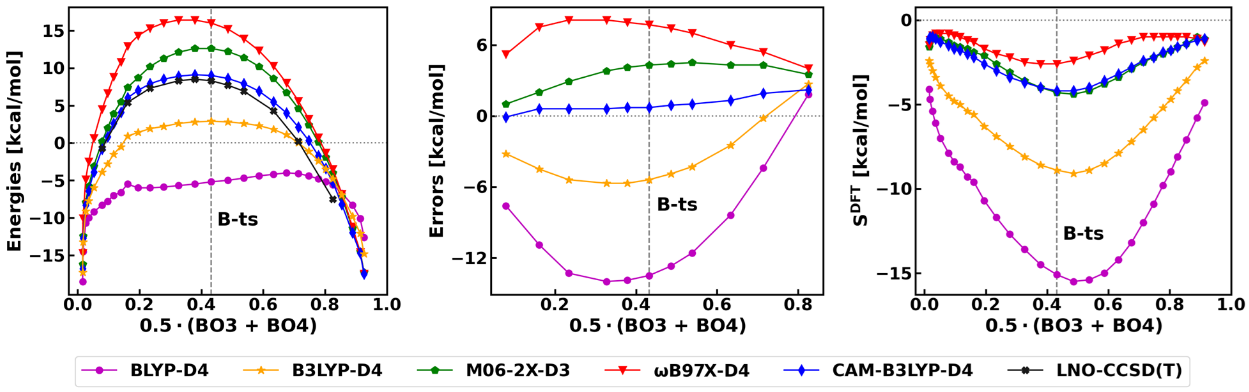

Figure S15: Halocyclization reaction **B**. left: Electronic energies computed with various methods along the reaction coordinate (RC). The RC is defined as the average Mayer Bond Order[46] (BO) of the forming bonds (3 and 4 in Scheme 2). The reactant state (CV $\approx$ 0) corresponds to **B-prec** and the energies are provided with respect to the separated reactants. middle: Signed errors of functionals with respect to LNO-CCSD(T)/CBS results along the RC. right: Density sensitivities of functionals along the RC.

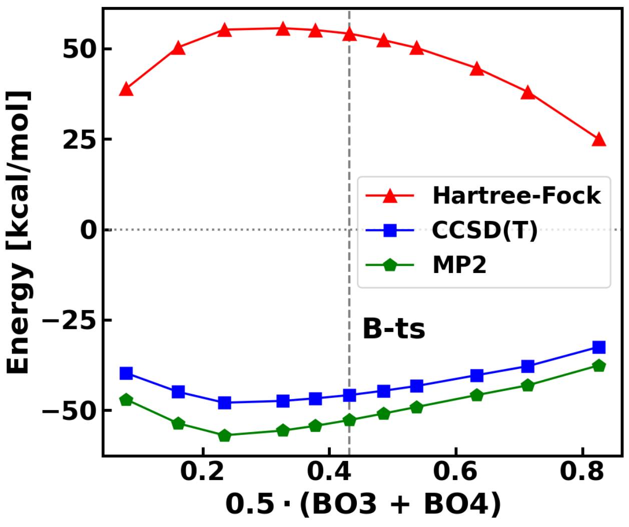

Figure S16: Correlation and Hartree–Fock contributions in **reaction B** (halocyclization, Scheme 2) to the LNO-CCSD(T) energy along the reaction coordinate. The separated reactants are taken as the reference state.

S2.3 Methylation

The errors and density sensitivities with the separated reactants taken as reference state in **C-ts** are plotted in Figure S18 for each group of functional. The data for all structures and the individual functionals with the separated reactants taken as reference are plotted in Figure S17 and are collected in Table S6. The errors and density sensitivities with product state as reference are presented in Figure S19 and Table S7.

The energies, errors and density sensitivities for selected functionals along the RC of the *syn* methylation with the reactant reference are plotted in Figure S21. The HF and correlation contributions along the reaction coordinate are shown in Figure S23.

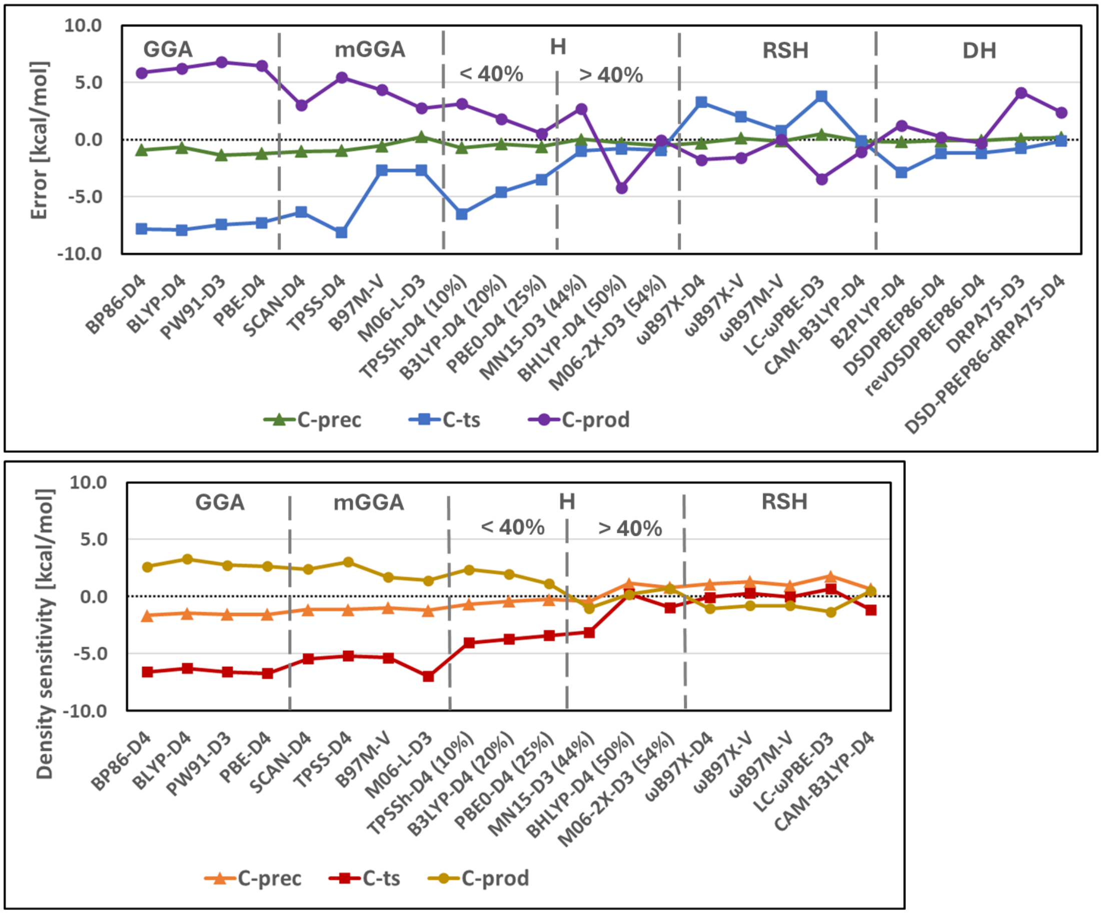

Figure S17: Signed errors (top) and density sensitivities (bottom) in **reaction C** (methylation, Scheme 3). The errors and density sensitivities are calculated in the stabilities **with respect to the separated reactants**.

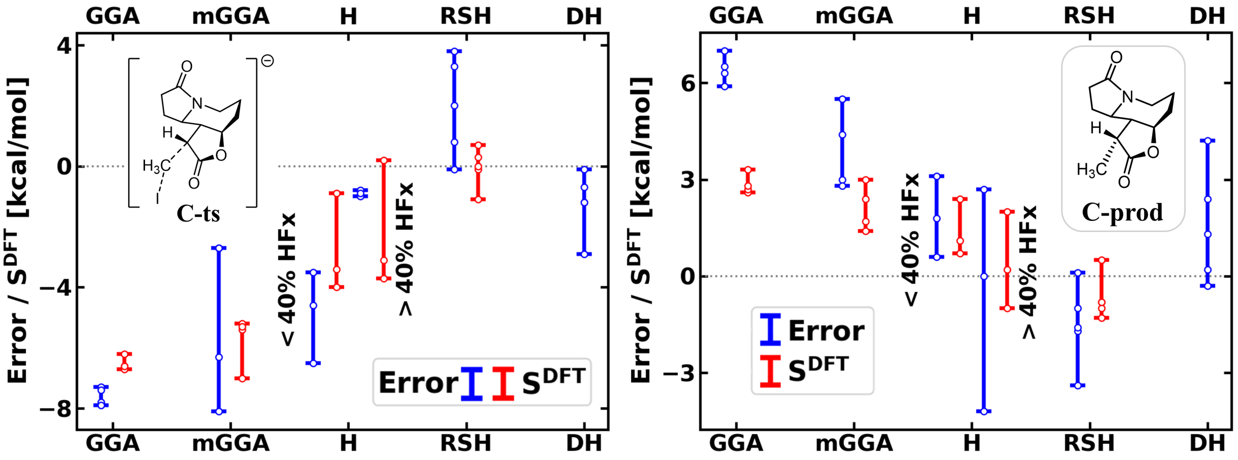

Figure S18: Signed error for **C-ts** (left) and **C-prod** (right) with respect to LNO-CCSD(T) (blue) and density sensitivity (red) of functionals in the methylation reaction (with respect to the separated reactants, Scheme 3). Functional categories are defined in Section 2. See all data in Figure S17 and Table S6.

Table S6: Signed errors, density sensitivities (S^{DFT}) in **reaction C** (methylation, Scheme 3) in kcal/mol. The errors and density sensitivities are calculated in the stabilities **with respect to the separated reactants**.

	Error			S^{DFT}		
	**C-prec**	**C-ts**	**C-prod**	**C-prec**	**C-ts**	**C-prod**
BP86-D4	-0.9	-7.8	5.9	-1.6	-6.6	2.6
BLYP-D4	-0.7	-7.9	6.3	-1.5	-6.2	3.3
PW91-D4	-1.3	-7.4	6.8	-1.6	-6.6	2.8
PBE-D4	-1.2	-7.3	6.5	-1.6	-6.7	2.7
SCAN-D4	-1.0	-6.3	3.0	-1.1	-5.4	2.4
TPSS-D4	-0.9	-8.1	5.5	-1.1	-5.2	3.0
B97M-V	-0.5	-2.7	4.4	-1.0	-5.3	1.7
M06-L-D3	0.3	-2.7	2.8	-1.2	-7.0	1.4
TPSSh-D4 (10%)	-0.7	-6.5	3.1	-0.6	-4.0	2.4
B3LYP-D4 (20%)	-0.4	-4.6	1.8	-0.4	-3.7	2.0
PBE0-D4 (25%)	-0.6	-3.5	0.6	-0.2	-3.4	1.1
MN15-D3 (44%)	0.1	-1.0	2.7	-0.4	-3.1	-1.0
BHLYP-D4 (50%)	-0.3	-0.8	-4.2	1.1	0.2	0.2
M06-2X-D3 (54%)	-0.5	-0.9	0.0	0.8	-0.9	0.7
ωB97X-D4	-0.3	3.3	-1.7	1.1	-0.1	-1.0
ωB97X-V	0.1	2.0	-1.6	1.3	0.3	-0.8
ωB97M-V	-0.1	0.8	0.1	1.0	0.0	-0.8
LC-ωPBE-D3	0.5	3.8	-3.4	1.8	0.7	-1.3
CAM-B3LYP-D4	-0.1	-0.1	-1.0	0.7	-1.1	0.5
B2PLYP-D4	-0.2	-2.9	1.3			
DSD-PBEP86-D4	-0.1	-1.2	0.2			
revDSD-PBEP86-D4	0.0	-1.2	-0.3			
DRPA75-D3	0.1	-0.7	4.2			
DSD-PBEP86-dRPA75-D4	0.2	-0.1	2.4			

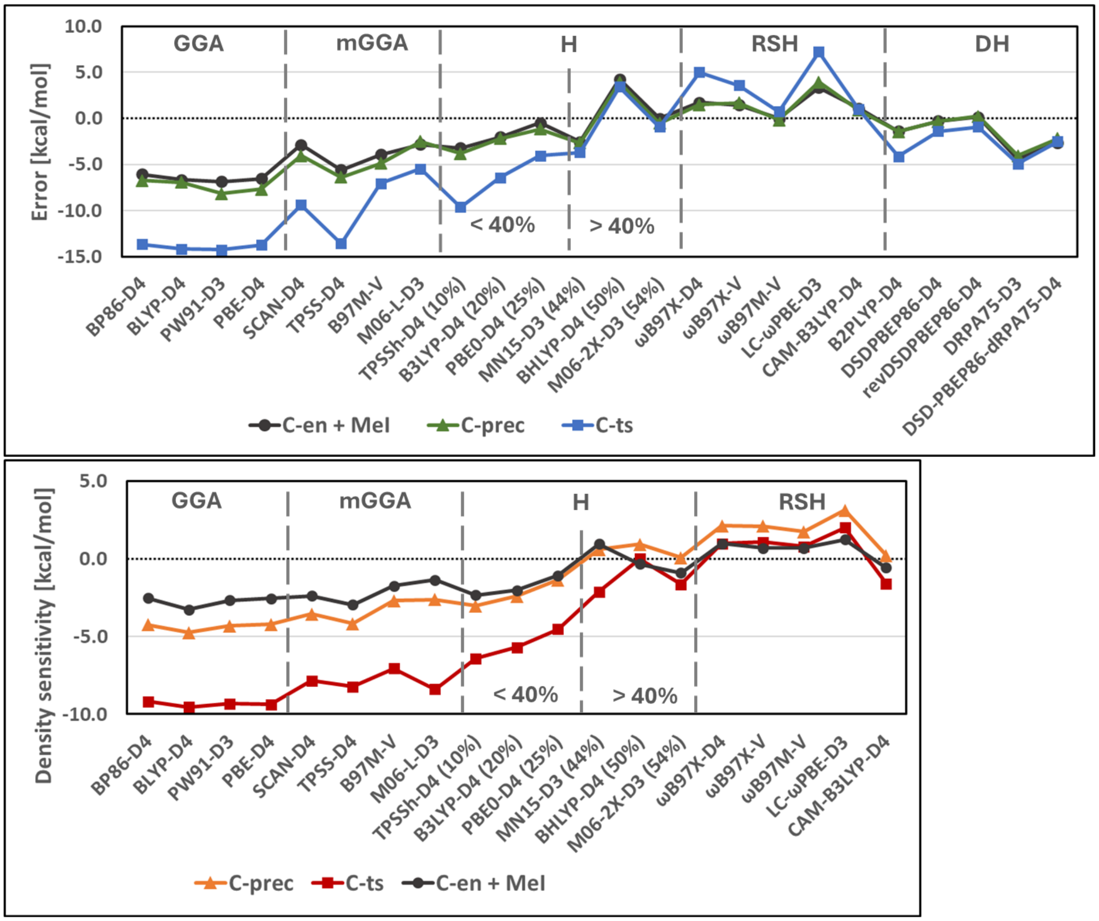

Figure S19: Signed errors (top) and density sensitivities (bottom) in **reaction C** (methylation, Scheme 3). The errors and density sensitivities are calculated in the stabilities **with respect to the separated products**.

Table S7: Signed errors, density sensitivities (S^{DFT}) in **reaction C** (methylation, Scheme 3) in kcal/mol. The errors and density sensitivities are calculated in the stabilities **with respect to the separated products**.

	Error			S^{DFT}		
	**C-en + MeI**	**C-prec**	**C-ts**	**C-en + MeI**	**C-prec**	**C-ts**
BP86-D4	-6.1	-6.7	-13.7	-2.5	-4.2	-9.2
BLYP-D4	-6.7	-6.9	-14.2	-3.3	-4.7	-9.5
PW91-D4	-6.9	-8.1	-14.3	-2.7	-4.3	-9.3
PBE-D4	-6.5	-7.7	-13.7	-2.6	-4.2	-9.4
SCAN-D4	-2.9	-4.0	-9.4	-2.4	-3.5	-7.8
TPSS-D4	-5.6	-6.4	-13.6	-3.0	-4.2	-8.2
B97M-V	-3.9	-4.9	-7.0	-1.7	-2.7	-7.1
M06-L-D3	-2.8	-2.5	-5.5	-1.4	-2.6	-8.4
TPSSh-D4 (10%)	-3.2	-3.8	-9.6	-2.3	-3.0	-6.4
B3LYP-D4 (20%)	-2.0	-2.2	-6.4	-2.0	-2.4	-5.7
PBE0-D4 (25%)	-0.5	-1.2	-4.0	-1.1	-1.3	-4.5
MN15-D3 (44%)	-2.6	-2.7	-3.7	1.0	0.6	-2.1
BHLYP-D4 (50%)	4.3	3.9	3.4	-0.3	0.9	0.0
M06-2X-D3 (54%)	0.0	-0.5	-0.9	-0.9	0.1	-1.6
ωB97X-D4	1.7	1.5	5.0	1.0	2.1	1.0
ωB97X-V	1.4	1.7	3.6	0.7	2.1	1.1
ωB97M-V	0.0	-0.2	0.7	0.7	1.7	0.8
LC-ωPBE-D3	3.4	3.9	7.2	1.2	3.1	2.0
CAM-B3LYP-D4	1.1	0.9	1.0	-0.6	0.2	-1.6
B2PLYP-D4	-1.4	-1.5	-4.1			
DSD-PBEP86-D4	-0.3	-0.3	-1.4			
revDSD-PBEP86-D4	0.1	0.2	-0.9			
DRPA75-D3	-4.4	-4.1	-4.9			
DSD-PBEP86-dRPA75-D4	-2.6	-2.2	-2.5			

Compared to the analysis with respect to the product state in the main text (Figure S20), the corresponding Figure S21 choosing the reactant state as reference could look more confusing at first sight. However, it can be similarly understood considering that it shows the same trends as Figure 5 with a constant shift due to the change of reference. This further reinforces the conclusion in the main text, for example about the lower-than-expected forward barrier height errors. Namely, that can be understood as the combination of a large negative SIE in the 3c/4e TS and a positive error component due to the overpolarized iodine already at the reactant state.

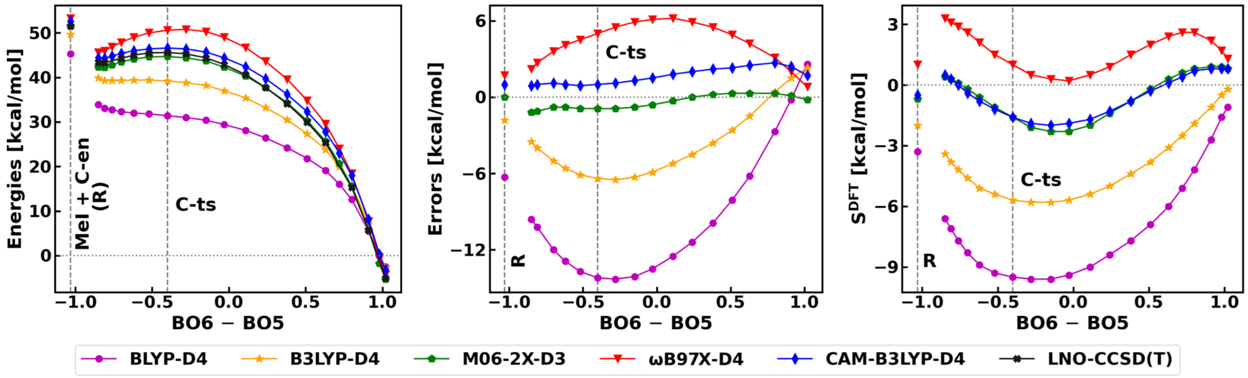

Figure S20: Reverse of the methylation reaction. left: Electronic energies along the RC with respect to the separated products. The RC is defined as the difference between the BOs of the formed and cleaved bonds (6 and 5 in Scheme 3). middle: Signed DFT errors with respect to LNO-CCSD(T)/CBS along the RC. right: Density sensitivities along the RC. **R** labels the state of infinitely separated reactants.

To further investigate the trends for the functionals where the above SIE sources do not clearly dominate, let us inspect in Figure S23 the HF and correlation energy components along the RC. Here, the CCSD(T) correlation energy contributions of about 10–20 kcal/mol are notable, but somewhat lower than those obtained for the halocyclization. However, the post-MP2 contribution is very small, indicating simpler correlation effects. As one may expect from the significant rearrangement of explicit charges during the reaction, the electrostatic effects present already in HF are the most extensive components of energy stabilization.

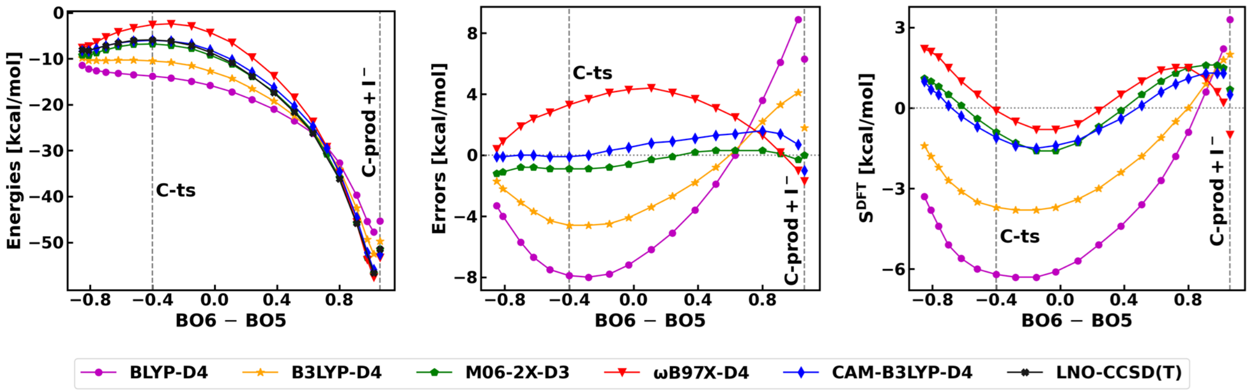

Figure S21: left: Electronic energies with various methods along the RC of the methylation reaction with the separated reactant state taken as the reference. The RC is defined as the difference between the BOs of the formed and cleaved bonds (6 and 5 in Scheme 3). middle: Signed errors of functionals with respect to LNO-CCSD(T)/CBS results along the RC. right: Density sensitivities of functionals along the RC.

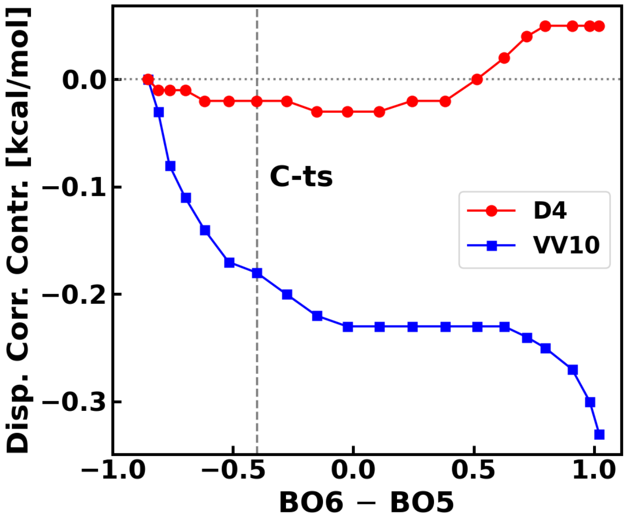

Figure S22: Contribution of different dispersion corrections (D4 and VV10) to the ωB97X functional (resulting in ωB97X-D4 and ωB97X-V, respectively in **reaction C** (methylation, Scheme 3). Both D4 and VV10 contributions are given as the increment with respect to their value at the first point of the analysis.

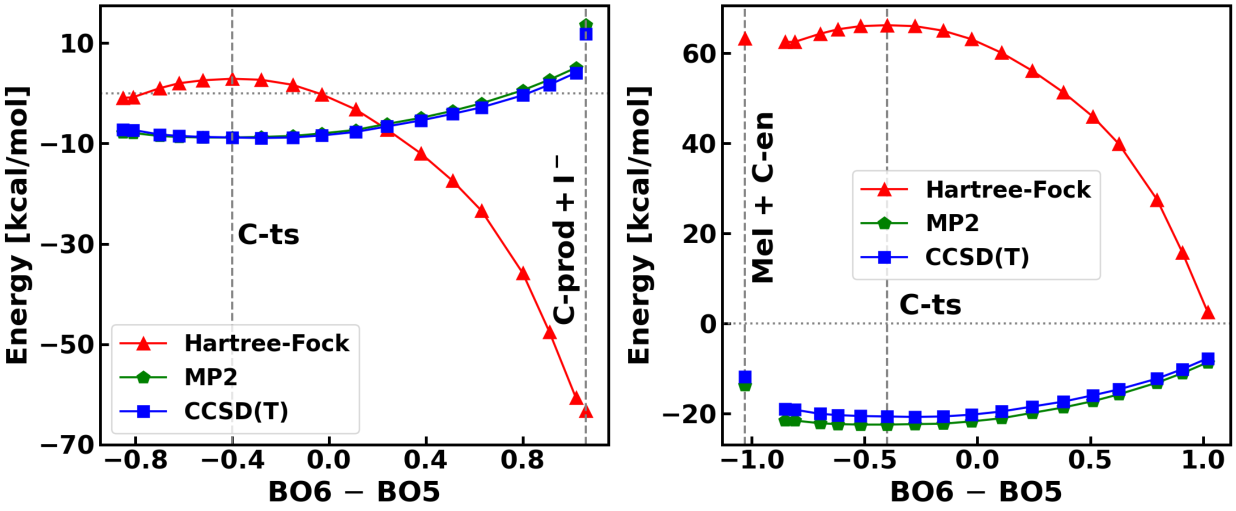

Figure S23: Correlation and Hartree–Fock contributions in **reaction C** (methylation, Scheme 3) to the LNO-CCSD(T) energy along the reaction coordinate. The separated reactants are taken as the reference state on the left figure and the separated products on the right.

S2.3.1 Anti methylation

The *syn* and the *anti* methylation are schematically represented in Figure S24. In the article, only the more favored *syn* pathway is discussed, so for simplicity, the *syn* structures are denoted as **C-prec**, **C-ts** and **C-prod**, while the notations of the *anti* structures include the **anti** prefix. First, we present additional data to the *syn* methylation and then we introduce the results on the *anti* pathway.

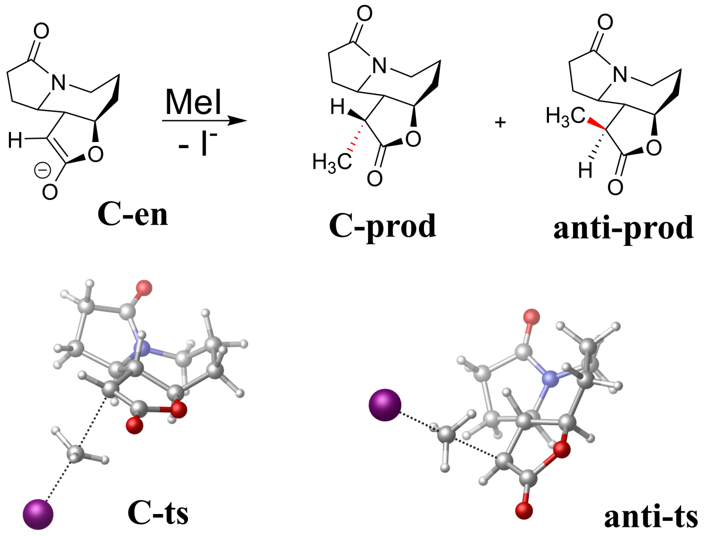

Figure S24: Details of the methylation reaction (**reaction C**, Scheme 3). In the article, the *syn* methylation (**C-ts** and **C-prod**) are investigated, and the results of the less favored *anti* path (**anti-ts** and **anti-prod**) are introduced in Section S2.3.1.

The errors and density sensitivities of functionals in the electronic energies with respect to the reactants in the *anti* methylation can be seen in Figure S25 and Table S8. The tendencies are completely analogous to those discussed for the syn methylation.

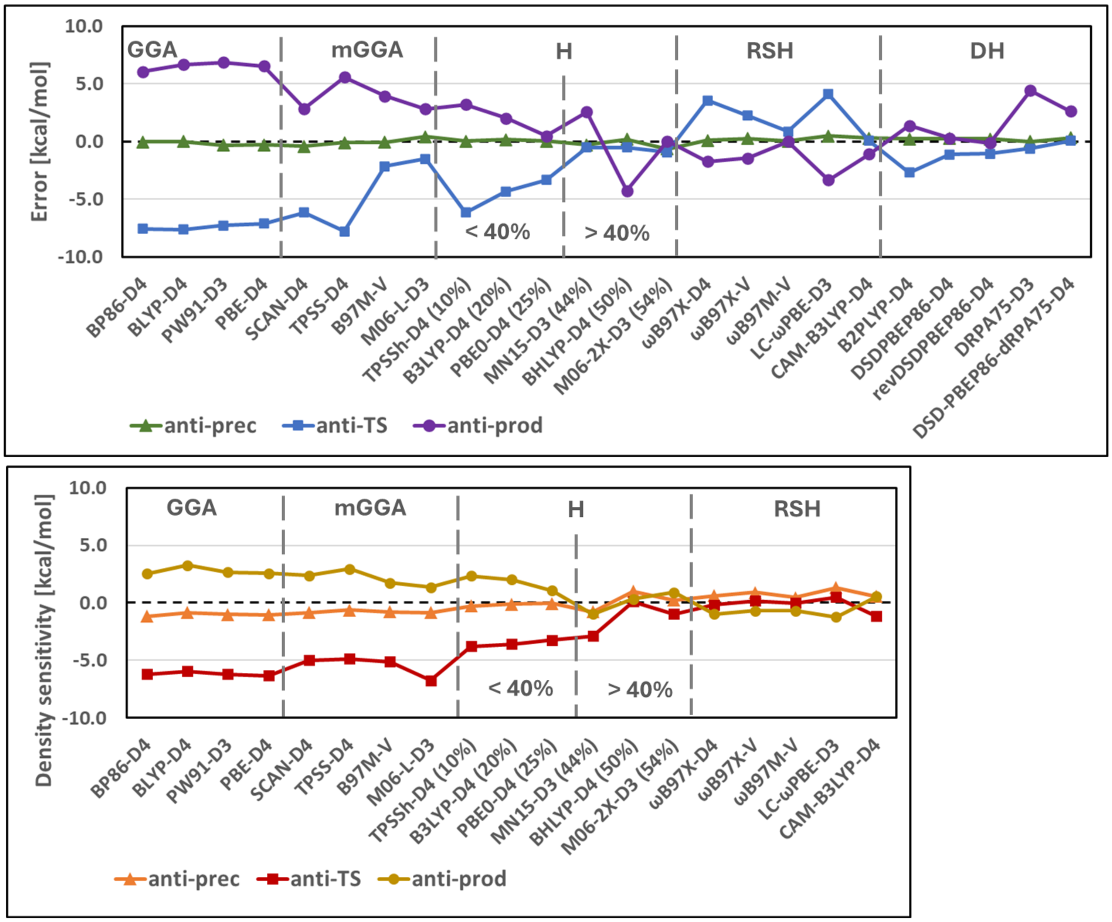

Figure S25: Signed errors (top) and density sensitivities (bottom) in the **anti methylation** (Figure S24). The errors and density sensitivities are calculated in the stabilities **with respect to the separated products**.

Table S8: Signed errors, density sensitivities (S^{DFT}) in the **anti methylation** (Figure S24) in kcal/mol. The errors and density sensitivities are calculated in the stabilities **with respect to the separated products**.

	Error			S^{DFT}		
	**anti-prec**	**anti-TS**	**anti-prod**	**anti-prec**	**anti-TS**	**anti-prod**
BP86-D4	0.0	-7.6	6.1	-1.1	-6.2	2.5
BLYP-D4	0.0	-7.7	6.7	-0.9	-6.0	3.3
PW91-D4	-0.3	-7.3	6.9	-1.0	-6.2	2.7
PBE-D4	-0.3	-7.1	6.5	-1.0	-6.3	2.6
SCAN-D4	-0.4	-6.2	2.9	-0.8	-5.0	2.4
TPSS-D4	-0.1	-7.8	5.6	-0.6	-4.9	3.0
B97M-V	-0.1	-2.2	3.9	-0.8	-5.1	1.7
M06-L-D3	0.4	-1.5	2.8	-0.9	-6.7	1.4
TPSSh-D4 (10%)	0.0	-6.1	3.2	-0.3	-3.8	2.3
B3LYP-D4 (20%)	0.2	-4.3	2.0	-0.1	-3.6	2.0
PBE0-D4 (25%)	0.0	-3.3	0.5	-0.1	-3.2	1.1
MN15-D3 (44%)	-0.3	-0.5	2.6	-0.8	-2.9	-1.0
BHLYP-D4 (50%)	0.2	-0.5	-4.3	1.0	0.1	0.3
M06-2X-D3 (54%)	-0.6	-0.9	0.0	0.2	-1.0	0.9
ωB97X-D4	0.1	3.6	-1.7	0.6	-0.2	-1.0
ωB97X-V	0.3	2.2	-1.4	0.9	0.2	-0.7
ωB97M-V	0.0	0.9	0.0	0.5	0.0	-0.7
LC-ωPBE-D3	0.5	4.1	-3.4	1.3	0.5	-1.2
CAM-B3LYP-D4	0.3	0.1	-1.1	0.6	-1.2	0.6
B2PLYP-D4	0.2	-2.7	1.4			
DSD-PBEP86-D4	0.3	-1.1	0.3			
revDSD-PBEP86-D4	0.2	-1.1	-0.1			
DRPA75-D3	0.0	-0.6	4.4			
DSD-PBEP86-dRPA75-D4	0.3	0.1	2.6			

S2.3.2 Syn VS anti methylation

As we noted in the article, the errors of functionals in terms of the difference between the barriers of the *syn* and *anti* methylation (forward reaction) are relatively small as a consequence of the analogous errors along the two reactions. The error of all investigated functionals in the difference between the barrier of the (forward) *syn* and the *anti* methylation is presented in Table S9.

Table S9: Error of functionals in the difference between the barrier (with respect to the separated reactants) *syn* and the *anti* methylation in kcal/mol

	Error (**syn-TS**) − Error (**anti-TS**)
BP86-D4	-0.2
BLYP-D4	-0.3
PW91-D3	-0.1
PBE-D4	-0.2
SCAN-D4	-0.2
TPSS-D4	-0.3
B97M-V	-0.5
M06-L-D3	-1.2
TPSSh-D4	-0.3
B3LYP-D4	-0.2
PBE0-D4	-0.1
MN15-D3	-0.4
BHLYP-D4	-0.3
M06-2X-D3	0.0
ωB97X-D4	-0.3
ωB97X-V	-0.2
ωB97M-V	-0.1
LC-ωPBE-D3	-0.3
CAM-B3LYP-D4	-0.2
B2PLYP-D4	-0.1
DSD-PBEP86-D4	-0.1
revDSD-PBEP86-D4	-0.1
DRPA75-D3	-0.1
DSD-PBEP86-dRPA75-D4	-0.2

S2.4 Michael addition

The errors and density sensitivities represented with error bars for each functional group are shown in Figure S26. The error and density sensitivity of each functional can be seen in Figure S27 and Table S10. The correlation energies along the reaction coordinate are plotted in Figure S33.

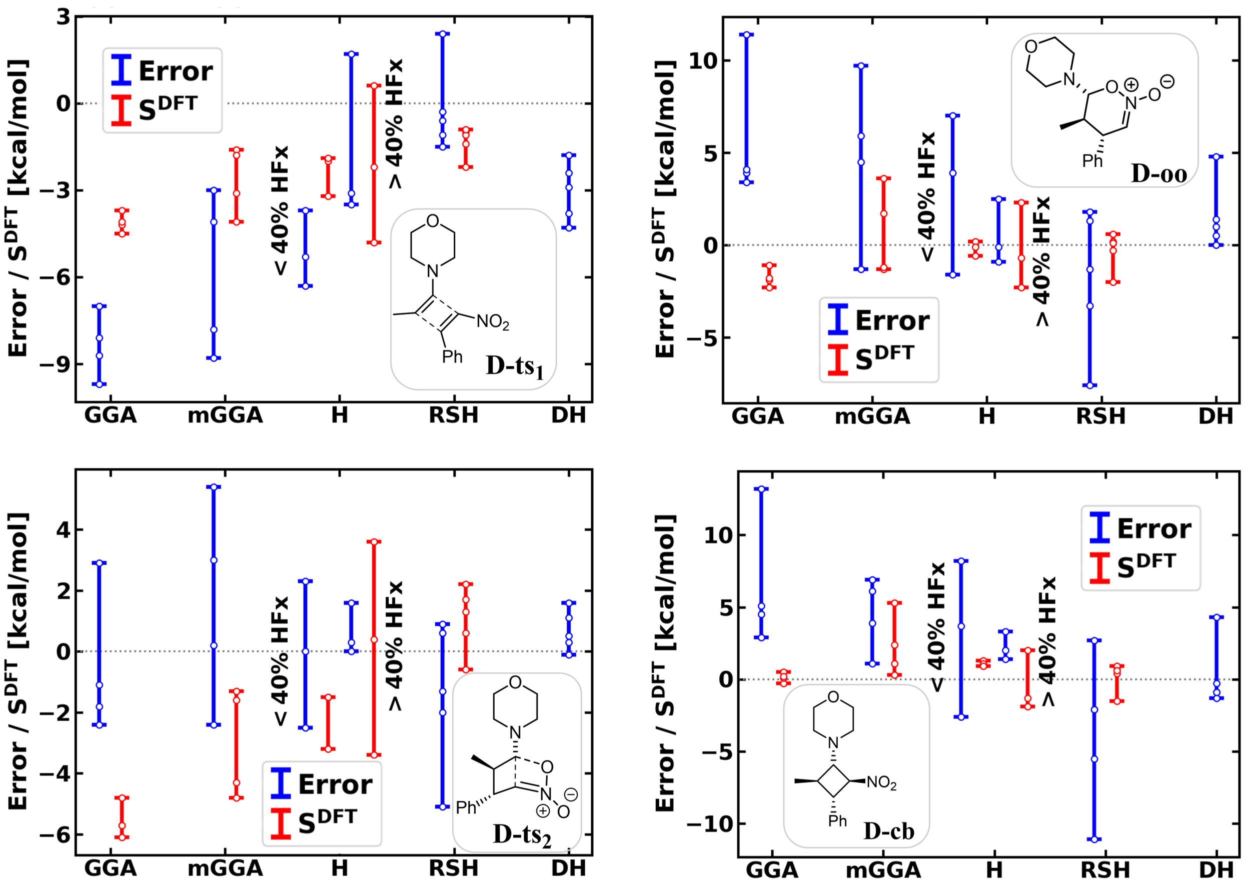

Figure S26: Signed error with respect to LNO-CCSD(T) (left bars, blue) and density sensitivity (right bars, red) of functionals in the energy of the transition states and intermediates in the Michael addition (with respect to separated reactants, see Scheme 4). Functional categories are defined in Section 2. See all data in Figure S27 or Table S10.

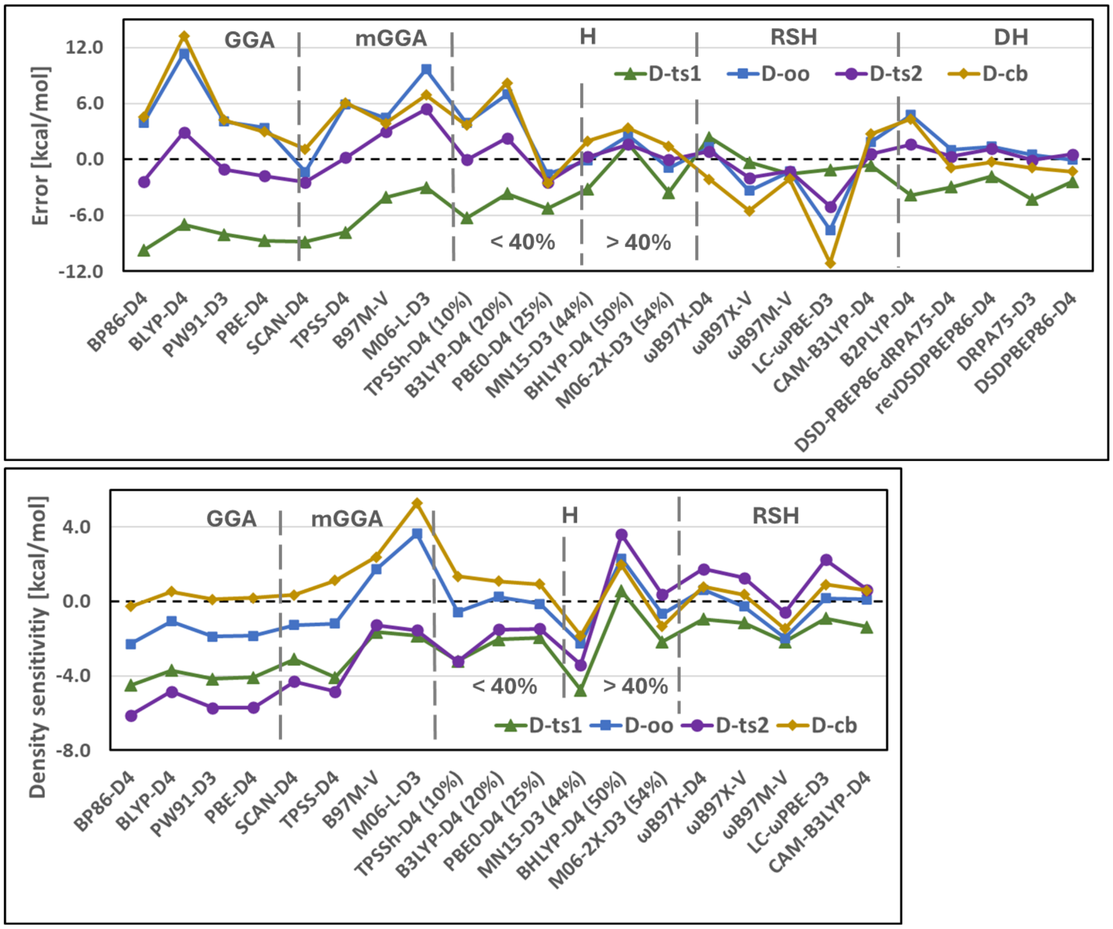

Figure S27: Signed errors (top) and density sensitivities (bottom) in **reaction D** (Michael addition, Scheme 4). The errors and density sensitivities are calculated in the stabilities with respect to the separated reactants.

Table S10: Signed errors, density sensitivities (S^{DFT}) in **reaction D** (Michael addition, Scheme 4) in kcal/mol. The errors and density sensitivities are calculated in the stabilities with respect to the separated reactants.

	Error				S^{DFT}			
	**D-ts$_1$**	**D-oo**	**D-ts$_2$**	**D-cb**	**D-ts$_1$**	**D-oo**	**D-ts$_2$**	**D-cb**
BP86-D4	-9.7	4.0	-2.2	4.6	-4.5	-2.3	-6.1	-0.3
BLYP-D4	-6.9	11.5	3.1	13.3	-3.7	-1.1	-4.8	0.5
PW91-D3	-8.0	4.2	-0.9	4.3	-4.2	-1.9	-5.7	0.1
PBE-D4	-8.7	3.5	-1.5	3.0	-4.1	-1.8	-5.7	0.2
SCAN-D4	-8.8	-1.2	-2.2	1.2	-3.1	-1.3	-4.3	0.3
TPSS-D4	-7.7	6.1	0.4	6.2	-4.1	-1.2	-4.8	1.1
B97M-V	-4.0	4.6	3.2	4.0	-1.6	1.7	-1.3	2.4
M06-L-D3	-2.9	9.8	5.6	7.0	-1.8	3.6	-1.6	5.3
TPSSh-D4 (10%)	-6.2	4.0	0.2	3.8	-3.2	-0.6	-3.2	1.3
B3LYP-D4 (20%)	-3.6	7.1	2.5	8.3	-2.0	0.2	-1.5	1.1
PBE0-D4 (25%)	-5.2	-1.5	-2.2	-2.5	-1.9	-0.1	-1.5	0.9
MN15-D3 (44%)	-3.1	0.0	0.5	2.1	-4.8	-2.3	-3.4	-1.9
BHLYP-D4 (50%)	1.8	2.6	1.8	3.4	0.6	2.3	3.6	2.0
M06-2X-D3 (54%)	-3.5	-0.8	0.2	1.5	-2.2	-0.7	0.4	-1.3
ωB97X-D4	2.4	1.4	1.1	-2.0	-1.0	0.6	1.7	0.8
ωB97X-V	-0.3	-3.2	-1.8	-5.4	-1.1	-0.3	1.3	0.4
ωB97M-V	-1.5	-1.2	-1.1	-2.0	-2.2	-2.0	-0.6	-1.5
LC-ωPBE-D3	-1.0	-7.5	-4.9	-11.0	-0.9	0.2	2.2	0.9
CAM-B3LYP-D4	-0.6	2.0	0.8	2.8	-1.4	0.1	0.6	0.6
B2PLYP-D4	-3.8	4.9	1.8	4.4				
DSD-PBEP86-dRPA75-D4	-2.9	1.1	0.5	-0.8				
revDSD-PBEP86-D4	-1.7	1.5	1.4	-0.2				
DRPA75-D3	-4.2	0.6	0.1	-0.8				
DSD-PBEP86-D4	-2.3	0.1	0.8	-1.2				

Some important geometries from the investigated region of the RC are depicted in Figure S28. These are the one closest to the reactant state, the transition state **D-ts$_\mathbf{1}$**, the point where bond 8 is approximately halfway formed (second minima of S^{DFT} curves), and the geometry closest to **D-oo**. In the second reaction, bond 8 is cleaved and then a new C-C bond (9) is formed, so the difference between these BOs gives the RC. The geometry close to the first minima of the density sensitivities, **D-ts$_\mathbf{2}$**, a point close to the second minima of density sensitivities, and the geometry closest to **D-cb** are depicted in S29. The density sensitivities again show minima close to the points where one of the bonds is halfway formed (cleaved).

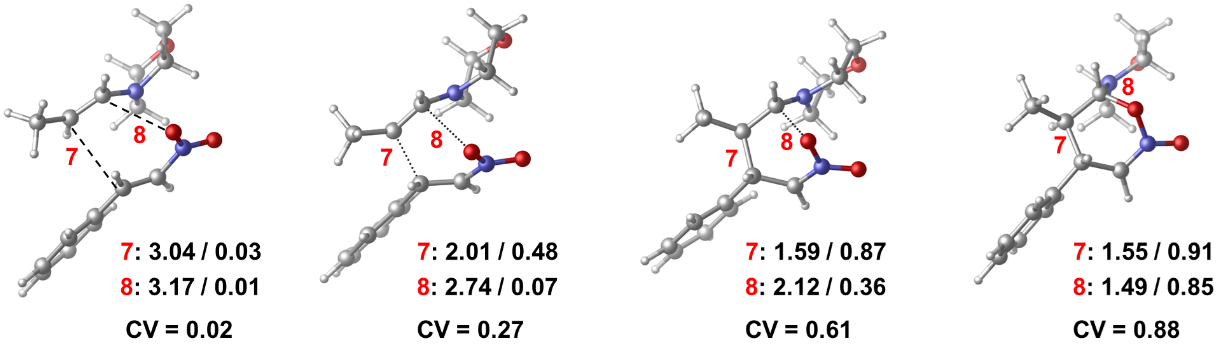

Figure S28: Important geometries along the RC of the first elementary step of the Michael addition. Bond distance in angstrom / bond order is given for selected atom pairs, and the collective variable (CV, chosen as RC) is calculated as the average of these bond orders.

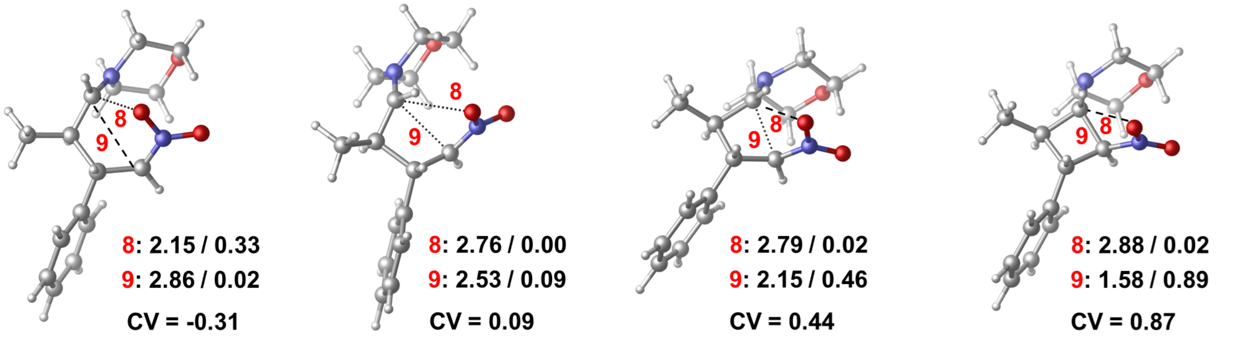

Figure S29: Important geometries along the RC of the second elementary step of the Michael addition. Bond distance in angstrom / bond order is given for selected atom pairs, and the collective variable (CV, chosen as RC) is calculated as BO9−BO8.

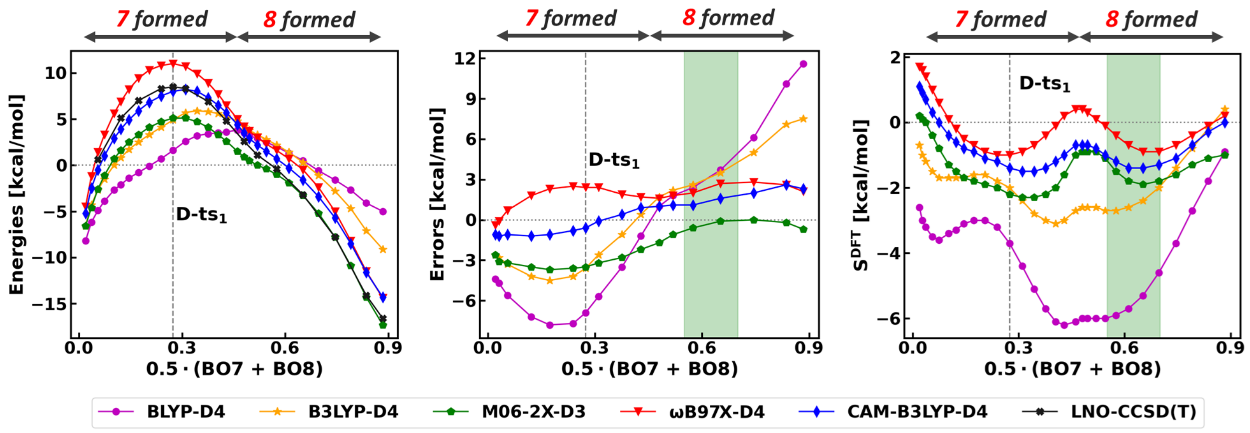

Figure S30: First step of the Michael addition. left: Electronic energies along the RC with respect to the separated reactants. The RC is defined as the average BO of the forming bonds (7 and 8 in Scheme 4). middle: Signed errors of functionals with respect to LNO-CCSD(T)/CBS results along the RC. right: Density sensitivities of functionals along the RC. The structure representative of the green highlighted region is depicted in Figure S28.

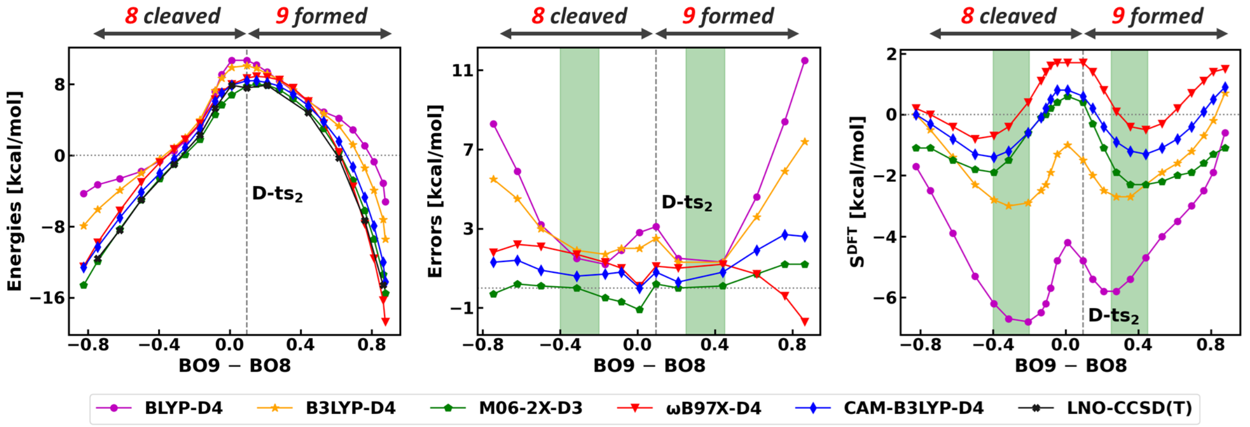

Figure S31: Second step of the Michael addition. left: Electronic energies along the RC with respect to the separated reactants. The RC is defined as the difference between the BOs of the formed and cleaved bonds (9 and 8 in Scheme 4). middle: Signed errors of functionals with respect to LNO-CCSD(T)/CBS results along the RC. right: Density sensitivities of functionals along the RC. The structures representative of the green highlighted regions are depicted in Figure S29.

The Hartree–Fock and correlation energy contributions along the RC are presented in Figure S33. The CCSD(T) correlation energy is significant in the entire reaction. The MP2 correlation is always more negative, but the difference is generally between 1 and 2 kcal/mol, except around **D-ts$_1$**, where MP2 overestimates the stability by almost 5 kcal/mol.

These findings explain why double hybrid functionals are accurate in the intermediates and the second transition state, but give negative errors in **D-ts$_1$** (Figure 6 and Table S10). Interestingly, compared to the more flat correlation contribution, the HF term shows a notable drop starting around **D-ts$_1$**. That drop is similar in size and shape (with opposite sign) to the error pattern emerging and increasing from **D-ts$_1$**, that we assign to functional errors.

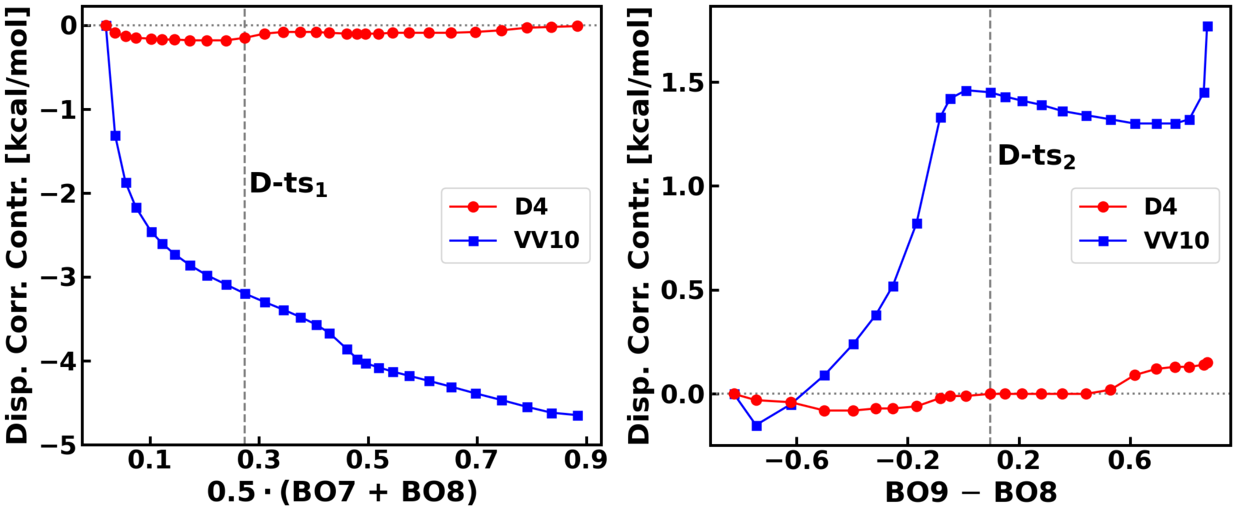

Figure S32: Contribution of different dispersion corrections (D4 and VV10) to the ωB97X functional (resulting in ωB97X-D4 and ωB97X-V, respectively in **reaction D** (Michael addition, Scheme 4). Both D4 and VV10 contributions are given as the increment with respect to their values at the first points of the reaction coordinates.

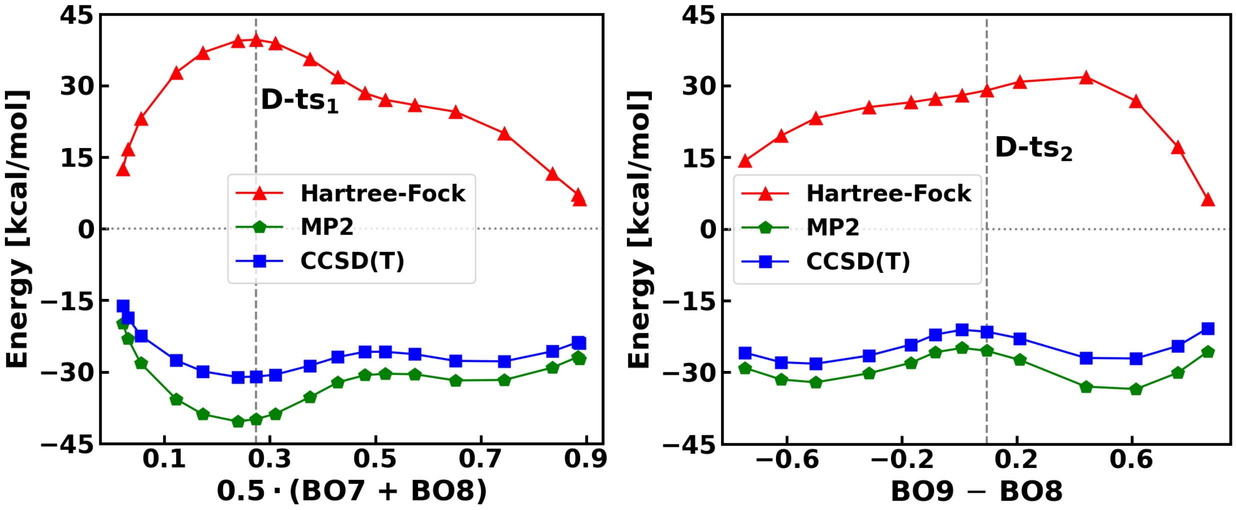

Figure S33: Correlation and Hartree–Fock contributions in **reaction D** (Michael addition, Scheme 4) to the LNO-CCSD(T) energy along the reaction coordinate. The separated reactants are taken as the reference state.

S3 Statistics

Table S11: Correlation coefficient between the errors and the density sensitivities in various structures. **C-ts'** denotes the barrier height in the *syn* methylation with respect to the products.

**A-ts**	**B-ts**	**C-ts**	**C-prod**	**C-ts'**	**C-en + MeI**	**D-ts$_1$**	**D-oo**	**D-ts$_2$**	**D-cb**
0.94	0.92	0.87	0.82	0.96	0.82	0.83	0.21	0.04	0.24

Table S12: Mean absolute deviations (MAD), lowest (most negative, MIN) and largest (most positive, MAX) error in each functional category.

		**A-ts**	**B-ts**	**C-ts**	**D-ts$_1$**	**D-oo**	**D-ts$_2$**	**D-cb**
**GGA**	MAD	11.3	14.4	7.6	8.3	5.8	1.9	6.3
	range	[-12.0,-10.9]	[-16.3,-13.2]	[-7.9,-7.3]	[-9.7,-6.9]	[3.5,11.5]	[-2.2,3.1]	[3.0,13.3]
	MAX−MIN	1.1	3.1	0.6	3.1	8.0	5.3	10.3
**mGGA**	MAD	8.8	9.3	5.0	5.9	5.4	2.9	4.6
	range	[-11.7,-5.8]	[-15.3,-3.3]	[-8.1,-2.7]	[-8.8,-2.9]	[-1.2,9.8]	[-2.2,5.6]	[1.2,7.0]
	MAX−MIN	5.9	12.0	5.5	5.8	11.0	7.8	5.8
**H (<40%)**	MAD	7.6	7.0	4.8	5.0	4.2	1.6	4.8
	range	[-9.5,-5.5]	[-11.0,-4.7]	[-6.5,-3.5]	[-6.2,-3.6]	[-1.5,7.1]	[-2.2,2.5]	[-2.5,8.3]
	MAX−MIN	3.9	6.3	3.0	2.6	8.6	4.7	10.8
**H (<40%)**	MAD	3.8	5.3	0.9	2.8	1.1	0.8	2.3
	range	[-4.9,-3.2]	[4.4,6.4]	[-1.0,-0.8]	[-3.5,-1.8]	[-0.8,2.6]	[0.2,1.8]	[1.5,3.4]
	MAX−MIN	1.7	2.0	0.2	5.2	3.4	1.7	1.9
**RSH**	MAD	2.5	4.2	2.0	1.2	3.0	1.9	4.7
	range	[-4.0,1.6]	[0.7,7.7]	[-0.1,3.8]	[-1.5,2.4]	[-7.5,2.0]	[-4.9,1.1]	[-11.0,2.8]
	MAX−MIN	5.7	7.0	3.9	3.9	9.4	5.9	13.8
**DH**	MAD	4.0	3.0	1.2	3.0	1.7	0.9	1.5
	range	[-5.4,-3.4]	[-5.4,-0.8]	[-2.9,-0.1]	[-4.2,-1.7]	[0.1,4.9]	[0.1,1.8]	[-1.2,4.4]
	MAX−MIN	2.1	4.6	2.7	2.5	4.8	1.7	5.7

Table S13: Signed errors and mean absolute error (MAE) for the best functional in each category.

		A-ts	B-ts	C-ts	D-ts$_1$	D-oo	D-ts$_2$	D-cb	MAE
GGA	PBE-D4	-10.9	-13.2	-7.3	-8.7	3.4	-1.8	2.9	6.9
mGGA	B97M-V	-6.7	-4.4	-2.7	-4.1	4.5	3.0	3.9	4.2
H (<40%)	PBE0-D4 (25%)	-5.5	-4.7	-3.5	-5.3	-1.6	-2.5	-2.6	3.7
H (>40%)	M06-2X-D3 (54%)	-3.2	4.4	-0.9	-3.5	-0.9	0.0	1.4	2.1
RSH	CAM-B3LYP-D4	-4.0	0.7	-0.1	-0.6	1.8	0.6	2.7	1.5
DH	DSD-PBEP86-dRPA75-D4	-3.4	-0.8	-0.1	-2.4	1.0	0.3	-0.9	1.3

S4 Sample input files

An example input of a DFT calculation in Mrcc reads as:

```
calc=wB97X-D3
basis=def2-TZVPP
mem=20gb
popul=mulli
charge=-1
geom=xyz
6
A-ts
C    -0.191614   0.652376  -0.514821
H    -1.271584   0.652435  -0.511585
H     0.348593   1.587496  -0.541447
H     0.348595  -0.282836  -0.491429
Cl   -0.189014   0.714898   1.787950
Cl   -0.202857   0.590103  -2.817571
```

where the Mulliken population analysis provides, among others, the MBOs. In order to calculate the density sensitivity, a Hartree–Fock and an LDA energy calculations are run with the `calc=DF-HF` and the

```
calc=df-scf
dft=user
2
1.0 LDA
1.0 VWN3
```

keywords, respectively. The energy computed with the frozen HF and LDA densities for each functional then can be obtained by reading the HF and LDA densities and performing a single Kohn–Sham iteration, e.g.:

```
calc=wB97X-D3
scfiguess=restart
scfmaxit=1
```

To operate the `scfiguess=restart` restart functionality, the `VARS` and `SCFDENSITIES` files generated by the corresponding HF and LDA calculations have to be copied into the working directory of the DFT computation.
A sample input file for the LNO-CCSD(T) calculations reads as:

```
calc=LNO-CCSD(T)
lcorthr=tight
basis=aug-cc-pVTZ
mem=25gb
```

```
geom=xyz
18
D-ns
C    0.617158     0.445177    -0.000050
C    1.593190    -0.456797     0.000063
N    2.986628    -0.030931     0.000020
O    3.245625     1.156987    -0.000132
O    3.811120    -0.926209     0.000064
C   -0.820639     0.174267    -0.000021
C   -1.352002    -1.121053     0.000047
C   -2.722467    -1.317758     0.000076
C   -3.587402    -0.226768     0.000036
C   -3.073707     1.062969    -0.000036
C   -1.699929     1.260568    -0.000066
H    0.924684     1.487138    -0.000170
H   -0.693560    -1.981981     0.000072
H   -3.121005    -2.325513     0.000128
H   -4.659605    -0.385229     0.000059
H   -3.741698     1.916165    -0.000069
H   -1.298793     2.268533    -0.000123
H    1.504411    -1.532443     0.000191
```

For completeness, we also provide an LNO-CCSD(T) with an extended basis set definition covering all elements and basis sets used in this study:

```
calc=LNO-CCSD(T)
localcc=2021
lcorthr=tight
mem=25gb

basis=atomtype
I:aug-cc-pwCVQZ-PP
O:aug-cc-pVQZ
C:aug-cc-pVQZ
H:aug-cc-pVQZ
N:aug-cc-pVQZ
Cl:aug-cc-pV(Q+d)Z

dfbasis_scf=atomtype
I:aug-cc-pwCVQZ-PP-gen
O:aug-cc-pVQZ-RI-JK
C:aug-cc-pVQZ-RI-JK
H:aug-cc-pVQZ-RI-JK
N:aug-cc-pVQZ-RI-JK
Cl:aug-cc-pVQZ-RI-JK
```

```
dfbasis_cor=atomtype
I:aug-cc-pwCVQZ-PP-gen
O:aug-cc-pVQZ-RI
C:aug-cc-pVQZ-RI
H:aug-cc-pVQZ-RI
N:aug-cc-pVQZ-RI
Cl:aug-cc-pVQZ-RI

ecp=atomtype
I:MCDHF-ECP-28
core=5
charge=0

geom=xyz
5
MeI
C -1.24571600 0.09054800 -0.01360000
H -0.86954000 -0.93784300 0.00686200
H -0.86951200 0.62251900 -0.89393600
H -2.34066500 0.10249000 0.00700500
I -0.53201500 1.09995400 1.73466100
```

For the density fitting basis set of iodine, we employed large decontracted basis sets generated following Ref. 50, which we provide in the supplementary files as a GENBAS MRCC basis set file.

The D3 correction computations are automated in MRCC, while Grimme's dftd4 program can be run for the D4 corrections as follows: `dftd4 --func DFTname STRUCTUREfile` . The D4 corrections to DSD-PBEP86-dRPA75 are obtained as:

`dftd4 --param  0.4257 0.0000 -0.1455 6.3983 --mbdscale 0.6342`.

References

(1) Dunning, Jr., T. H. *J. Chem. Phys.* **1989**, *90*, 1007.

(2) Peterson, K. A.; Yousaf, K. E. Molecular core-valence correlation effects involving the post-d elements Ga-Rn: Benchmarks and new pseudopotential-based correlation consistent basis sets. *J. Chem. Phys.* **2010**, *133*, 174116.

(3) Dunning Jr., T. H.; Peterson, K. A.; Wilson, A. K. Gaussian basis sets for use in correlated molecular calculations. X. The atoms aluminum through argon revisited. *J. Chem. Phys.* **2001**, *114*, 9244.

(4) LaJohn, L.; Christiansen, P.; Ross, R.; Atashroo, T.; Ermler, W. Ab initio relativistic effective potentials with spin–orbit operators. III. Rb through Xe. *The Journal of chemical physics* **1987**, *87*, 2812–2824.

(5) Karton, A.; Martin, J. M. L. Comment on: "Estimating the Hartree–Fock limit from finite basis set calculations". *Theor. Chem. Acc.* **2006**, *115*, 330.

(6) Helgaker, T.; Klopper, W.; Koch, H.; Noga, J. Basis-set convergence of correlated calculations on water. *J. Chem. Phys.* **1997**, *106*, 9639.

(7) Nagy, P. R.; Kállay, M. Approaching the basis set limit of CCSD(T) energies for large molecules with local natural orbital coupled-cluster methods. *J. Chem. Theory Comput.* **2019**, *15*, 5275.

(8) Nagy, P. R. State-of-the-art local correlation methods enable accurate and affordable gold standard quantum chemistry up to a few hundred atoms. *Chem. Sci.* **2024**, *15*, 14556.

(9) Gyevi-Nagy, L.; Kállay, M.; Nagy, P. R. Integral-direct and parallel implementation of the CCSD(T) method: Algorithmic developments and large-scale applications. *J. Chem. Theory Comput.* **2020**, *16*, 366.

(10) Kállay, M.; Nagy, P. R.; Mester, D.; Rolik, Z.; Samu, G.; Csontos, J.; Csóka, J.; Szabó, P. B.; Gyevi-Nagy, L.; Hégely, B.; Ladjánszki, I.; Szegedy, L.; Ladóczki, B.; Petrov, K.; Farkas, M.; Mezei, P. D.; Ganyecz, Á. The MRCC program system: Accurate quantum chemistry from water to proteins. *J. Chem. Phys.* **2020**, *152*, 074107.

(11) Mester, D.; Nagy, P. R.; Csóka, J.; Gyevi-Nagy, L.; Szabó, P. B.; Horváth, R. A.; Petrov, K.; Hégely, B.; Ladóczki, B.; Samu, G.; Lőrincz, B. D.; Kállay, M. An overview of developments in the MRCC program system. *J. Phys. Chem. A* **2025**, *129*, 2086.

(12) Kállay, M.; Nagy, P. R.; Mester, D.; Gyevi-Nagy, L.; Csóka, J.; Szabó, P. B.; Rolik, Z.; Samu, G.; Csontos, J.; Hégely, B.; Ganyecz, Á.; Ladjánszki, I.; Szegedy, L.; Ladóczki, B.; Petrov, K.; Farkas, M.; Mezei, P. D.; Horváth, R. A. MRCC*, a quantum chemical program suite. See https://www.mrcc.hu/* **Accessed Dec 1, 2024**,

(13) Caldeweyher, E.; Ehlert, S.; Hansen, A.; Neugebauer, H.; Spicher, S.; Bannwarth, C.; Grimme, S. A generally applicable atomic-charge dependent London dispersion correction. *J. Chem. Phys.* **2019**, *150*, 154122.

(14) Grimme, S.; Antony, J.; Ehrlich, S.; Krieg, H. A consistent and accurate ab initio parametrization of density functional dispersion correction (DFT-D) for the 94 elements H-Pu. *J. Chem. Phys.* **2010**, *132*, 154104.

(15) Vydrov, O. A.; Van Voorhis, T. Nonlocal van der Waals density functional: The simpler the better. *J. Chem. Phys.* **2010**, *133*, 244103.

(16) Becke, A. D. Density-functional exchange-energy approximation with correct asymptotic-behavior. *Phys. Rev. A* **1988**, *38*, 3098.

(17) Perdew, J. P. Density-functional approximation for the correlation energy of the inhomogeneous electron gas. *Phys. Rev. B* **1986**, *33*, 8822.

(18) Lee, C.; Yang, W.; Parr, R. G. Development of the Colle–Salvetti correlation-energy formula into a functional of the electron density. *Phys. Rev. B* **1988**, *37*, 785.

(19) Perdew, J. P.; Chevary, J. A.; Vosko, S. H.; Jackson, K. A.; Pederson, M. R.; Singh, D. J.; Fiolhais, C. Atoms, molecules, solids and surfaces: Applications of the generalized gradient approximation for exchange and correlation. *Phys. Rev. B* **1992**, *46*, 6671.

(20) Perdew, J. P.; Burke, K.; Ernzerhof, M. Generalized Gradient Approximation Made Simple. *Phys. Rev. Lett.* **1996**, *77*, 3865.

(21) Sun, J.; Ruzsinszky, A.; Perdew, J. P. Strongly Constrained and Appropriately Normed Semilocal Density Functional. *Phys. Rev. Lett.* **2015**, *115*, 036402.

(22) Tao, J.; Perdew, J. P.; Staroverov, V. N.; Scuseria, G. E. Climbing the Density Functional Ladder: Nonempirical Meta-Generalized Gradient Approximation Designed for Molecules and Solids. *Phys. Rev. Lett.* **2003**, *91*, 146401.

(23) Mardirossian, N.; Head-Gordon, M. Mapping the genome of meta-generalized gradient approximation density functionals: The search for B97M-V. *J. Chem. Phys.* **2015**, *142*, 074111.

(24) Zhao, Y.; Truhlar, D. G. A new local density functional for main-group thermochemistry, transition metal bonding, thermochemical kinetics, and noncovalent interactions. *J. Chem. Phys.* **2006**, *125*, 194101.

(25) Staroverov, V. N.; Scuseria, G. E.; Tao, J.; Perdew, J. P. Comparative assessment of a new nonempirical density functional: Molecules and hydrogen-bonded complexes. *J. Chem. Phys.* **2003**, *119*, 12129.

(26) Stephens, P. J.; Devlin, F. J.; Chabalowski, C. F.; Frisch, M. J. Ab initio calculation

of vibrational absorption and circular dichroism spectra using density functional force fields. *J. Phys. Chem.* **1994**, *98*, 11623.

(27) Perdew, J. P.; Ernzerhof, M.; Burke, K. Rationale for mixing exact exchange with density functional approximations. *J. Chem. Phys.* **1996**, *105*, 9982.

(28) Yu, H. S.; He, X.; Li, S. L.; Truhlar, D. G. MN15: A Kohn–Sham global-hybrid exchange-correlation density functional with broad accuracy for multi-reference and single-reference systems and noncovalent interactions. *Chem. Sci.* **2016**, *7*, 5032.

(29) Becke, A. D. A new mixing of Hartree–Fock and local density-functional theories. *J. Chem. Phys.* **1993**, *98*, 1372.

(30) Zhao, Y.; Truhlar, D. G. The M06 suite of density functionals for main group thermochemistry, thermochemical kinetics, noncovalent interactions, excited states, and transition elements: two new functionals and systematic testing of four M06-class functionals and 12 other functionals. *Theor. Chem. Acc.* **2006**, *120*, 215.

(31) Chai, J.-D.; Head-Gordon, M. Systematic optimization of long-range corrected hybrid density functionals. *J. Chem. Phys.* **2008**, *128*, 084106.

(32) Chai, J.-D.; Head-Gordon, M. Long-range corrected double-hybrid density functionals. *J. Chem. Phys.* **2009**, *131*, 174105.

(33) Mardirossian, N.; Head-Gordon, M. ωB97X-V: A 10-parameter, range-separated hybrid, generalized gradient approximation density functional with nonlocal correlation, designed by a survival-of-the-fittest strategy. *Phys. Chem. Chem. Phys.* **2014**, *16*, 9904.

(34) Mardirossian, N.; Head-Gordon, M. ωB97M-V: A combinatorially optimized, range-separated hybrid, meta-GGA density functional with VV10 nonlocal correlation. *J. Chem. Phys.* **2016**, *144*, 214110.

(35) Vydrov, O. A.; Scuseria, G. E. Assessment of a long-range corrected hybrid functional. *J. Chem. Phys.* **2006**, *125*, 234109.

(36) Yanai, T.; Tew, D. P.; Handy, N. C. A new hybrid exchange-correlation functional using the Coulomb-attenuating method (CAM-B3LYP). *Chem. Phys. Lett.* **2004**, *393*, 51.

(37) Grimme, S. Semiempirical hybrid density functional with perturbative second-order correlation. *J. Chem. Phys.* **2006**, *124*, 034108.

(38) Kozuch, S.; Martin, J. M. L. DSD-PBEP86: in search of the best double-hybrid DFT with spin-component scaled MP2 and dispersion corrections. *Phys. Chem. Chem. Phys.* **2011**, *13*, 20104.

(39) Santra, G.; Sylvetsky, N.; Martin, J. M. L. Minimally Empirical Double-Hybrid Functionals Trained against the GMTKN55 Database: revDSD-PBEP86-D4, revDOD-PBE-D4 and DOD-SCAN-D4. *J. Phys. Chem. A* **2019**, *123*, 5129.

(40) Mezei, P. D.; Csonka, G. I.; Ruzsinszky, A.; Kállay, M. Construction and Application of a New Dual-Hybrid Random Phase Approximation. *J. Chem. Theory Comput.* **2015**, *11*, 4615.

(41) Santra, G.; Semidalas, E.; Martin, J. M. L. Exploring avenues beyond revised DSD Functionals: II. Random-Phase Approximation and scaled MP3 corrections. *J. Phys. Chem. A* **2021**, *125*, 4628.

(42) Weigend, F.; Ahlrichs, R. Balanced basis sets of split valence, triple zeta valence and quadruple zeta valence quality for H to Rn: Design and assessment of accuracy. *Phys. Chem. Chem. Phys.* **2005**, *7*, 3297.

(43) Frisch, M. J.; Trucks, G. W.; Schlegel, H. B.; Scuseria, G. E.; Robb, M. A.; Cheeseman, J. R.; Scalmani, G.; Barone, V.; Mennucci, B.; Petersson, G. A.; Nakatsuji, H.;

Caricato, M.; Li, X.; Hratchian, H. P.; Izmaylov, A. F.; Bloino, J.; Zheng, G.; Sonnenberg, J. L.; Hada, M.; Ehara, M.; Toyota, K.; Fukuda, R.; Hasegawa, J.; Ishida, M.; Nakajima, T.; Honda, Y.; Kitao, O.; Nakai, H.; Vreven, T.; Montgomery, J. A.; Jr.; Peralta, J. E.; Ogliaro, F.; Bearpark, M.; Heyd, J. J.; Brothers, E.; Kudin, K. N.; Staroverov, V. N.; Keith, T.; Kobayashi, R.; Normand, J.; Raghavachari, K.; Rendell, A.; Burant, J. C.; Iyengar, S. S.; Tomasi, J.; Cossi, M.; Rega, N.; Millam, J. M.; Klene, M.; Knox, J. E.; Cross, J. B.; Bakken, V.; Adamo, C.; Jaramillo, J.; Gomperts, R.; Stratmann, R. E.; Yazyev, O.; Austin, A. J.; Cammi, R.; Pomelli, C.; Ochterski, J. W.; Martin, R. L.; Morokuma, K.; Zakrzewski, V. G.; Voth, G. A.; Salvador, P.; Dannenberg, J. J.; Dapprich, S.; Daniels, A. D.; Farkas, O.; Foresman, J. B.; Ortiz, J. V.; Cioslowski, J.; ; Fox, D. J. Gaussian 16 Revision C.01. 2016; Gaussian, Inc., Wallingford CT.

(44) Csókás, D.; Siitonen, J. H.; Pihko, P. M.; Pápai, I. Conformationally Locked Pyramidality Explains the Diastereoselectivity in the Methylation of trans-Fused Butyrolactones. *Org. Lett.* **2020**, *22*, 4597–4601.

(45) Földes, T.; Madarász, Á.; Révész, Á.; Dobi, Z.; Varga, S.; Hamza, A.; Nagy, P. R.; Pihko, P. M.; Pápai, I. Stereocontrol in Diphenylprolinol Silyl Ether Catalyzed Michael Additions: Steric Shielding or Curtin–Hammett Scenario? *J. Am. Chem. Soc.* **2017**, *139*, 17052.

(46) Mayer, I. Charge, bond order and valence in the ab initio SCF theory. *Chem. Phys. Lett.* **1983**, *97*, 270.

(47) Kozuch, S.; Martin, J. M. L. Halogen Bonds: Benchmarks and Theoretical Analysis. *J. Chem. Theory Comput.* **2013**, *9*, 1918.

(48) Song, S.; Vučković, S.; Sim, E.; Burke, K. Density Sensitivity of Empirical Functionals. *J. Phys. Chem. Lett.* **2021**, *12*, 800.

(49) Anderson, L. N.; Aquino, F. W.; Raeber, A. E.; Chen, X.; Wong, B. M. Halogen Bonding Interactions: Revised Benchmarks and a New Assessment of Exchange vs Dispersion. *J. Chem. Theory Comput.* **2018**, *14*, 180–190.

(50) Stoychev, G. L.; Auer, A. A.; Neese, F. Automatic Generation of Auxiliary Basis Sets. *J. Chem. Theory Comput.* **2017**, *13*, 554.
